# Perioperative oxygen therapy: an overview of systematic reviews and meta-analyses

**DOI:** 10.1016/j.bja.2025.04.020

**Published:** 2025-06-06

**Authors:** Adel Elfeky, Yen-Fu Chen, Amy Grove, Keith Couper, Rachel Court, Sara Tomassini, Anna Wilson, Amy Hooper, Alexandra Buckle, Sharvari Vadeyar, Marion Thompson, Olalekan Uthman, Joyce Yeung

**Affiliations:** 1 Warwick Clinical Trials Unit, Warwick Medical School, University of Warwick, Coventry, UK; 2 University Hospitals Birmingham NHS Foundation Trust, Birmingham, UK

**Keywords:** meta-analysis, overview review, oxygen therapy, surgery, surgical wound infection, umbrella review

## Abstract

**Background:**

Perioperative oxygen is routinely used, but evidence of its clinical impact remains inconsistent, leading to variable practice. The aim of this work was to provide a comprehensive overview of the effectiveness of perioperative oxygen therapy strategies.

**Methods:**

We searched multiple databases for systematic reviews comparing perioperative oxygen strategies. Two reviewers independently extracted data. The co-primary outcomes were surgical site infection (SSI) and mortality. We classified systematic reviews with the largest number of trials as anchoring reviews. We updated anchoring reviews with data from more recent RCTs. We assessed the risk of bias of the anchoring reviews using the ROBIS tool, updated meta-analyses and subgroup analyses, and undertook exploratory meta-regression. We assessed the certainty in evidence using GRADE framework and conducted trial sequential analysis.

**Results:**

Fifty-nine systematic reviews met the inclusion criteria, from which five anchoring reviews were selected. Perioperative high fraction of inspired oxygen (Fio_2_, 80%), compared with a low Fio_2_ (30–35%), may reduce the incidence of SSI slightly (risk ratio [RR] 0.87, 95% confidence interval [CI] 0.76–1.01; risk difference [RD] 1.6% lower, 3% lower to 0.1% higher), but the evidence is very uncertain. High inspired oxygen may result in little to no difference in mortality (RR 1.17, 95% CI 0.77–1.78, RD 0.3% higher, 0.4% lower to 1.3% higher), based on low-certainty evidence. The evidence suggests that high Fio_2_ results in a large increase in the incidence of atelectasis (RR 1.47, 95% CI 1.20–1.79, RD 6.5% higher, 2.8% higher to 10.9% higher, low-certainty evidence). Postoperative noninvasive ventilation (NIV) does not reduce mortality compared with conventional oxygen therapy (COT; RR 0.91, 95% CI 0.62–1.32, RD 0.1% lower, 0.6% lower to 0.5% higher), based on high-certainty evidence. Low-certainty evidence suggests that postoperative high-flow nasal oxygen (HFNO) compared with COT does not reduce mortality (RR 0.78, 95% CI 0.27–2.24, RD 0.4% lower, 1.4% lower to 2.4% higher). Low- to very low-certainty evidence indicates that postoperative NIV and HFNO may reduce some of the pulmonary adverse events compared with COT. Trial sequential analyses showed that further studies are required to determine which perioperative oxygen strategy is most clinically and cost effective.

**Conclusions:**

We did not find evidence to support routine use of high inspired oxygen to reduce surgical site infection and improve patient outcomes. A small reduction in surgical site infection associated with high Fio_2_ cannot be ruled out and possible effect modifiers require further investigation. Existing evidence favours postoperative noninvasive ventilation and high-flow nasal oxygen over conventional oxygen therapy, but the low to very low certainty of evidence limits our confidence in the findings.

**Systematic review protocol:**

PROSPERO CRD42021272361.

**Editor's key points**

• Perioperative oxygen is routinely used, but evidence of its clinical impact remains inconsistent, leading to variable practice.
• Administering 80% oxygen perioperatively appears to have minimal effect on reducing surgical site infections and can increase the risk of atelectasis. Evidence for postoperative noninvasive ventilation and high-flow nasal oxygen suggests potential reductions in pulmonary complications, though data quality is low.
• Research is needed to establish optimal perioperative and postoperative oxygen strategies to support evidence-based clinical decision-making.

More than 310 million operations are carried out globally each year.^1^ The ubiquitous use of oxygen therapy across the perioperative care pathway is driven by the desire to prevent hypoxaemia. Oxygen therapy is also used to prevent postoperative complications, such as surgical site infections (SSIs) and postoperative pulmonary complications (PPCs).

SSIs complicate up to 20% of operations, and represent the third most common healthcare-associated infection in the UK.^2^ SSIs delay recovery, prolong hospital stays, and often require treatment with antimicrobials. The World Health Organization (WHO) has proposed the use of a high concentration of intraoperative oxygen (80%) as a strategy to reduce SSIs. Concerns as to the reliability of the evidence and potential harms of this recommendation has led to widespread variability in clinical guidelines and practice.^3^^,^^4^ There is similarly uncertainty and variability in practice in relation to other perioperative oxygen strategies, such as noninvasive ventilation (NIV) strategies in the immediate postoperative period.

Perioperative oxygen strategies potentially offer a cheap and rapidly implementable way to reduce surgical morbidity and mortality. However, strategies must be proved to be clinically effective before they are recommended into routine practice. To facilitate research prioritisation in this area, the UK National Institute for Health and Care Research commissioned this review to identify for which types of surgery, at which stages of care, in which subgroups of patients, and delivered under what conditions are different types of perioperative oxygen therapy clinically effective.

## Methods

We prospectively registered this review on PROSPERO (CRD42021272361) and have followed the Preferred Reporting Items for Overviews of Reviews (PRIOR) guidelines.^5^ The full protocol is published elsewhere.^6^ This project was an evidence synthesis of publicly accessible data and therefore did not require ethical approval.

### Study selection criteria

Details of the inclusion and exclusion criteria are highlighted in Table 1.

**Table 1:** Overview of inclusion and exclusion criteria.

Criteria	Inclusion	Exclusion
Population	Hospitalised patients undergoing surgical procedures (where patients would normally be provided with anaesthesia by either an anaesthetist or a qualified anaesthetic practitioner) of any age group, and surgical specialty at any stage of the surgical pathway including preoperative, intraoperative, and postoperative periods.	
Intervention	Perioperative oxygen therapy, defined as oxygenation strategy where the primary purpose of the intervention is to optimise oxygenation/oxygen delivery, with the aim of preventing hypoxaemia or reducing complications during the perioperative period.	We excluded systematic reviews that primarily focus on intraoperative ventilation strategies (e.g. ventilatory rate, pressure and volume settings), hyperbaric oxygen therapy, and extracorporeal life support.^7^ Reviews that examine preoxygenation strategies during tracheal intubation are excluded.
Comparator	Any comparator or control.	
Outcomes	Primary outcomes: 1- Surgical site infection (SSI) within 30 days of follow-up after surgery: we followed definitions of the US Centers for Disease Control and Prevention (CDC) where possible. The CDC defines SSI as an infection related to a surgical procedure that occurs near the surgical site within 30 days after surgery (or up to 90 days after surgery where an implant is involved).^8^ Reviews (and RCTs included in the reviews) that have adopted other definitions were included and examined if meeting other inclusion criteria, but differences in the outcome definitions were recorded and highlighted. 2- All-cause mortality within 30-days after surgery. Secondary outcomes: 1- Postoperative pulmonary complications (PPCs): defined according to the most recent consensus definition of PPC^9^ as composite of respiratory diagnoses: (i) atelectasis detected on computed tomography or chest radiograph, (ii) pneumonia using US CDC criteria, (iii) acute respiratory distress syndrome (ARDS) using Berlin consensus definition, (iv) pulmonary aspiration (clear clinical history and radiological evidence). 2- Postoperative respiratory failure: including ARDS defined using Berlin consensus definition^10^ and need for mechanical ventilation. Definitions for the above outcomes are recommended by the StEP-COMPAC Group.^9^ We accepted similar outcomes defined differently in previous studies. Differences in the outcome definitions were recorded and highlighted. 3- Mortality up to the longest point of postoperative follow-up. 4- Length of hospital stay: the number of days from the day of surgery to hospital discharge or death. 5- ICU admission: unplanned admission to ICU within 14 days of surgery. 6- Quality of life. Further outcomes not listed above but are identified during the review and considered important by the Advisory Group were examined. Any *post hoc* addition of such outcomes is explicitly stated in our report.	
Study design	Systematic reviews and meta-analyses of RCTs that examine the use of perioperative oxygen therapy. We included systematic reviews that included both randomised and non-randomised studies if evidence summarised from RCTs was reported separately. Systematic reviews must have fulfilled a minimum of four methodological criteria as defined by Centre for Reviews and Dissemination, University of York, guidance to be included.^11^ Specifically, reviews must have reported inclusion/exclusion criteria of studies, an adequate search strategy, synthesis of included studies, description of and quality assessment of included studies.	

### Information sources and search strategy

Our information specialist (RC) searched MEDLINE, Embase, the Cochrane Database of Systematic Reviews, Epistemonikos,^12^ PROSPERO,^13^ the International HTA Database - INAHTA, the DARE archives, and ECRI Guidelines Trust database^14^ from inception to September 2021 (Supplementary material 1). Searches were not limited by date or publishing language. Non-English language articles were translated into English. We reviewed the reference lists of relevant articles and used citation search facilities provided by the Web of Knowledge to check records of papers citing our anchoring reviews.

To ensure that emerging trial evidence was covered, we also searched for recently published or ongoing/planned RCTs in the Cochrane CENTRAL database from April 2018 to August 2023. This date was determined by when the searches were performed in recent, relevant systematic reviews identified in the first set of searches.

### Study selection and mapping

#### Initial review selection

**Fig 1:**
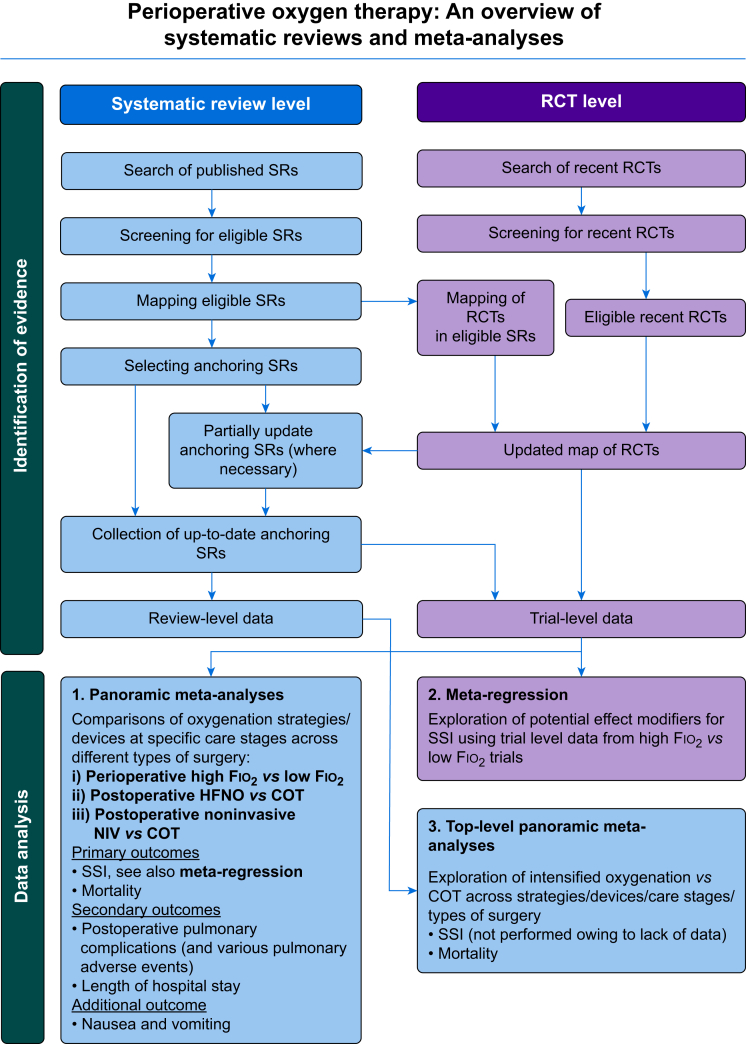
Overview schema. COT, conventional oxygen therapy; Fio_2_, fraction of inspired oxygen; HFNO, high-flow nasal oxygen; NIV, noninvasive ventilation; SR, systematic review; SSI, surgical site infection.

Titles and abstracts of records retrieved were screened by two reviewers independently (two of AE/ AW/ AH/ ST) and disagreement was resolved by discussion or if needed with the input of a third senior reviewer (one of JY/ KC/ YC/ AG). Full-text articles considered potentially meeting inclusion criteria were assessed for inclusion by two reviewers independently and disagreements resolved as above. Figure 1 illustrates the overview schema. We used Evidence for Policy and Practice Information (EPPI)-Reviewer 4 software to manage records and data throughout the review.^15^

#### Study mapping

Each review was mapped according to types of surgery, stages of perioperative care, and comparisons made. Three major groups of comparisons emerged among the identified systematic reviews: (1) perioperative high *vs* low fraction of inspired oxygen (Fio_2_); (2) NIV (including CPAP and other forms of NIV but excluding high-flow nasal oxygen [HFNO]) *vs* conventional oxygen therapy (COT); and (3) HFNO *vs* COT.

Findings are presented using this categorisation. We also mapped all RCTs included in the systematic reviews to gauge the extent of overlap between reviews and to ensure that no double counting of evidence occurred when undertaking further quantitative analyses. Where reviews covering largely overlapping topic areas and RCTs are identified, a single review with the most comprehensive coverage of literature, the highest methodological quality, or both, as judged by the ROBIS tool,^16^ was selected (through deliberation within the review team) as the anchoring review, to which we added evidence from recent RCTs in updated meta-analyses that we undertook (described below). RCTs published since the searches in the anchoring reviews were performed and that were judged to be eligible were added to an RCT map, which is a list of all eligible RCTs identified either in relevant systematic reviews that we found or through the additional searches of recent RCTs that we conducted (Supplementary material 2). We became aware during the preparation of the overview that five trials by one research group have been retracted and a study by Myles and colleagues^17^ found evidence that data integrity was compromised in 38 out of 40 analysed papers from the research group. To eliminate all doubt, we proactively excluded all trials by that research group from our analyses.

Anchoring reviews were checked against the RCT map to assess if any important RCTs not included in the anchoring reviews warrant addition, thus updating relevant analyses in the anchoring reviews (where this was considered necessary and feasible).

### Data extraction

Two reviewers (two of AE/ AW/ AH/ ST) independently extracted data from each chosen anchoring review. This included number of RCTs included, number of patients, intervention and comparator, type of surgery, all clinical outcomes and adverse events reported, pooled results for the primary and secondary outcomes listed above and for additional outcomes judged to be important by the research team and Advisory Panel, results of subgroup and sensitivity analyses, and certainty of evidence.

### Risk of bias assessment

Risk of bias for each of the anchoring reviews was assessed using the ROBIS tool, focusing on phase 2 (identifying concerns with the review process) of the assessment.^16^ New RCTs included in our updated meta-analyses of the anchoring reviews were assessed using the Cochrane ROB2 tool for each relevant outcome.^18^ Where an anchoring review used different methodological approaches to assessing the risk of bias that could impact on the comparability of findings between the reviews, the risk of bias for included RCTs was reassessed for each relevant outcome using the ROB2 tool. All assessments were conducted by two reviewers independently (ST, AE) with conflicts resolved through discussion or consultation with a third reviewer (YC).

### Data synthesis

#### Panoramic meta-analysis

The term ‘panoramic meta-analysis’ was originally used to refer to a meta-analysis that evaluates the effectiveness of an intervention across different indications (e.g. type of surgery), usually using pooled results from systematic reviews focusing on different indications with an intention to test a hypothesis of a generic effect of an intervention across different indications.^19^ In this overview, we tested the hypothesis that delivery of a higher concentration of oxygen perioperatively at various time through various devices could reduce SSI across different types of surgery. We used trial-level data rather than review-level data and therefore the panoramic meta-analyses that we report in this paper are equivalent to meta-analyses with subgroups defined by types of surgery. The subgroups of surgery were determined before the commencement of analyses by the project team in consultation with the advisory panel: abdominal surgery *vs* caesarean section *vs* other surgery. Additional pre-specified subgroups that we investigated (where data available) were: oxygen delivery through tracheal intubation *vs* face masks or nasal cannula; use of nitrous oxide as carrier gas for anaesthesia in the control group *vs* no use of nitrous oxide; trials with low risk of bias *vs* trials with high or unclear risk of bias; and urgency of the surgery (elective *vs* emergency *vs* mixed).

We also updated additional subgroup analyses reported in the anchoring reviews where the main meta-analyses were updated. Risk ratios (RRs) with 95% confidence intervals (CIs) were reported and presented in forest plots for each meta-analysis. A random effects model was used to account for heterogeneity that cannot readily be explained.^20^ Inconsistency across different studies within individual subgroups and across different subgroups was assessed using the I^2,^statistic.^21^

Two ‘top-level’ panoramic meta-analyses were planned for the two primary outcomes (SSI and 30-day all-cause mortality) to further aggregate the data across the three main comparisons, with pooled data from individual panoramic meta-analysis as the unit of analysis. The purpose was to explore the compatibility of all relevant evidence included in this overview with the hypothesis that perioperative exposure to higher concentrations of oxygen reduces SSI; and to gauge the overall benefit/risk of delivering more oxygen perioperatively on postoperative 30-day all-cause mortality.

#### Additional analyses

To explore more than one potential effect modifier for SSI simultaneously, a meta-regression was planned and performed. No meta-regression was planned for the mortality outcome as the number of studies reporting mortality and the number of death events was expected to be substantially smaller than those of SSI.

Trial sequential analysis was performed to address the risk of type I and II errors associated with sparse data, repeated significance testing, or both, which can affect cumulative meta-analysis.^22^^,^^23^ The required information size was estimated assuming a relative risk reduction (RRR) of 20% with 90% power and an alpha error of 5%. RRR was estimated using the event rates of both arms with model variance-based heterogeneity correction.

Meta-analyses and meta-regression were performed in STATA using *meta* and *metareg* commands.^24^ Trial sequential analyses were performed using trial sequential analysis software version 0.9.5.10 (The Copenhagen Trial Unit, Centre for Clinical Intervention Research, Copenhagen, Denmark).

We created funnel plots to examine the possibility of publication bias and small-study effects for meta-analyses with at least 10 included studies. Finally, the GRADE approach was used to assess the certainty of evidence for each outcome within each (updated) anchoring review.^25^ Judgements on certainty of evidence were taken directly from the anchoring reviews if reported (for outcomes which did not require updating); otherwise, the GRADE assessment for the updated anchoring reviews was undertaken by two researchers independently (AE and YC) using GRADEpro GDT software^26^ and reported using the informative statements guidance.^27^ When communicating an effect using these statements, we focused on the best estimate and on the certainty in that estimate which considers multiple factors.

GRADE now suggests an aligning approach that relies on thresholds and CIs of the absolute effect as a primary criterion for imprecision rating. In the partially contextualised approach,^28^ one would judge whether the effect of an intervention on a specific outcome (expressed in absolute terms) falls in a category of magnitude of effect (i.e. trivial or none, small, moderate, or large effects).^29^ When rating the certainty of evidence under this approach, the ratings represent the certainty that the true effect lies within the thresholds of one of these four categories of magnitude of effect or beyond the threshold for large effects. The effects are a result of combining absolute estimates and the importance (value) of these outcomes. Specifically, when assessing imprecision, the extent of rating down depends on the number of thresholds being crossed by the CI. Conceptually, we rated down by one level when the CI for the absolute effect crossed one of the thresholds across the range of categories of magnitude of effect. We rated down by two or three levels when the CI for the absolute effect crosses two or three of the thresholds across the range of categories of magnitude of effect, respectively.

### GRADE informative statements to communicate the findings of the overview

We followed GRADE's informative statements to communicate results of the review.^27^ Two aspects of a result were used to communicate the review's findings: the size or magnitude of the effect and the level of certainty of the evidence. Two factors are crucially important when determining the size of effect. The first involves calculating and using absolute effects instead of using relative effects that can often be misleading. For instance, consider RR of 0.82, or 18% relative reduction in SSI. If on the one hand, the baseline risk of SSI is 40/1000, the RR 0.82 would translate into seven fewer infections per 1000 patients in absolute terms, which might be considered a trivial effect. If on the other hand, the baseline risk is much higher at 400/1000, the same RR would translate into 72 fewer infections per 1000 patients, which would be considered a large effect. The second is identifying threshold values in absolute terms which would define the clinical importance of the outcome. We have defined small, moderate, and large absolute risk difference (RD) to be 1% (10/1000), 2.5% (25/1000), and 5% (50/1000), respectively in consultation with our advisory panel.

The statements communicate the size of the effect based on the point estimate in a meta-analysis, as it is the most likely value of true effect given available data. CIs are taken into account when determining the certainty of evidence. CIs are calculated based on factors such as sample sizes and variance between or within studies. Whether the estimate is sufficiently precise is determined by comparing the CIs against the thresholds for large, moderate, and small effects. Wider CIs spanning across one or more of these thresholds indicate that the estimate may not be sufficiently precise and would result in downgrading of the certainty of evidence. In addition to imprecision, GRADE assessment factors in the risk of bias in the included studies; inconsistency and indirectness of evidence, and the risk of publication bias. The final list of informative statements as recommended by GRADE^27^ is shown in Supplementary material 3.

We recognise that readers and clinical communities may be looking for more definitive statements and may feel some of the wording recommended by GRADE and used by us in this paper is ambiguous. However, it is important to emphasise that the GRADE informative statements deliberately use the recommended wording to convey different levels of (un)certainty related to the evidence in a consistent way, which was also our motivation to adopt these informative statements.

### Stakeholder involvement

Our advisory panel represented key stakeholders involved in the use of perioperative oxygen, including patients and a multidisciplinary group of clinical specialists with expertise in anaesthesia, critical care, surgery, and physiotherapy. In addition, a patient representative (MT) worked closely with the research team throughout the project. We worked with advisory panel members on three occasions. Each meeting included pre-meeting background information, presentation from the study team, and minutes were circulated to ensure comments were fully captured from members. Panel members reviewed our synthesis strategy and interpretation of evidence to guide the production of clinically relevant recommendations and conclusions.

## Results

### Search results

**Fig 2:**
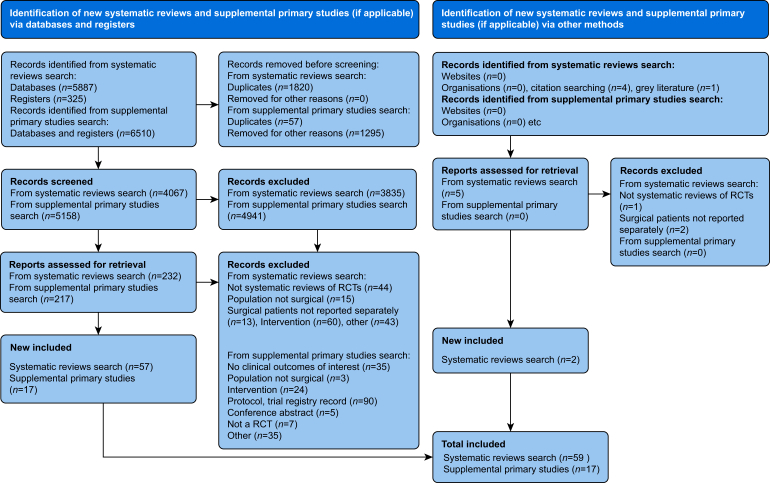
PRIOR flow diagram; in relation to Preferred Reporting Items for Overviews of Reviews.

In total, 4067 titles and abstracts were screened after deduplication and 232 records remained for full-text screening. Of these, a further 175 were excluded, and 59 reviews were selected for final inclusion. Seventeen additional RCTs were identified and were used to update the analyses in relevant anchoring reviews. The PRIOR diagram describing each of these stages is presented in Figure 2.

### Mapping of included reviews

We included 59 systematic reviews in this overview. Twenty-nine reviews compared high *vs* low Fio_2_^30^, ^31^, ^32^, ^33^, ^34^, ^35^, ^36^, ^37^, ^38^, ^39^, ^40^, ^41^, ^42^, ^43^, ^44^, ^45^, ^46^, ^47^, ^48^, ^49^, ^50^, ^51^, ^52^, ^53^, ^54^, ^55^, ^56^, ^57^, ^58^; 13 reviews compared HFNO *vs* COT,^59^, ^60^, ^61^, ^62^, ^63^, ^64^, ^65^, ^66^, ^67^, ^68^, ^69^, ^70^ and 17 reviews compared NIV *vs* COT.^71^, ^72^, ^73^, ^74^, ^75^, ^76^, ^77^, ^78^, ^79^, ^80^, ^81^, ^82^, ^83^, ^84^, ^85^, ^86^, ^87^

Considerable overlap between the primary studies included in the systematic reviews was observed (Supplementary material 2). All the included reviews examined the effectiveness of different oxygenation strategies in the adult population. None attempted to synthesise the evidence in the paediatric population.

No systematic reviews assessed the effectiveness of oxygenation strategies at the preoperative stage. Likewise, no evidence was identified on the effectiveness of postoperative administration of high Fio_2_ on patient outcomes compared with low Fio_2_, via facemask or nasal cannulae.

We selected five anchoring reviews,^30^, ^31^, ^32^^,^^59^^,^^87^ which provided comprehensive up-to-date evidence on the effectiveness of perioperative oxygen therapy. Three anchoring reviews assessed the effectiveness of high (80%) *vs* low (30–35%) Fio_2_ in patients undergoing surgery.^30^, ^31^, ^32^ de Jonge and colleagues^30^ focused on the incidence of SSI. Lim and colleagues^31^ examined other postoperative clinical outcomes in patients undergoing non-thoracic surgery under general anaesthesia. Markwei and colleagues^32^ assessed the incidence of postoperative nausea, vomiting, or both; this outcome was not pre-specified in our protocol but was considered as an important outcome for patients by our advisory panel.

The remaining two anchoring reviews examined the effectiveness of postoperative noninvasive respiratory support. Hui and colleagues^87^ assessed the effectiveness of routine postoperative noninvasive respiratory support after elective surgery. The review covered both NIV and HFNO compared with COT, but we used this anchoring review only for evidence related to NIV. Chaudhuri and colleagues^59^ assessed the effect of HFNO in the immediate postoperative period compared with COT in surgical patients. This review was chosen to provide evidence on HFNO *vs* COT given its more comprehensive coverage than Hui and colleagues for this comparison.

Table 2 outlines the characteristics of chosen anchoring reviews and further analyses undertaken in this overview. Supplementary material 4 highlights the characteristics of non-anchoring reviews. Characteristics of additional RCTs identified from the updated search are provided in Supplementary material 5.

**Table 2:** Characteristics of anchoring reviews and further analyses undertaken in this overview. ARDS, acute respiratory distress syndrome; COT, conventional oxygen therapy; CPAP, continuous positive airway pressure; Fio_2_, fraction of inspired oxygen; HFNO, high-flow nasal oxygen; LOS, length of stay; NIV, noninvasive ventilation; PPCs, postoperative pulmonary complications.

Anchoring reviews					Evidence included and further analyses undertaken in this overview
Review ID and care stage	RCTs included (participant included)	Patient population (age group)	Intervention/comparator	Outcomes evaluated	Updating of original meta-analyses and additional analyses performed
**de Jonge 2019** Intraoperative and postoperative	21 RCTs (17 RCTs meta-analysed, *n*=7817)	Patients undergoing surgical procedures (adults)	High Fio_2_ (80%)/low Fio_2_ (30–35%)	Surgical site infection	New studies were added Subgroup analyses: type of surgery, intubation *vs* not, use of nitrous oxide in the control group, risk of bias of the RCT, urgency of surgery Meta-regression
**Lim 2021** Intraoperative and postoperative	26 RCTs (*n*=4991)	Patients undergoing non-thoracic surgery (adults)	High Fio_2_ (80–100%)/low Fio_2_ (30–40%)	Primary outcome: mortality within 30 days Secondary outcomes: PPCs, pneumonia, respiratory failure, atelectasis, ICU admissions, and hospital LOS	New studies were added
**Markwei 2023** Intraoperative	15 RCTs (10 RCTs meta-analysed, *n*=6773)	Patients undergoing surgery (adults)	80% *vs* 30% Fio_2_	The incidence of postoperative nausea and vomiting	No studies were added
**Hui 2022** Postoperative	38 RCTs (*n*=9782)	Patients undergoing major elective surgery (adults).	Noninvasive respiratory support (including CPAP, other forms of NIV, and HFNO)/standard postoperative care (COT).	Primary outcome: pneumonia Secondary outcome: PPCs Other outcomes: mortality, ARDS, pulmonary aspiration, reintubation, unplanned ICU admissions, hospital LOS	No studies were added. Results were reanalysed to focus on NIV trials. Subgroup analysis (for mortality and pneumonia): type of surgery
**Chaudhuri 2020** Postoperative	11 RCTs (*n*=2201)	Patients undergoing surgical procedures (adults)	HFNO/COT or NIV	Mortality, reintubation, escalation of respiratory support, postoperative hypoxemia, treatment complications, ICU, and hospital LOS	New studies were added to the review Subgroup analysis (for reintubation and escalation of respiratory support): type of surgery, risk of PPCs, BMI

### Methodological quality of included reviews

The results of the ROBIS assessment, focusing on the four domains examined in phase 2 of the assessment, are provided in Supplementary material 6.

All reviews achieved a low risk of bias rating for specification of study eligibility criteria. Three reviews were judged to be at low risk of bias for the identification and selection of studies^30^^,^^31^^,^^87^; one review was rated as unclear^32^ and one was at high risk.^59^ All reviews achieved a low risk of bias rating for methods used to collect data and appraise studies and for synthesis and findings.

### Risk of bias in additional RCTs

Nine of the 17 newly identified RCTs were considered at low risk of bias for all risk of bias domains.^88^, ^89^, ^90^, ^91^, ^92^, ^93^, ^94^, ^95^, ^96^ Six RCTs had some cause for concern.^97^, ^98^, ^99^, ^100^, ^101^, ^102^ Two trials reporting on SSI were considered at high risk of bias.^103^^,^^104^ The domain most frequently assessed at high risk or of some concern of bias was deviations from the intended interventions.

Risk of bias judgements for important outcomes across comparisons are presented in Supplementary material 7.

The anchoring review (de Jonge and colleagues,^30^ 2019) for SSI assessed risk of bias in included RCTs using the original Cochrane risk of bias tool. Therefore, we reassessed the risk of bias for RCTs included in the review using the revised RoB2 tool. Details of RoB2 ratings are provided in Supplementary material 8.

### Findings of data synthesis

Findings are presented for the three main comparisons: high *vs* low Fio_2_, NIV *vs* COT, and HFNO *vs* COT. Within each comparison, we firstly present latest pooled estimates for the two primary outcomes (SSI and mortality), followed by our pre-specified secondary outcomes and other relevant outcomes reported in the original anchoring reviews. Findings from subgroup and other analyses were reported alongside respective outcomes.

**Fig 3:**
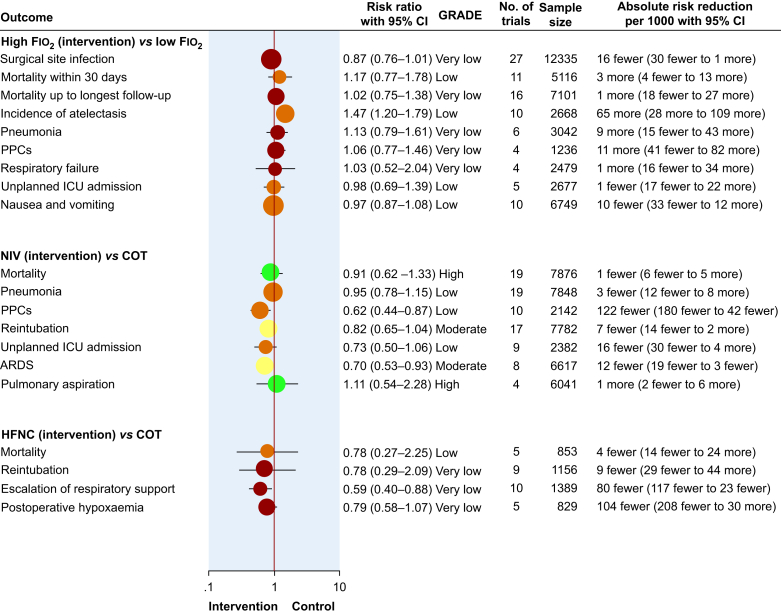
Evidence map of systematic reviews and meta-analyses. *The pooled effect estimates are presented both in relative and absolute terms, with the centre of the bubbles corresponding to the point estimate of the pooled relative risks. The size of the bubble reflects the volume of evidence (total sample size) for each pooled estimate. The overall certainty in evidence is rated according to the GRADE framework and is colour-coded for the bubbles (green*, *high certainty; yellow*, *moderate certainty; amber*, *low certainty; red*, *very low certainty).* ARDS, acute respiratory distress syndrome; CI, confidence interval; COT, conventional oxygen therapy; Fio_2_, fraction of inspired oxygen; HFNO, high-flow nasal oxygen; NIV, noninvasive ventilation; PPCs, postoperative pulmonary complications.

A summary of findings for key outcomes, including the latest pooled point estimates and GRADE assessment for certainty of evidence, can be found in the evidence map shown in Figure 3.

### Perioperative high *vs* low fraction of inspired oxygen

#### Surgical site infection

Our updated meta-analysis included 27 trials with 12 335 participants. Ten of these were newly identified since the publication of the review by de Jonge and colleagues^30^ in 2019.

**Fig 4:**
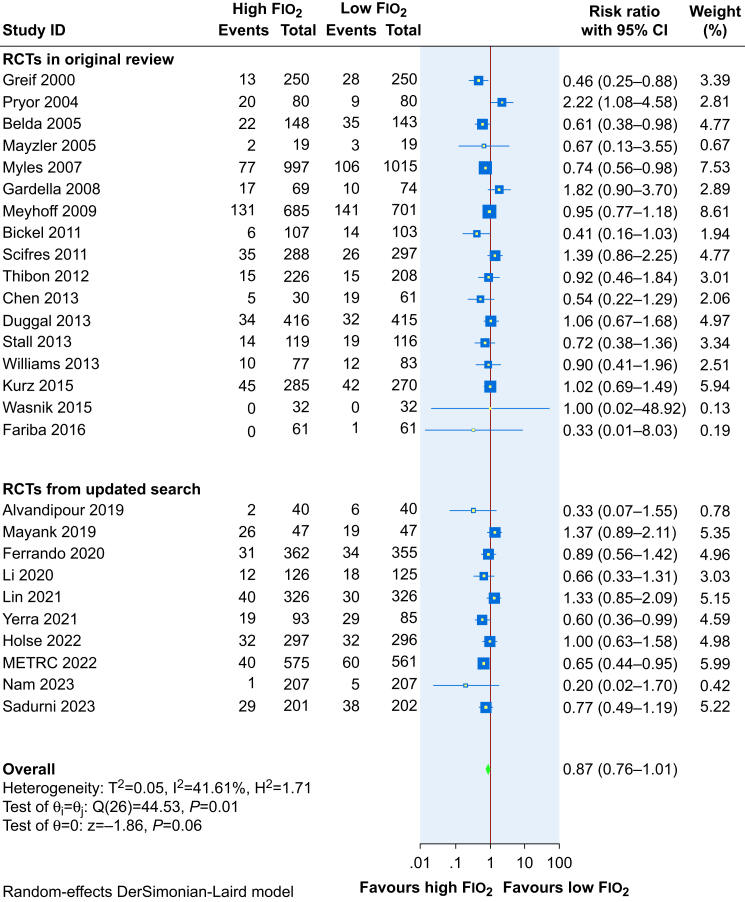
Effect of 80% Fio_2_ on SSI when compared with 30–35% Fio_2_. CI, confidence interval; Fio_2_, fraction of inspired oxygen.

**Fig 5:**
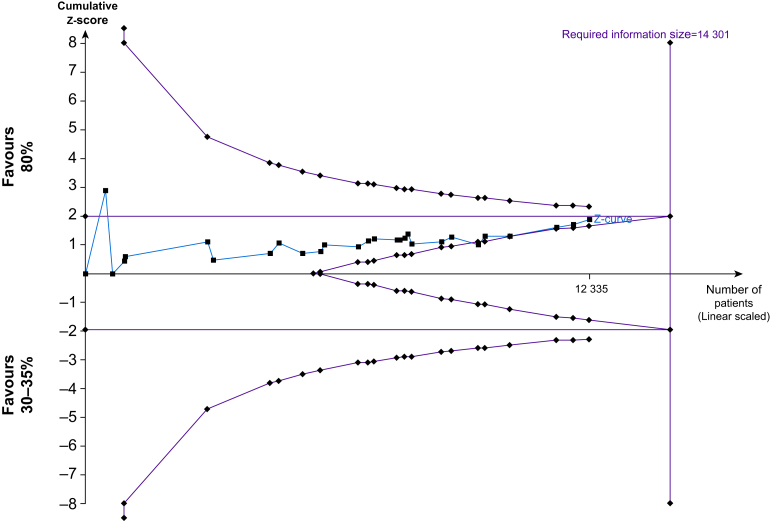
Trial sequential analysis of surgical site infection in all trials included in the meta-analysis.

The updated meta-analysis (Fig. 4) suggests that high (80%) Fio_2_ may reduce the incidence of SSI compared with low (30–35%) Fio_2_ (RR 0.87, 95% CI 0.76–1.01, RD 1.6% reduction, 95% CI 3% reduction to 0.1% increase), but the evidence is very uncertain. Trial sequential analysis for SSI showed that the number of patients evaluated did not surpass the required information size (*n*=14 301). The cumulative Z-curve crossed neither the conventional nor the TSA boundary for benefit or harm and trial sequential monitoring boundaries for futility (Fig. 5). This indicated that, considering repetitive testing, the evidence was not sufficient to refute a 20% relative risk increase (RRI) or a 20% RRR for the benefit or harm of higher *vs* lower Fio_2_.

The trial sequential analysis for SSI in trials at low risk of bias (Supplementary material 9) indicated that 3537 more patients will be needed to make a definitive conclusion on the benefits of high Fio_2_. The cumulative Z-curve crossed the boundaries of conventional significance and the trial sequential monitoring boundaries for benefit.

The funnel plot did not reveal an asymmetrical appearance suggesting no clear sign of small-study effects (Supplementary material 10); regression-based Egger's test was not statistically significant (*P*=0.14)

#### Surgical site infection: subgroup analyses, meta-regression, and sensitivity analysis

Table 3 summarises the findings of subgroup analyses and univariable meta-regression for the SSI outcome (forest plots are presented in Supplementary material 11). The analyses suggested that methods of oxygen delivery, type of surgery, and risk of bias rating for the RCT are potential effect modifiers. Univariable meta-regression indicated the risk of bias for the RCT being potentially the strongest effect modifier (R^2^=35%). The ratio of RR for high-/unclear risk RCTs *vs* low-risk RCTs was 1.37 (95% CI 1.06–1.79, *P*=0.01), suggesting that RCTs judged to be of low risk of bias tended to show a stronger reduction in the risk of SSI associated with high Fio_2_. In our full meta-regression model including both risk of bias and the method of oxygen delivery which also appeared to be a potential effect modifier based on univariable analysis (R^2^=23%), the effect modification was weakened for both variables and was no longer statistically significant. The findings from meta-regression analyses are summarised in Supplementary material 12.

**Table 3:** Subgroup analyses for surgical site infection (SSI). CI, confidence interval; N_2_O, nitrous oxide.

	No. of RCTs	No. of SSI in the intervention group (%)	No. of SSI in the control group (%)	Relative risk (95% CI), I^2^	Test of subgroup differences (*P*-value)	Univariable meta-regression (*P*-value)	Percent variance explained – R-squared (%) from meta-regression
**Overall**							
All	27	678/6163 (11)	783/6172 (12.7)	0.87 (0.76–1.01) I^2^=42%	NA		NA
**Delivery of oxygen**							
Intubation	22	582/5252 (11)	702/5242 (13)	0.82 (0.71–0.96) I^2^=42%	0.01	0.03	23
No intubation	5	96/911 (11)	81/930 (9)	1.23 (0.93–1.62) I^2^=0%			
**Type of surgery**							
Intra-abdominal including colorectal	16	403/2831 (14)	465/2839 (16)	0.84 (0.69–1.03) I^2^=49%	0.02	0.09	22
C-section	5	96/911 (11)	81/930 (9)	1.23 (0.93–1.62) I^2^=0%			
Other types of surgery	6	179/2421 (7)	237/2403 (10)	0.76 (0.63–0.91) I^2^=0%			
**Urgency of surgery**							
Elective surgery	16	303/2971 (10)	365/2963 (12)	0.85 (0.70–1.04) I^2^=40%	0.06	0.10	9
Emergency surgery	4	39/351 (11)	62/336 (18)	0.60 (0.42–0.86) I^2^=0%			
Mixed	7	336/2841 (12)	356/2873 (12)	1.01 (0.80–1.28) I^2^=52%			
**By risk of bias**							
Low risk	14	426/4106 (10)	551/4146 (13)	0.77 (0.66–0.91) I^2^=29%	0.02	0.01	35
High/unclear risk	13	252/2057 (12)	232/2026 (11)	1.06 (0.85–1.31) I^2^=33%			
**Gas mixture**							
With N_2_O	6	132/1213 (11)	162/1262 (13)	0.94 (0.58–1.51) I^2^=66%	0.73	0.53	0
Without N_2_O	21	546/4950 (11)	621/4910 (13)	0.86 (0.74–0.99) I^2^=33%			

Our analyses excluded a large quasi-randomised trial (*n*=5700) recently published by Kurz and colleagues in 2018,^105^ in which high or low Fio_2_ was alternated every 2 weeks in an operating room. The study was conducted in a pragmatic setting, where 80% or 30% Fio_2_ during major abdominal procedures was allocated to an operation room section at random, and thereafter alternated the Fio_2_ concentration every 2 weeks. The trial design, therefore, differed from most of the other included trials in that the unit of allocation is cluster while the alternating treatment assignment is not usually considered as an adequate method of randomisation. Nevertheless, as the study had a relatively large sample size, could be considered ‘quasi-experimental’, and was included in some previously published systematic reviews, we included the study in a sensitivity analysis to explore whether adding the study to our meta-analysis would have changed our conclusion. Inclusion of data from this trial in a sensitivity analysis did not change our findings from the main analysis (Supplementary material 13).

### GRADE assessment

Overall, the certainty in the evidence was assessed as low. GRADE evidence profile and summary of findings table are presented in Supplementary material 14.

#### Mortality

We selected one anchoring review (Lim and colleagues 2021)^31^ that provided comprehensive evidence on the effectiveness of high *vs* low Fio_2_ on postoperative clinical outcomes in patients undergoing non-thoracic surgery under general anaesthesia. Twenty-six trials with a total of 4991 patients were included in this review. Our updated meta-analyses included 32 trials with 8174 patients, six were newly identified since the publication of the Lim and colleagues review in 2021.^90^^,^^91^^,^^95^^,^^97^^,^^104^

**Fig 6:**
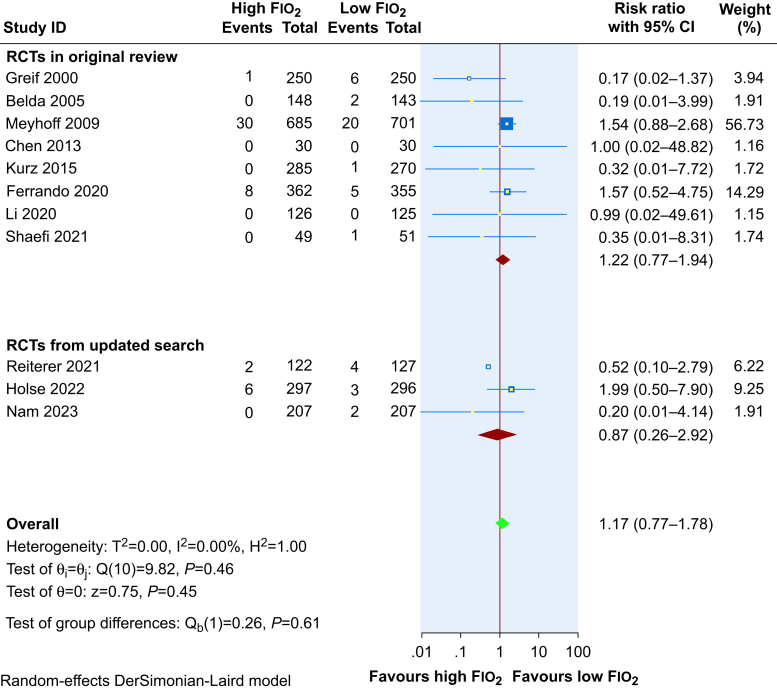
Effect of 80% Fio_2_ on mortality when compared with 30–35% Fio_2_. CI, confidence interval; Fio_2_, fraction of inspired oxygen.

The evidence from 11 studies including 5116 patients suggests that high Fio_2_ does not reduce 30-day mortality (RR 1.17, 95% CI 0.77–1.78, RD 0.3% higher, 95% CI 0.4% lower to 1.3% higher). The forest plot is presented in Fig. 6. GRADE certainty in the evidence was low. There was some evidence of funnel plot asymmetry (Supplementary material 10). Furthermore, regression-based Egger's test for small-study effects was statistically significant (*P*=0.02).

The trial sequential analysis for 30-day mortality showed that the number of patients evaluated did not surpass the required information size (*n*=54 777). The cumulative Z-curve crossed neither the conventional nor trial sequential monitoring boundaries of significance. The result suggests that there is insufficient information to support the conclusion, and further studies are still required.

#### Pulmonary and other outcomes

Updated meta-analyses for pulmonary outcomes including PPCs, pneumonia, respiratory failure, and atelectasis, and for other secondary outcomes including mortality up to longest follow-up, postoperative nausea and vomiting, and length of hospital stay, are presented in Supplementary material 11, and a GRADE summary of evidence table for them can be found in Supplementary material 14. Overall, the certainly of evidence is low to very low and the findings suggest that high Fio_2_ is unlikely to have a major effect on these outcomes, except that high Fio_2_ may result in a large increase in incidence of atelectasis (RR 1.47, 95% CI 1.20–1.79, RD 6.5% increase, 95% CI 2.8%–10.9% increase), based on low-certainty evidence.

### Postoperative noninvasive ventilation *vs* conventional oxygen therapy

The anchoring review (Hui and colleagues,^87^ 2021) included 38 trials with 9782 patients. To avoid the overlap between this review and Chaudhuri and colleagues^59^ review, we focused on trials comparing NIV (including CPAP) with standard postoperative care. We did not identify additional trials from updated search results. SSI was not reported as an outcome in systematic reviews of NIV *vs* COT that we identified.

#### Mortality

**Fig 7:**
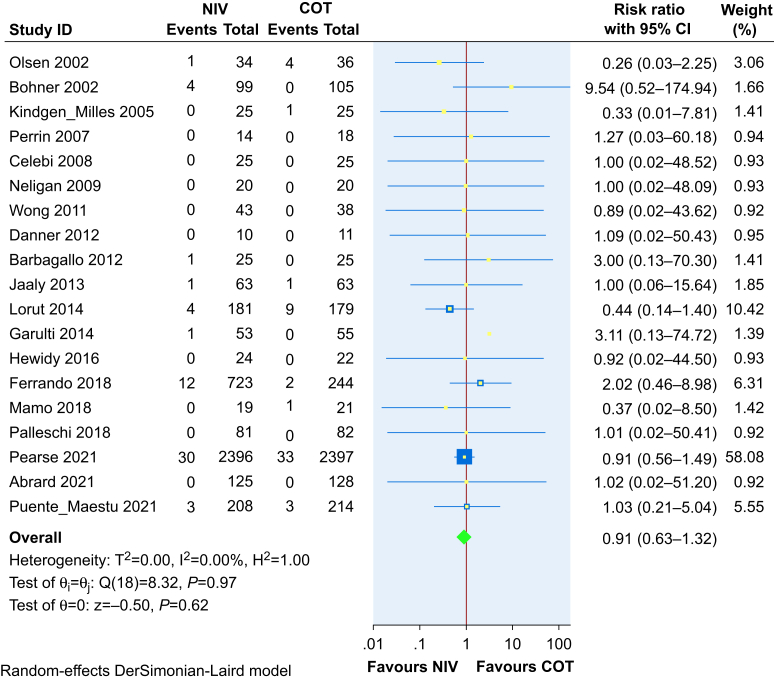
Effect of noninvasive ventilation (NIV) on mortality when compared with conventional oxygen therapy (COT). The required information size was calculated assuming a relative risk reduction of 20% with 90% power and an alpha error of 5%. With a low baseline mortality risk of 1.5%, the required information size would be sufficiently powered to detect a risk difference between intervention and comparison groups of 0.3% (20% × 1.5%), or 3 per 1000. CI, confidence interval.

Nineteen trials, including 7876 patients, reported on mortality (Fig. 7). NIV does not reduce mortality compared with COT in the immediate postoperative period (RR 0.91, 95% CI 0.62–1.32, RD 0.1% reduction, 95% CI 0.6% reduction to 0.5% increase), based on high-certainty evidence. There was no evidence of funnel plot asymmetry (Supplementary material 10) and regression-based Egger's test for small-study effects was not statistically significant (*P*=0.66).

The trial sequential analysis for this outcome showed that the number of patients evaluated did not surpass the required information size (*n*=62 194).

Subgroup analysis based on the type of surgery did not show credible subgroup effects for mortality (Supplementary material 15). The risk of mortality did not differ between patients who underwent abdominal surgery compared with cardiothoracic or mixed surgery. The test of subgroup differences was not statistically significant (*P*=0.36).

#### Pulmonary and other outcomes

Meta-analyses for pulmonary outcomes including PPCs, acute respiratory distress syndrome (ARDS) and pneumonia, and for other secondary outcomes including length of hospital stay, are presented in Supplementary material 15, and a GRADE summary of evidence table can be found in Supplementary material 14. Compared with COT, NIV may decrease the incidence of PPCs based on low-certainty evidence (RR 0.62, 95% CI 0.44–0.87, RD 12.2% reduction, 95% CI 4.2%–18% reduction) and likely results in a slight reduction in the incidence of ARDS (RR 0.70, 95% CI 0.53–0.93, RD 1.2% reduction, 95% CI 1.9%–0.3% reduction, moderate-certainty evidence). NIV probably shortens the length of hospital stay (MD 1.12 days shorter, 95% CI 1.69 to 0.55 days shorter), based on moderate-certainty evidence. An overall summary of key findings for NIV *vs* COT can be found in Figure 3.

### Postoperative high-flow nasal oxygen *vs* conventional oxygen therapy

Our updated meta-analysis included 16 RCTs enrolling 2201 patients, five^92^^,^^98^, ^99^, ^100^, ^101^ were newly identified since the publication of the review by Chaudhuri and colleagues^59^ in 2020.

#### Mortality

**Fig 8:**
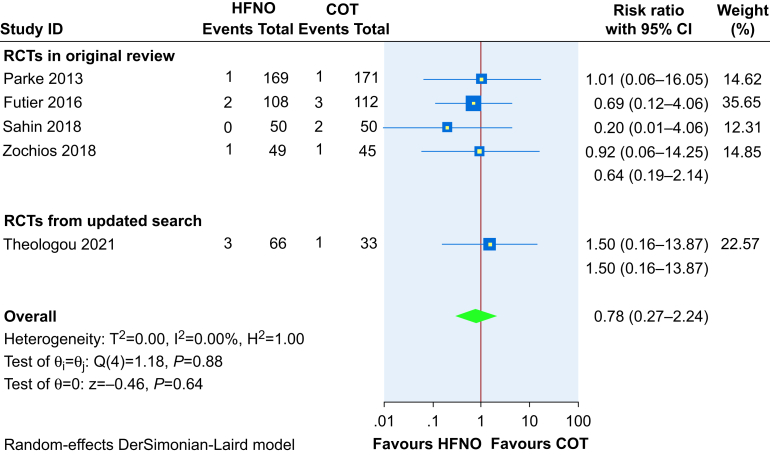
Effect of high-flow nasal oxygen (HFNO) on mortality when compared with conventional oxygen therapy (COT). CI, confidence interval.

Five trials including 853 patients reported on mortality (Fig. 8). HFNO use in the immediate postoperative period does not reduce mortality compared with COT (RR 0.78, 95% CI 0.27–2.24, RD 0.4% reduction, 95% CI 1.4% reduction to 2.4% increase), based on low-certainty evidence.

The trial sequential analysis for this outcome showed that the required information size (*n*=46 433) was not met.

#### Pulmonary and other outcomes

Updated meta-analyses for pulmonary outcomes including risk of reintubation and need for escalation of respiratory support, and for other secondary outcomes including length of hospital stay, are presented in Supplementary material 16, and a GRADE summary of evidence table can be found in Supplementary material 14. HFNO use in the immediate postoperative period may reduce the need for escalation of respiratory support (RR 0.59, 95% CI 0.40–0.88, RD 8% reduction, 95% CI 2.3%–11.7% reduction; very low-certainty evidence). Subgroup analyses based on the type of surgery, risk of postoperative respiratory complications, and body mass index did not show credible subgroup effects (Supplementary material 17). HFNO may shorten the length of hospital stay slightly compared with COT (MD 0.45 days shorter, 95% CI 0.86 days–0.03 days shorter; low-certainty evidence). An overall summary of key findings for HFNO *vs* COT can be found in Figure 3.

### Top-level panoramic meta-analyses

We were unable to perform the top-level panoramic meta-analysis for SSI as this outcome was examined and reported only among trials of high *vs* low Fio_2_ and was not reported in trials comparing NIV or HFNO with COT.

**Fig 9:**
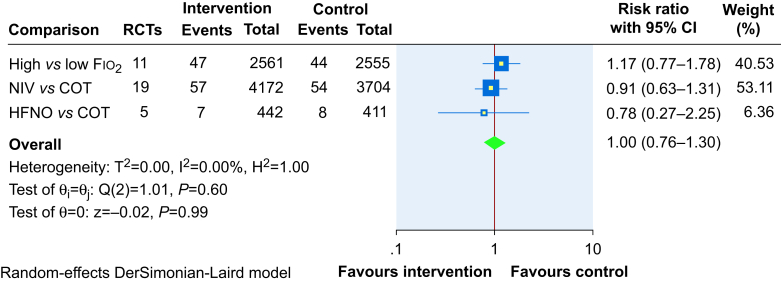
Top-level panoramic meta-analysis on mortality. CI, confidence interval; COT*,* conventional oxygen therapy; Fio_2_, fraction of inspired oxygen; HFNO*,* high*-*flow nasal oxygen; NIV*,* noninvasive ventilation.

Our top-level panoramic meta-analysis across the three main comparisons on mortality (Fig. 9) showed that oxygenation strategies involving delivery of higher concentration of oxygen perioperatively (high Fio_2_/HFNO/NIV) did not influence mortality (RR 1.00, 95% CI 0.76–1.30, RD 0%, 0.4% lower to 0.5% higher), although the total number of deaths included in the analysis was still relatively small.

## Discussion

This is the most compressive review of evidence of the effectiveness of perioperative oxygen therapy strategies. Adopting a panoramic approach to the synthesis of the 59 reviews has revealed several important findings. Firstly, in relation to SSI and mortality, our findings suggest that a high Fio_2_ (80%) is unlikely to have a major effect on SSI compared with low Fio_2_ (30–35%) when administered perioperatively, although a potentially slight reduction in the risk of SSI cannot be ruled out. Intraoperative high Fio_2_ (80%) may result in little to no difference in the risks of 30-day mortality, PPCs, pneumonia, respiratory failure, unplanned ICU admission, and the length of hospital stay, but it may increase the incidence of atelectasis compared with low Fio_2_ (30–35%). Secondly, HFNO and NIV may have little to no effect on mortality compared with COT when administered after surgery. Finally, regarding potential harms and benefits of perioperative oxygen therapy in relation to pulmonary/respiratory outcomes, we found that the evidence is very uncertain.

Although our updated meta-analysis on the effectiveness of 80% *vs* 30–35% Fio_2_ on SSI (RR 0.87, 95% CI 0.76–1.01) rules out a major effect for intraoperative high Fio_2_, a potential benefit of slight reduction in SSI remains a possibility. Our trial sequential analysis showed that 86% of the required information size has been reached so far, consistent with the study by El Maleh and colleagues.^106^ This indicates that further trials are needed to generate conclusive results and recommendations.

Our updated subgroup analyses highlighted a few potential effect modifiers, including methods of oxygen delivery, type of surgery, urgency of surgery, and risk of bias. It is possible that previous trials did not include patients who are at higher risk of complications and more targeted sampling approach is required. However, these analyses were based on trial-level data and therefore may be subject to ecological fallacy. The findings of these subgroup analyses were heavily confounded and required great caution in their interpretation. The level of heterogeneity that remained in various subgroups highlights the need to explore potential effect modifiers potentially using individual-level patient data for within-trial comparisons. Different ventilation strategies were not explored in our subgroup analysis as we expected that there would be too many different approaches to enable an analysis with adequate statistical power. Nevertheless, how ventilation strategies might affect postoperative SSI through their impact on oxygenation may be worth exploring in future research.

Our updated analyses suggest that intraoperative high Fio_2_ does not reduce 30-day mortality and mortality within longest follow-up. These findings are consistent with those documented recently.^44^^,^^51^^,^^107^ The low risk of death for most surgeries and the modest effect that perioperative oxygen therapy might have mean that mortality is unlikely to be a suitable indicator for judging the overall benefit/risk of the intervention. Alternatively, patient-centred outcomes such as days at home after surgery at 30 days have been proposed and may be worth considering.^108^

Despite previous meta-analyses,^39^^,^^44^^,^^107^ our findings suggest that high Fio_2_ may result in a large increase in the incidence of atelectasis (RR 1.47, 95% CI 1.20–1.79; low-certainty evidence). Discrepancy among the studies could be, at least in part, explained by type of inspired gas mixture. N_2_O promotes absorption atelectasis in the lung as effectively as breathing 100% oxygen^109^ and thus its presence in the inhaled gas mixture might have increased the incidence of atelectasis in the low Fio_2_ group, resulting in a comparable incidence of atelectasis between the groups. In a sensitivity analysis including trials with N_2_O in the gas mixture, the effect of high Fio_2_ on the incidence of atelectasis was no longer statistically significant (RR 1.26, 95% CI 0.91–1.73).

In the postoperative setting, our findings suggest that HFNO may reduce the need to escalate respiratory support (RR 0.59, 95% CI 0.40–0.88) compared with COT, but it is worth noting that all trials included in previous and our current meta-analyses were relatively small with few events (<10) in individual treatment arms. The finding therefore remains very uncertain. Our findings also showed potential benefit for postoperative NIV compared with COT in reducing some of the pulmonary adverse events. Nevertheless, the findings were also driven by relatively small trials and were not confirmed by the largest trial to date.^110^

In summary, we found that 80% oxygen administered perioperatively is unlikely to have a major beneficial or harmful effect (i.e. >5 percentage point) on SSI. However, further large-scale trials are needed to confirm or refute a potentially small effect on reducing SSI and the substantial increase in atelectasis compared with COT (30–35% oxygen). This will also mitigate potential bias associated with publication and reporting bias that cannot be ruled out.

### Strengths and limitations of this review

We performed a comprehensive search of published systematic reviews and recent RCTs, registered our overview methods prospectively, and followed through our protocol.

The strength of our overview stems from it being the most comprehensive synthesis of evidence that combines all relevant reviews on the topic. It signposts clinicians and policymakers toward relevant systematic reviews to support clinical decision-making. There is a relatively large number of overlapping systematic reviews which makes interpretation of the evidence difficult. Our overview will play a key role in research prioritisation, ensuring effective use of resources and avoiding duplication.

Reducing SSI has been the target of numerous clinical and quality improvement interventions, and as a result its incidence has been decreasing over time.^111^ This raises a question of applicability of evidence for some of the trials conducted more than a decade ago. Nevertheless, we did not notice any clear trend of the effect of perioperative oxygen therapy having changed over time. Current evidence is dominated by studies conducted in developed countries; insights may be gained from studies conducted in developing countries where the incidence of SSI is much higher and represents a much more serious problem. We were unable to include any evidence of cost and resource implications of perioperative oxygen therapy as this was not considered in studies conducted in developed countries. In addition, we did not find any systematic reviews in paediatric population.

Evidence included in our analyses was (to some extent) still bounded by our chosen anchoring reviews as we did not go back to original RCT reports unless there were data queries or a reassessment of risk of bias was necessary. In addition, we did not have access to individual patient data which would allow us to explore potential influence of patient characteristics such as age and underlying disease condition on the effect of perioperative oxygen therapy and other patient-focused outcomes of interest.

We conducted trial sequential analysis to control random errors as a result of repeated significance testing when meta-analyses are updated, and to assess whether further trials are needed. We recognise that trial sequential analysis has been criticised for being too conservative, as sceptical *a priori* assumed intervention effects can cause the required information size to become unreasonably large.^22^ This became apparent in some of the analyses that we conducted: where the baseline (control group) risk of an event of interest is low, a pre-specified intervention effect in relative terms (e.g. RRR of 20%) could result in the calculation of an unrealistically large optimal information size that is aiming to detect a trivial intervention effect in absolute terms.

### Implications for practice

Although the WHO recommends the use of a high concentration of intraoperative oxygen (80%) as a strategy to reduce SSIs, we did not find evidence to support routine use of high inspired oxygen to reduce SSIs. Given the knowledge of potentially harmful effects of high level of oxygen in cardiac and critical care settings,^35^^,^^112^ it is likely that the benefits/harms of perioperative oxygen do not increase/decrease in a linear fashion, and an optimal concentration of oxygen may need to be found and calibrated depending on the patient's condition and type of surgery. An intraoperative Fio_2_ of 50% currently represents standard intraoperative practice in the UK.^4^ This seems to be a rational practice considering the unknown trade-off between potential harms and benefits. Nevertheless, this differs from both WHO's recommendation of using an Fio_2_ of 80% intraoperatively, and the value most previous interventional oxygen therapy trials have used to represent standard care (typically 30% Fio_2_).

### Implications for research

Our review has identified six gaps in the evidence base that need to be explored to determine the most clinically effective perioperative oxygen therapy strategies and important effect modifiers: (1) large-scale RCTs are needed on the clinical and cost effectiveness of different amounts of oxygen on SSI, PPCs, and patient-centred outcomes that encompass both potential benefits and harms; (2) large-scale pragmatic RCTs of postoperative NIV and HFNO to confirm or refute their clinical and cost effectiveness in reducing PPCs; (3) head-to-head RCTs to establish effectiveness of postoperative HFNO compared with NIV, especially in patients at higher risk for PPCs; (4) high-quality systematic reviews to establish the benefits of perioperative oxygen therapy in paediatric populations; (5) living systematic reviews/overviews to reduce research waste and replace the need for multiple systematic reviews with overlapping efforts and potentially inconsistent conclusions; and (6) pre-specified and theory-driven analyses based on data from large-scale RCTs or collaborative individual patient data meta-analyses to explore and identify potential effect modifiers of perioperative oxygen therapy.

### Conclusions

Use of high Fio_2_ (80%) is unlikely to have a large beneficial or a harmful effect on the incidence of surgical site infection and mortality (RD of ≥5 percentage point) compared with low Fio_2_ (30%). However, there is a potentially large effect of increased atelectasis. Current evidence is insufficient to confirm or rule out a moderate to small effect of high Fio_2_ *vs* low Fio_2_ for various outcomes. Therefore, evidence is lacking to support the recommendations or practice trends towards increased use of high Fio_2_.

When considering alternative perioperative oxygen therapy strategies, postoperative noninvasive ventilation has little or no effect on mortality compared with conventional oxygen therapy. Noninvasive ventilation might reduce other adverse respiratory events, but the evidence is insufficient to quantify the magnitude of the benefits or to confirm the applicability of the findings in routine use. Evidence is also insufficient to confirm or rule out a potentially large beneficial effect or a moderate harmful effect on various respiratory outcomes for postoperative high-flow nasal oxygen compared with conventional oxygen therapy. Trial sequential analysis indicated that more trials are needed to reduce the uncertainty in the field.

## Authors’ contributions

Contributed to title/abstract screening and full-text screening: ST, AW, AH

Led the overview: AE

Involved in the design of the overview: YFC, AG, KC, OU, JY

Oversaw its conduct: YFC, AG, KC, JY

Data extraction: ST, AW, AH, AB, SV

Mapping of studies: ST, AW, AH, AB, SV

Helped reviewing mapping of studies: MT

Synthesis strategy and interpretation of evidence: MT

Led the database search: RC

Designed and ran the searches for the systematic overview: RC

Managed the references and referencing: RC

Provided clinical guidance: KC, JY

Maintained day-to-day running of the project: AE

Involved in all stages of the review process: AE

Quality assessment: ST

Drafted the manuscript: AE, YFC, AG, KC, ST, JY

Revised the manuscript: AE, YFC, AG, KC, OU, JY

Principal investigator and grant holder: JY

Oversaw the public and patient engagement sessions: JY

The corresponding author attests that all listed authors meet authorship criteria and that no others meeting the criteria have been omitted.

## Provenance and peer review

This manuscript presents independent research commissioned by the National Institute for Health and Care Research (NIHR). The views and opinions expressed by the authors in this publication are those of the authors and do not necessarily reflect those of the NHS, the NIHR, the Health Technology Assessment Programme, or the Department of Health.

## Funding

10.13039/501100000272National Institute for Health and Care Research (NIHR) Health Technology Assessment (HTA) Programme (132987 to JY); NIHR Evidence Synthesis Programme (14/25/05 to AG, YFC, and RC); NIHR Advanced Fellowship (NIHR300060 to AG and RC); NIHR Applied Research Collaboration (ARC) West Midlands (NIHR200165 to AG); NIHR Academic Clinical Fellowship (ACF-2021-08-003 to ST).

## Declarations of interest

All authors have completed the ICMJE uniform disclosure form and declare: YFC is a member of the NIHR Evidence Synthesis Programme Prioritisation and Advisory Group. AG is a member of the Member of the NIHR HTA Commissioning Committee and a member of the NIHR DSE Fellowship Funding Committee. KC is a member of NIHR HTA hospital-based prioritisation committee and NIHR RfPB West Midlands funding committee. JY is a member of NIHR HTA General Committee. Otherwise no other authors declare any competing interests. The lead author (AE) affirms that the manuscript is an honest, accurate, and transparent account of the study being reported; that no important aspects of the study have been omitted; and that any discrepancies from the study as planned have been explained.
