# Supplementary material for: Perioperative oxygen therapy: an overview of systematic reviews and meta-analyses
Source: Br J Anaesth. 2025 Jun 6;135(5):1456–76. doi: 10.1016/j.bja.2025.04.020 (PMC12597348; doi:10.1016/j.bja.2025.04.020)
Supplement: Supplementary material 1 [file mmc1.docx]

**Supplementary file 1: detailed search strategies for the review**

**Search for Systematic Reviews**

**Bibliographic databases: search summary**

| **Source(s) and date coverage** | **Date searched** | **Purpose** | **Description of search** | **Hits** | **Notes** |
| --- | --- | --- | --- | --- | --- |
| Epistemonikos | 14/09/2021 | Search for systematic reviews | Surgery  AND oxygen/relevant interventions | 769 | Specialist database for systematic reviews and overviews. Filtered for publication types:   - Systematic Review - Broad Synthesis - No date or language limits applied. |
| MEDLINE  (Ovid) | 14/09/2021 | Search for systematic reviews | Surgery  AND oxygen/relevant interventions  AND  systematic review search filter | 1396 | No date or language limits applied. |
| Embase  (Ovid) | 14/09/2021 | Search for systematic reviews | As for MEDLINE above | 2863 | No date or language limits applied. |
| Cochrane Database of Systematic Reviews (Wiley) | 15/09/2021 | Search for systematic reviews | As for Epistemonikos above | 223 | - Specialist database for Cochrane systematic reviews and protocols. - No date or language limits applied. |
| PROSPERO (CRD) | 15/09/2021 | Search for systematic reviews | As for Epistemonikos above | 325 | - Specialist database for systematic review protocols. - No date or language limits applied |
| HTA Database (INAHTA) | 15/09/2021 | Search for systematic reviews | As for Epistemonikos above | 93 | Specialist database for health technology assessments.  Not filtered by publication type because many records are currently unassigned.  No date or language limits applied. |
| DARE archives (CRD) | 16/09/2021 | Search for systematic reviews | As for Epistemonikos above | 218 | Specialist database for systematic reviews.  Discontinued - records stopped being added in 2015.  No date or language limits applied. |

**Totals for PRISMA flow diagram**

Total from bibliographic databases: 5887

Total after deduplication in EndNote: 4067

Update searches: June 2022

For details of update searches, see below each source search strategy.

Notes: Not for inclusion in flow diagram. For scanning by reviewers for awareness and potential mention in discussion, etc.

**Bibliographic databases: searches**

**Epistemonikos**

https://www.epistemonikos.org/

Search date: 14/09/2021

Advanced Search

(title:(surg* OR operat* OR preoperativ* OR intraoperativ* OR perioperativ* OR postoperative* OR pre-operativ* OR intra-operativ* OR peri-operativ* OR post-operative* OR presurg* OR intrasurg* OR perisurg* OR postsurg* OR pre-surg* OR intra-surg* OR peri-surg* OR post-surg*) OR abstract:(surg* OR operat* OR preoperativ* OR intraoperativ* OR perioperativ* OR postoperative* OR pre-operativ* OR intra-operativ* OR peri-operativ* OR post-operative* OR presurg* OR intrasurg* OR perisurg* OR postsurg* OR pre-surg* OR intra-surg* OR peri-surg* OR post-surg*)) AND (title:(oxygen* OR hyperoxia OR "non-invasive ventilation" OR "non invasive ventilation" OR "noninvasive ventilation" OR "nasal cannula" OR "nasal cannulae" OR "nasal cannulas" OR "high flow" OR highflow OR high-flow OR HFNO OR HFOC OR "continuous positive airway pressure" OR CPAP OR "continuous positive pressure ventilation" OR CPPV OR "bi level positive airway pressure" OR "bilevel positive airway pressure" OR "bi-level positive airway pressure" OR BiPaP) OR abstract:(oxygen* OR "non-invasive ventilation" OR "non invasive ventilation" OR "noninvasive ventilation" OR "nasal cannula" OR "nasal cannulae" OR "nasal cannulas" OR "high flow" OR highflow OR high-flow OR HFNO OR HFOC OR "continuous positive airway pressure" OR CPAP OR "continuous positive pressure ventilation" OR CPPV OR "bi level positive airway pressure" OR "bilevel positive airway pressure" OR "bi-level positive airway pressure" OR BiPaP))

Filters applied:

Systematic Review: 725

Broad Synthesis: 44

Update 30/06/2022

Re-ran above search, with following data limit

Added to database: Custom date range: From: 14-09-21 To: 30-06-22

Systematic Review: 105

Broad Synthesis: 2

Alerts: Not possible to set up alerts

**MEDLINE (Ovid)**

Search date: 14/09/2021

Exact database segment searched: Ovid MEDLINE(R) ALL <1946 to September 13, 2021>

Search Strategy:

--------------------------------------------------------------------------------

1 exp Specialties, Surgical/ (207628)

2 exp Surgical Procedures, Operative/ (3311503)

3 (surger* or surgical or surgeon* or operat* or preoperativ* or intraoperativ* or perioperativ* or postoperative* or pre-operativ* or intra-operativ* or peri-operativ* or post-operative* or presurg* or intrasurg* or perisurg* or postsurg* or pre-surg* or intra-surg* or peri-surg* or post-surg*).ti,ab,kf. (3094965)

4 1 or 2 or 3 (5123023)

5 Oxygen/ (169396)

6 exp Oxygen Inhalation Therapy/ (27158)

7 Hyperoxia/ (3989)

8 Noninvasive ventilation/ (2810)

9 Positive-Pressure Respiration/ (17710)

10 Continuous Positive Airway Pressure/ (8010)

11 (oxygen* or non invasive ventilation or noninvasive ventilation or nasal cannula* or high flow or highflow or HFNO or HFOC or continuous positive airway pressure or CPAP or continuous positive pressure ventilation or CPPV or bi level positive airway pressure or bilevel positive airway pressure or BiPaP).ti,ab,kf. (625166)

12 5 or 6 or 7 or 8 or 9 or 10 or 11 (710401)

13 4 and 12 (102058)

14 (metaanalys* or meta analys* or meta-analys* or NMA* or MAIC* or indirect comparison* or indirect treatment comparison* or mixed treatment comparison*).mp. (247161)

15 ((systematic* adj3 (review* or overview* or search or literature)) or umbrella review*).mp. (271732)

16 (technology assessment* or HTA or HTAs or technology overview* or technology appraisal*).mp. (17024)

17 14 or 15 or 16 (406117)

18 13 and 17 (1396)

19 limit 13 to (meta analysis or "systematic review") (951)

20 18 or 19 (1396)

Update 30/06/2022

Re-ran above search, with following data limit:

21 (20210915 or 20210916 or 20210917 or 20210918 or 20210919 or 2021092* or 202110* or 202111* or 202112* or 2022*).dt,ez,da. (1713558)

22 20 and 21 (242)

30/06/2022 Alert set up - emailing to RC

**Embase (Ovid)**

Search date: 14/09/2021

Database: Embase Classic+Embase <1947 to 2021 Week 36>

Search Strategy:

--------------------------------------------------------------------------------

1 exp surgery/ (5639002)

2 exp surgeon/ (177960)

3 perioperative medicine/ (289)

4 (surger* or surgical or surgeon* or operat* or preoperativ* or intraoperativ* or perioperativ* or postoperative* or pre-operativ* or intra-operativ* or peri-operativ* or post-operative* or presurg* or intrasurg* or perisurg* or postsurg* or pre-surg* or intra-surg* or peri-surg* or post-surg*).ti,ab,kw. (4262932)

5 1 or 2 or 3 or 4 (7197759)

6 oxygen/ (256042)

7 oxygen therapy/ (37316)

8 hyperoxia/ (10603)

9 exp noninvasive ventilation/ (15524)

10 exp positive pressure ventilation/ (10805)

11 (oxygen* or non invasive ventilation or noninvasive ventilation or nasal cannula* or high flow or highflow or HFNO or HFOC or continuous positive airway pressure or CPAP or continuous positive pressure ventilation or CPPV or bi level positive airway pressure or bilevel positive airway pressure or BiPaP).ti,ab,kw. (797248)

12 6 or 7 or 8 or 9 or 10 or 11 (907180)

13 5 and 12 (165461)

14 (metaanalys* or meta analys* or meta-analys* or NMA* or MAIC* or indirect comparison* or indirect treatment comparison* or mixed treatment comparison*).mp. (357848)

15 ((systematic* adj3 (review* or overview* or search or literature)) or umbrella review*).mp. (432167)

16 (technology assessment* or HTA or HTAs or technology overview* or technology appraisal*).mp. (26895)

17 14 or 15 or 16 (617074)

18 13 and 17 (2863)

Update 30/06/2022

Re-ran above search, with following data limit:

limit 18 to dc=20210914-20220630 (499)

30/06/2022 Alert set up - emailing to RC

**Cochrane Database of Systematic Reviews (Wiley)**

Search date: 15/09/2021

ID Search Hits

#1 MeSH descriptor: [Specialties, Surgical] explode all trees 1942

#2 MeSH descriptor: [Surgical Procedures, Operative] explode all trees 123492

#3 (surg* or operat* or preoperativ* or intraoperativ* or perioperativ* or postoperative* or pre-operativ* or intra-operativ* or peri-operativ* or post-operative* or presurg* or intrasurg* or perisurg* or postsurg* or pre-surg* or intra-surg* or peri-surg* or post-surg*):ti,ab,kw 321509

#4 #1 or #2 or #3 356935

#5 MeSH descriptor: [Oxygen] this term only 5257

#6 MeSH descriptor: [Oxygen Inhalation Therapy] explode all trees 1650

#7 MeSH descriptor: [Hyperoxia] this term only 211

#8 MeSH descriptor: [Noninvasive Ventilation] this term only 295

#9 MeSH descriptor: [Positive-Pressure Respiration] this term only 1564

#10 MeSH descriptor: [Continuous Positive Airway Pressure] this term only 1173

#11 (oxygen* or "non invasive ventilation" or "noninvasive ventilation" or (nasal next cannula*) or "high flow" or highflow or HFNO or HFOC or "continuous positive airway pressure" or CPAP or "continuous positive pressure ventilation" or CPPV or "bi level positive airway pressure" or "bilevel positive airway pressure" or BiPaP):ti,ab,kw 59119

#12 #5 or #6 or #7 or #8 or #9 or #10 or #11 59619

#13 #4 and #12 17322

Cochrane Reviews: 220

Cochrane Protocols: 3

Update 30/06/2022.

Re-ran above search, with following data limit:

#14 #4 and #12 with Cochrane Library publication date from Sep 2021 to Jun 2022 951

Cochrane Reviews: 9

Cochrane Protocols: 0

30/06/2022 Alert set up - emailing to RC

**PROSPERO**

https://www.crd.york.ac.uk/prospero/

Search date: 15/09/2021

#1 (surg* OR operat* OR preoperativ* OR intraoperativ* OR perioperativ* OR postoperative* OR pre-operativ* OR intra-operativ* OR peri-operativ* OR post-operative* OR presurg* OR intrasurg* OR perisurg* OR postsurg* OR pre-surg* OR intra-surg* OR peri-surg* OR post-surg*):TI,CS,HA,KW,PA,RQ 24143

#2 (oxygen* OR non-invasive ventilation OR non invasive ventilation OR noninvasive ventilation OR nasal cannula OR nasal cannulas OR high flow OR highflow OR high-flow OR HFNO OR HFOC OR continuous positive airway pressure OR CPAP OR continuous positive pressure ventilation OR CPPV OR bi level positive airway pressure OR bilevel positive airway pressure OR bi-level positive airway pressure OR BiPaP):TI,KW,RQ 1031

#3 (oxygen* OR non-invasive ventilation OR non invasive ventilation OR noninvasive ventilation OR nasal cannula OR nasal cannulas OR high flow OR highflow OR high-flow OR HFNO OR HFOC OR continuous positive airway pressure OR CPAP OR continuous positive pressure ventilation OR CPPV OR bi level positive airway pressure OR bilevel positive airway pressure OR bi-level positive airway pressure OR BiPaP):IV 1011

#4 #2 OR #3 1484

#5 #1 AND #4 325

**HTA Database (INAHTA)**

https://database.inahta.org/

Search date: 15/09/2021

(((oxygen* or "non invasive ventilation" or "non-invasive ventilation" or "noninvasive ventilation" or "nasal cannula" or "nasal cannulas" or "nasal cannulae" or "high flow" or "high-flow"or highflow or HFNO or HFOC or "continuous positive airway pressure" or CPAP or "continuous positive pressure ventilation" or CPPV or "bi level positive airway pressure" or "bi-level positive airway pressure" or "bilevel positive airway pressure" or BiPaP)[abs]) OR ((oxygen* or "non invasive ventilation" or "non-invasive ventilation" or "noninvasive ventilation" or "nasal cannula" or "nasal cannulas" or "nasal cannulae" or "high flow" or "high-flow"or highflow or HFNO or HFOC or "continuous positive airway pressure" or CPAP or "continuous positive pressure ventilation" or CPPV or "bi level positive airway pressure" or "bi-level positive airway pressure" or "bilevel positive airway pressure" or BiPaP)[title]) OR ("Continuous Positive Airway Pressure"[mh]) OR ("Positive-Pressure Respiration"[mh]) OR ("Noninvasive Ventilation"[mh]) OR ("Hyperoxia"[mh]) OR ("Oxygen Inhalation Therapy"[mhe]) OR ("Oxygen"[mh])) AND ((((surger* or surgical or surgeon* or operat* or preoperativ* or intraoperativ* or perioperativ* or postoperativ* or pre-operativ* or intra-operativ* or peri-operativ* or post-operative* or "pre operative" or "intra operative" or "peri operative" or "post operative" or presurg* or intrasurg* or perisurg* or postsurg* or pre-surg* or intra-surg* or peri-surg* or post-surg*)[abs]) OR ((surger* or surgical or surgeon* or operat* or preoperativ* or intraoperativ* or perioperativ* or postoperativ* or pre-operativ* or intra-operativ* or peri-operativ* or post-operative* or "pre operative" or "intra operative" or "peri operative" or "post operative" or presurg* or intrasurg* or perisurg* or postsurg* or pre-surg* or intra-surg* or peri-surg* or post-surg*)[title])) OR ("Surgical Procedures, Operative"[mhe]) OR ("Specialties, Surgical"[mhe]))

93

Update 30/06/2022

Re-ran above search

97

Sorted by publication year and exported records from 2021 and 2022: 4

**DARE archives (CRD)**

Search date: 15/09/2021

| 1 | MeSH DESCRIPTOR Specialties, Surgical EXPLODE ALL TREES | 375 |
| --- | --- | --- |
| 2 | MeSH DESCRIPTOR Surgical Procedures, Operative EXPLODE ALL TREES | 16740 |
| 3 | (surg* OR operat* OR preoperativ* OR intraoperativ* OR perioperativ* OR postoperative* OR pre-operativ* OR intra-operativ* OR peri-operativ* OR post-operative* OR presurg* OR intrasurg* OR perisurg* OR postsurg* OR pre-surg* OR intra-surg* OR peri-surg* OR post-surg*) | 19957 |
| 4 | #1 OR #2 OR #3 | 25084 |
| 5 | MeSH DESCRIPTOR Oxygen | 105 |
| 6 | MeSH DESCRIPTOR Oxygen Inhalation Therapy EXPLODE ALL TREES | 172 |
| 7 | MeSH DESCRIPTOR Hyperoxia | 3 |
| 8 | MeSH DESCRIPTOR Noninvasive Ventilation | 17 |
| 9 | MeSH DESCRIPTOR Positive-Pressure Respiration | 97 |
| 10 | MeSH DESCRIPTOR Continuous Positive Airway Pressure | 122 |
| 11 | (oxygen* or non invasive ventilation or noninvasive ventilation or nasal cannula* or high flow or highflow or HFNO or HFOC or continuous positive airway pressure or CPAP or continuous positive pressure ventilation or CPPV or bi level positive airway pressure or bilevel positive airway pressure or BiPaP) | 1340 |
| 12 | #5 OR #6 OR #7 OR #8 OR #9 OR #10 OR #11 | 1384 |
| 13 | #4 AND #12 | 392 |
| 14 | (#4 AND #12) IN DARE | 218 |

Not updated because no new records added since previous search.

**Sources of guidelines**

Considered ECRI, GIN and NICE Evidence.

ECRI Guidelines Trust

<https://guidelines.ecri.org/> (free registration required)

Search date: 02/12/2021

((oxygen* OR "non invasive ventilation" OR "noninvasive ventilation" OR "nasal cannula" OR "nasal cannulae" OR "nasal cannulas" OR "high flow" OR highflow OR HFNO OR HFOC OR "continuous positive airway pressure" OR CPAP OR "continuous positive pressure ventilation" OR CPPV OR "bi level positive airway pressure" OR "bilevel positive airway pressure" OR BiPaP) AND (surg* OR operat* OR preoperativ* OR intraoperativ* OR perioperativ* OR postoperative* OR pre-operativ* OR intra-operativ* OR peri-operativ* OR post-operative* OR presurg* OR intrasurg* OR perisurg* OR postsurg* OR pre-surg* OR intra-surg* OR peri-surg* OR post-surg*))

47

Information Specialist filtered and sent 9 potentially relevant records to reviewers for the final check.

NICE Evidence Search

<https://www.evidence.nhs.uk/>

Search date: 03/12/2021

((oxygen* OR "non invasive ventilation" OR "noninvasive ventilation" OR "nasal cannula" OR "nasal cannulae" OR "nasal cannulas" OR "high flow" OR highflow OR HFNO OR HFOC OR "continuous positive airway pressure" OR CPAP OR "continuous positive pressure ventilation" OR CPPV OR "bi level positive airway pressure" OR "bilevel positive airway pressure" OR BiPaP) AND (surg* OR operat* OR preoperativ* OR intraoperativ* OR perioperativ* OR postoperative* OR "pre operative" OR "intra operative" OR "peri operative" OR "post operative" OR presurg* OR intrasurg* OR perisurg* OR postsurg* OR "pre surgery" OR "intra surgery" OR "peri surgery" OR "post surgery" OR "pre surgical" OR "intra surgical" OR "peri surgical" OR "post surgical"))

Sorted by: Relevance

Filtered by: Evidence type: Guidance

950 (looked through first 50)

Information Specialist filtered first 50 for records not picked up by ECRI search.

**Search for recent RCTs**

**Bibliographic databases: search strategies**

**Bibliographic databases: search summary**

| **Source(s) and date coverage** | **Date searched** | **Purpose** | **Description of search** | **Hits** | **Notes** |
| --- | --- | --- | --- | --- | --- |
| CENTRAL (Cochrane Library via Wiley) | 11/03/2022 | Search for recent RCTs | Surgery (specific terms)  AND oxygen/relevant interventions | 3361 | Limited by date added to database. Older trial register records (added to trial registers before 2018) removed. |

Update searches: June 2022

For details of update searches, see below source search strategy.

Notes: Not for inclusion in flow diagram. For scanning by reviewers for awareness and potential mention in discussion, etc.

**CENTRAL (Cochrane Library)**

Search date: 11/03/2022

ID Search Hits

#1 MeSH descriptor: [Specialties, Surgical] explode all trees 2009

#2 MeSH descriptor: [Surgical Procedures, Operative] explode all trees 127199

#3 (preoperativ* or intraoperativ* or perioperativ* or postoperative* or pre-operativ* or intra-operativ* or peri-operativ* or post-operative* or presurg* or intrasurg* or perisurg* or postsurg* or pre-surg* or intra-surg* or peri-surg* or post-surg*):ti,ab,kw 172873

#4 #1 or #2 or #3 240317

#5 MeSH descriptor: [Oxygen] this term only 5393

#6 MeSH descriptor: [Oxygen Inhalation Therapy] explode all trees 1712

#7 MeSH descriptor: [Hyperoxia] this term only 221

#8 MeSH descriptor: [Noninvasive Ventilation] this term only 327

#9 MeSH descriptor: [Positive-Pressure Respiration] this term only 1602

#10 MeSH descriptor: [Continuous Positive Airway Pressure] this term only 1249

#11 (oxygen* or "non invasive ventilation" or "noninvasive ventilation" or "non invasive pressure support" or "noninvasive pressure support" or "intermittent positive-pressure breathing" or "intermittent positive pressure breathing" or "intermittent positive-pressure ventilation" OR "intermittent positive pressure ventilation" or (nasal next cannula*) or "high flow" or highflow or HFNO or HFOC or "continuous positive airway pressure" or CPAP or "continuous positive pressure ventilation" or CPPV or "bi level positive airway pressure" or "bilevel positive airway pressure" or BiPaP):ti,ab,kw 61984

#12 #5 or #6 or #7 or #8 or #9 or #10 or #11 62465

#13 #4 and #12 with Cochrane Library publication date from Jan 2018 to present, in Trials 4631

Total in CENTRAL: 4631

Total after older trial register records (added to trial registers before 2018) removed: 3361

Total after duplicates in CENTRAL removed: 3304

Total after records of RCTs already identified through review of reviews removed: 3279

Update 30/06/2022

Re-ran above search, with following data limit:

#14 #13 with Cochrane Library publication date from Mar 2022 to Jun 2022, in Trials 224

30/06/2022 Alert set up - emailing to RC
