# Supplementary material for: Perioperative oxygen therapy: an overview of systematic reviews and meta-analyses
Source: Br J Anaesth. 2025 Jun 6;135(5):1456–76. doi: 10.1016/j.bja.2025.04.020 (PMC12597348; doi:10.1016/j.bja.2025.04.020)
Supplement: Supplementary material 2 [file mmc2.docx]

**Supplementary file 2: overlap of primary studies in the included reviews.**

***Overlap of primary studies in reviews comparing high vs low FiO_2_***

| Study | de Jonge 2019 | Cohen 2018 | Chu 2018 (IOTA) | Zhang 2016 | Wetterslev 2015 | Brar 2011 | Al-Niaimi 2008 | Fasquel 2020 | Hovaguimian 2013 | Kao 2012 | Klingel 2013 | Koo 2019 | Lim 2021 | Brar 2011 | Dahlke 2013 | Martin 2015 | Mejia 2007 | Patel 2013 | Qadan 2009 | Rincon-valensuela 2012 | Mattishent 2019 | Orhan-sungur 2008 | Shaffer 2021 | Smith 2020 | Togioka 2012 | Wang 2017 | yang 2016 | Zhao 2016 | Xiao 2019 |
| --- | --- | --- | --- | --- | --- | --- | --- | --- | --- | --- | --- | --- | --- | --- | --- | --- | --- | --- | --- | --- | --- | --- | --- | --- | --- | --- | --- | --- | --- |
| Admadé 2013 |  |  |  |  |  |  |  |  |  |  |  |  |  |  |  |  |  |  |  |  |  |  |  |  |  |  |  |  |  |
| Alvandipour 2019 |  |  |  |  |  |  |  |  |  |  |  |  |  |  |  |  |  |  |  |  |  |  |  |  |  |  |  |  |  |
| Akca 1999 |  |  |  |  |  |  |  |  |  |  |  |  |  |  |  |  |  |  |  |  |  |  |  |  |  |  |  |  |  |
| Belda 2005 |  |  |  |  |  |  |  |  |  |  |  |  |  |  |  |  |  |  |  |  |  |  |  |  |  |  |  |  |  |
| Benoît 2002 |  |  |  |  |  |  |  |  |  |  |  |  |  |  |  |  |  |  |  |  |  |  |  |  |  |  |  |  |  |
| Bhatnagar 2005 |  |  |  |  |  |  |  |  |  |  |  |  |  |  |  |  |  |  |  |  |  |  |  |  |  |  |  |  |  |
| Bickel 2011 |  |  |  |  |  |  |  |  |  |  |  |  |  |  |  |  |  |  |  |  |  |  |  |  |  |  |  |  |  |
| Chen 2013 |  |  |  |  |  |  |  |  |  |  |  |  |  |  |  |  |  |  |  |  |  |  |  |  |  |  |  |  |  |
| Duggal 2013 |  |  |  |  |  |  |  |  |  |  |  |  |  |  |  |  |  |  |  |  |  |  |  |  |  |  |  |  |  |
| Edmark 2014 |  |  |  |  |  |  |  |  |  |  |  |  |  |  |  |  |  |  |  |  |  |  |  |  |  |  |  |  |  |
| Eskandr 2019 |  |  |  |  |  |  |  |  |  |  |  |  |  |  |  |  |  |  |  |  |  |  |  |  |  |  |  |  |  |
| Fariba 2016 |  |  |  |  |  |  |  |  |  |  |  |  |  |  |  |  |  |  |  |  |  |  |  |  |  |  |  |  |  |
| Ferrando 2020 |  |  |  |  |  |  |  |  |  |  |  |  |  |  |  |  |  |  |  |  |  |  |  |  |  |  |  |  |  |
| Gardella 2008 |  |  |  |  |  |  |  |  |  |  |  |  |  |  |  |  |  |  |  |  |  |  |  |  |  |  |  |  |  |
| Golfam 2011 |  |  |  |  |  |  |  |  |  |  |  |  |  |  |  |  |  |  |  |  |  |  |  |  |  |  |  |  |  |
| Goll 2001 |  |  |  |  |  |  |  |  |  |  |  |  |  |  |  |  |  |  |  |  |  |  |  |  |  |  |  |  |  |
| Greif 1999 |  |  |  |  |  |  |  |  |  |  |  |  |  |  |  |  |  |  |  |  |  |  |  |  |  |  |  |  |  |
| Greif 2000 |  |  |  |  |  |  |  |  |  |  |  |  |  |  |  |  |  |  |  |  |  |  |  |  |  |  |  |  |  |
| Habib 2015 |  |  |  |  |  |  |  |  |  |  |  |  |  |  |  |  |  |  |  |  |  |  |  |  |  |  |  |  |  |
| Joris 2003 |  |  |  |  |  |  |  |  |  |  |  |  |  |  |  |  |  |  |  |  |  |  |  |  |  |  |  |  |  |
| Kotani 2000 |  |  |  |  |  |  |  |  |  |  |  |  |  |  |  |  |  |  |  |  |  |  |  |  |  |  |  |  |  |
| Kim 2020 |  |  |  |  |  |  |  |  |  |  |  |  |  |  |  |  |  |  |  |  |  |  |  |  |  |  |  |  |  |
| Kurz 2015 |  |  |  |  |  |  |  |  |  |  |  |  |  |  |  |  |  |  |  |  |  |  |  |  |  |  |  |  |  |
| Li 2020 |  |  |  |  |  |  |  |  |  |  |  |  |  |  |  |  |  |  |  |  |  |  |  |  |  |  |  |  |  |
| Mayank 2019 |  |  |  |  |  |  |  |  |  |  |  |  |  |  |  |  |  |  |  |  |  |  |  |  |  |  |  |  |  |
| Mayzler 2005 |  |  |  |  |  |  |  |  |  |  |  |  |  |  |  |  |  |  |  |  |  |  |  |  |  |  |  |  |  |
| McKeen 2009 |  |  |  |  |  |  |  |  |  |  |  |  |  |  |  |  |  |  |  |  |  |  |  |  |  |  |  |  |  |
| Meyhoff 2009 |  |  |  |  |  |  |  |  |  |  |  |  |  |  |  |  |  |  |  |  |  |  |  |  |  |  |  |  |  |
| Myles 2007 |  |  |  |  |  |  |  |  |  |  |  |  |  |  |  |  |  |  |  |  |  |  |  |  |  |  |  |  |  |
| Park 2020 |  |  |  |  |  |  |  |  |  |  |  |  |  |  |  |  |  |  |  |  |  |  |  |  |  |  |  |  |  |
| Pryor 2004 |  |  |  |  |  |  |  |  |  |  |  |  |  |  |  |  |  |  |  |  |  |  |  |  |  |  |  |  |  |
| Purhonen 2003 |  |  |  |  |  |  |  |  |  |  |  |  |  |  |  |  |  |  |  |  |  |  |  |  |  |  |  |  |  |
| Purhonen 2006 |  |  |  |  |  |  |  |  |  |  |  |  |  |  |  |  |  |  |  |  |  |  |  |  |  |  |  |  |  |
| Sadrolsadat 2008 |  |  |  |  |  |  |  |  |  |  |  |  |  |  |  |  |  |  |  |  |  |  |  |  |  |  |  |  |  |
| Scifres 2011 |  |  |  |  |  |  |  |  |  |  |  |  |  |  |  |  |  |  |  |  |  |  |  |  |  |  |  |  |  |
| Shaefi 2021 |  |  |  |  |  |  |  |  |  |  |  |  |  |  |  |  |  |  |  |  |  |  |  |  |  |  |  |  |  |
| Šimurina 2010 |  |  |  |  |  |  |  |  |  |  |  |  |  |  |  |  |  |  |  |  |  |  |  |  |  |  |  |  |  |
| Staehr 2011 |  |  |  |  |  |  |  |  |  |  |  |  |  |  |  |  |  |  |  |  |  |  |  |  |  |  |  |  |  |
| Staehr 2012 |  |  |  |  |  |  |  |  |  |  |  |  |  |  |  |  |  |  |  |  |  |  |  |  |  |  |  |  |  |
| Stall 2013 |  |  |  |  |  |  |  |  |  |  |  |  |  |  |  |  |  |  |  |  |  |  |  |  |  |  |  |  |  |
| Thibon 2012 |  |  |  |  |  |  |  |  |  |  |  |  |  |  |  |  |  |  |  |  |  |  |  |  |  |  |  |  |  |
| Treschan 2005 |  |  |  |  |  |  |  |  |  |  |  |  |  |  |  |  |  |  |  |  |  |  |  |  |  |  |  |  |  |
| Turan 2006 |  |  |  |  |  |  |  |  |  |  |  |  |  |  |  |  |  |  |  |  |  |  |  |  |  |  |  |  |  |
| Wadhwa 2014 |  |  |  |  |  |  |  |  |  |  |  |  |  |  |  |  |  |  |  |  |  |  |  |  |  |  |  |  |  |
| Wasnik 2015 |  |  |  |  |  |  |  |  |  |  |  |  |  |  |  |  |  |  |  |  |  |  |  |  |  |  |  |  |  |
| Williams 2013 |  |  |  |  |  |  |  |  |  |  |  |  |  |  |  |  |  |  |  |  |  |  |  |  |  |  |  |  |  |

***Overlap of primary studies in reviews comparing NIV vs COT***

| Study | Hui 2021 | Liu 2020 | Odor 2020 | Wu 2020 | Zayed 2020 | Arora 2020 | Torres 2019 | Pang 2017 | Pieczkoski 2017 | Tong 2017 | Singh 2016 | Zhu 2016 | Faria 2015 | Claire 2014 | Olper 2013 | Nagappa 2014 | Glossop 2012 | Ferreyra 2008 |
| --- | --- | --- | --- | --- | --- | --- | --- | --- | --- | --- | --- | --- | --- | --- | --- | --- | --- | --- |
| Abrard 2021 |  |  |  |  |  |  |  |  |  |  |  |  |  |  |  |  |  |  |
| Alexandropoulou 2019 |  |  |  |  |  |  |  |  |  |  |  |  |  |  |  |  |  |  |
| Al jaaly 2013 |  |  |  |  |  |  |  |  |  |  |  |  |  |  |  |  |  |  |
| AL-Mutairi 2012 |  |  |  |  |  |  |  |  |  |  |  |  |  |  |  |  |  |  |
| Antonelli 2000 |  |  |  |  |  |  |  |  |  |  |  |  |  |  |  |  |  |  |
| Araújo-Filho 2017 |  |  |  |  |  |  |  |  |  |  |  |  |  |  |  |  |  |  |
| Auriant 2001 |  |  |  |  |  |  |  |  |  |  |  |  |  |  |  |  |  |  |
| Barbagallo 2012 |  |  |  |  |  |  |  |  |  |  |  |  |  |  |  |  |  |  |
| Bohner 2002 |  |  |  |  |  |  |  |  |  |  |  |  |  |  |  |  |  |  |
| Carlsson 1981 |  |  |  |  |  |  |  |  |  |  |  |  |  |  |  |  |  |  |
| Cavalcanti 2018 |  |  |  |  |  |  |  |  |  |  |  |  |  |  |  |  |  |  |
| Celebi 2008 |  |  |  |  |  |  |  |  |  |  |  |  |  |  |  |  |  |  |
| Chen 2007 |  |  |  |  |  |  |  |  |  |  |  |  |  |  |  |  |  |  |
| Damgaard 1982 |  |  |  |  |  |  |  |  |  |  |  |  |  |  |  |  |  |  |
| Danner 2012 |  |  |  |  |  |  |  |  |  |  |  |  |  |  |  |  |  |  |
| Ebeo 2002 |  |  |  |  |  |  |  |  |  |  |  |  |  |  |  |  |  |  |
| Ferando 2018 |  |  |  |  |  |  |  |  |  |  |  |  |  |  |  |  |  |  |
| Franco 2011 |  |  |  |  |  |  |  |  |  |  |  |  |  |  |  |  |  |  |
| Olsén 2002 |  |  |  |  |  |  |  |  |  |  |  |  |  |  |  |  |  |  |
| Garutti 2014 |  |  |  |  |  |  |  |  |  |  |  |  |  |  |  |  |  |  |
| Gao 2002 |  |  |  |  |  |  |  |  |  |  |  |  |  |  |  |  |  |  |
| Gupta 2016 |  |  |  |  |  |  |  |  |  |  |  |  |  |  |  |  |  |  |
| Hernandez 2018 |  |  |  |  |  |  |  |  |  |  |  |  |  |  |  |  |  |  |
| Hewidy 2016 |  |  |  |  |  |  |  |  |  |  |  |  |  |  |  |  |  |  |
| Jaber 2016 |  |  |  |  |  |  |  |  |  |  |  |  |  |  |  |  |  |  |
| Jousella 1994 |  |  |  |  |  |  |  |  |  |  |  |  |  |  |  |  |  |  |
| Kindgen-Milles 2005 |  |  |  |  |  |  |  |  |  |  |  |  |  |  |  |  |  |  |
| Kilic 2017 |  |  |  |  |  |  |  |  |  |  |  |  |  |  |  |  |  |  |
| Liao 2010 |  |  |  |  |  |  |  |  |  |  |  |  |  |  |  |  |  |  |
| Linder 1987 |  |  |  |  |  |  |  |  |  |  |  |  |  |  |  |  |  |  |
| Lotz 1984 |  |  |  |  |  |  |  |  |  |  |  |  |  |  |  |  |  |  |
| Lorut 2014 |  |  |  |  |  |  |  |  |  |  |  |  |  |  |  |  |  |  |
| Ludwig 2011 |  |  |  |  |  |  |  |  |  |  |  |  |  |  |  |  |  |  |
| Mamo 2019 |  |  |  |  |  |  |  |  |  |  |  |  |  |  |  |  |  |  |
| Matte 2000 |  |  |  |  |  |  |  |  |  |  |  |  |  |  |  |  |  |  |
| Mazullo 2010 |  |  |  |  |  |  |  |  |  |  |  |  |  |  |  |  |  |  |
| O'Gorman 2013 |  |  |  |  |  |  |  |  |  |  |  |  |  |  |  |  |  |  |
| Palleschi 2018 |  |  |  |  |  |  |  |  |  |  |  |  |  |  |  |  |  |  |
| Pasquina 2004 |  |  |  |  |  |  |  |  |  |  |  |  |  |  |  |  |  |  |
| Pearse 2021 |  |  |  |  |  |  |  |  |  |  |  |  |  |  |  |  |  |  |
| Pessoa 2010 |  |  |  |  |  |  |  |  |  |  |  |  |  |  |  |  |  |  |
| Perrin 2007 |  |  |  |  |  |  |  |  |  |  |  |  |  |  |  |  |  |  |
| Pinilla 1990 |  |  |  |  |  |  |  |  |  |  |  |  |  |  |  |  |  |  |
| Puente-maestu 2021 |  |  |  |  |  |  |  |  |  |  |  |  |  |  |  |  |  |  |
| Ricksten 1986 |  |  |  |  |  |  |  |  |  |  |  |  |  |  |  |  |  |  |
| Squadrone 2005 |  |  |  |  |  |  |  |  |  |  |  |  |  |  |  |  |  |  |
| Stephan 2015 |  |  |  |  |  |  |  |  |  |  |  |  |  |  |  |  |  |  |
| Stock 1984 |  |  |  |  |  |  |  |  |  |  |  |  |  |  |  |  |  |  |
| Stock 1985 |  |  |  |  |  |  |  |  |  |  |  |  |  |  |  |  |  |  |
| Wong 2011 |  |  |  |  |  |  |  |  |  |  |  |  |  |  |  |  |  |  |
| Zarbock 2009 |  |  |  |  |  |  |  |  |  |  |  |  |  |  |  |  |  |  |
| Zhu 2013 |  |  |  |  |  |  |  |  |  |  |  |  |  |  |  |  |  |  |

***Overlap of primary studies in reviews comparing HFNO vs COT***

| Study | Hui 2021 | Wang 2021 | Chaudhuri 2020 I | Lu 2020 | Wang 2020 | Xiang 2020 | Zayed 2020 | Lu 2019 | Zhu 2019 | Du 2018 | Huang 2018 | Wu 2018 | Zhu 2017 | Monroe-Somerville 2016 |
| --- | --- | --- | --- | --- | --- | --- | --- | --- | --- | --- | --- | --- | --- | --- |
| Ansari 2016 |  |  |  |  |  |  |  |  |  |  |  |  |  |  |
| Brainard 2017 |  |  |  |  |  |  |  |  |  |  |  |  |  |  |
| Corley 2015 |  |  |  |  |  |  |  |  |  |  |  |  |  |  |
| Ferrando 2019 |  |  |  |  |  |  |  |  |  |  |  |  |  |  |
| Futier 2016 |  |  |  |  |  |  |  |  |  |  |  |  |  |  |
| Geng 2017 |  |  |  |  |  |  |  |  |  |  |  |  |  |  |
| Parke 2013 |  |  |  |  |  |  |  |  |  |  |  |  |  |  |
| Pennisi 2019 |  |  |  |  |  |  |  |  |  |  |  |  |  |  |
| Pibul 2021 |  |  |  |  |  |  |  |  |  |  |  |  |  |  |
| Sahin 2018 |  |  |  |  |  |  |  |  |  |  |  |  |  |  |
| Sun 2017 |  |  |  |  |  |  |  |  |  |  |  |  |  |  |
| Stephan 2017 |  |  |  |  |  |  |  |  |  |  |  |  |  |  |
| Stephan 2015 |  |  |  |  |  |  |  |  |  |  |  |  |  |  |
| Tatsuishi 2019 |  |  |  |  |  |  |  |  |  |  |  |  |  |  |
| Yang 2017 |  |  |  |  |  |  |  |  |  |  |  |  |  |  |
| Yu 2017 |  |  |  |  |  |  |  |  |  |  |  |  |  |  |
| Zochios 2018 |  |  |  |  |  |  |  |  |  |  |  |  |  |  |
