# Supplementary material for: Perioperative oxygen therapy: an overview of systematic reviews and meta-analyses
Source: Br J Anaesth. 2025 Jun 6;135(5):1456–76. doi: 10.1016/j.bja.2025.04.020 (PMC12597348; doi:10.1016/j.bja.2025.04.020)
Supplement: Supplementary material 4 [file mmc4.docx]

**Supplementary file 4: characteristics of non-anchoring reviews**

***Characteristics of non-anchoring reviews***

| **Review ID** | **Date of search** | **Number of Included RCTS (number of participants included)** | **Patient population (age group)** | **Intervention/comparator** | **Relevant outcomes** | **Study findings** | **Reasons for not being selected as anchoring review** |
| --- | --- | --- | --- | --- | --- | --- | --- |
| Al-Niaimi 2008 | Inception to September 15^th^ 2007 | 4 (989) | Patient undergoing colorectal surgery (adults) | High FiO_2_ (80%)/low FiO_2_ (30-35%) | Incidence of SSI; mortality; length of hospital and ICU stay | Supplemental perioperative oxygenation resulted in a reduced incidence of SSI [RR 0.70 (95% CI 0.52–0.94), P = 0.01], using a fixed effects model. Using the random effects model, the point estimate was similar [RR 0.74 (95% CI 0.39–1.43), P = 0.37], but the results failed to achieve statistical significance. | All trials and data included in more recent review |
| Brar 2011 | Not reported | 5 (1240) | Patients undergoing colorectal surgery  (adults) | High FiO_2_ (80%)/ low FiO_2_ (30-35%) | Incidence of SSI; mortality rate; length of hospital and ICU stay | ‘‘Perioperative supplemental oxygen in colorectal surgery does not significantly reduce SSI. However, supplemental oxygen appears to confer a mortality benefit, a previously unreported finding.  Further RCTs are required to confirm these conclusions.” | All trials and data included in more recent reviews |
| Chu 2018 | Inception to October 25^th^ 2017 | 25 (of which 3 met our inclusion criteria) | Acutely ill patients  (adults) | Liberal oxygen therapy (as defined by study authors)/ Conservative oxygen therapy (as defined by study authors | Incidence of SSI and pneumonia; length of stay; in-hospital mortality, 30-day mortality and mortality at longest point of follow up | Authors do not offer a conclusion on the effect of liberal oxygen therapy. Examining the data, patients who had emergency surgery had fewer hospital-acquired  infections when treated with liberal oxygen therapy  (two randomised controlled trials, n=449, RR 0·50 [95% CI 0·36–0·69], p<0·0001, low quality) than patients treated with conservative therapy. | Only 3 trials of interest were included in this review (included in other reviews) |
| Cohen 2018 | Update on Wetterslev et al.- inception to January 2017 | 26 (14710) | Patients undergoing a mix of colorectal and non-colorectal surgery  (adults and children) | High FiO_2_ (80%)/ Low FiO_2_ (30%) | Incidence of SSI | Intraoperative hyperoxia reduced the incidence of SSI [RR, 0.81 95% CI 0.70, 0.94]. The effect remained significant in colorectal patients (N=10,469), 0.79 [0.66, 0.96], but not in other patients (N=4,241), 0.86 [0.69, 1.09]. No significant benefit was found when restricting the analysis to studies judged to be at low risk of bias | Considered for inclusion as anchoring review. Excluded following group consensus that other reviews were more appropriate as anchoring reviews. |
| Dhalke 2013 | January 1^st^ 2005- September 1^st^ 2012 | 2 | Caesarean section | High FiO_2_ (80-100%)/ 2 litres oxygen via nasal cannula | This review evaluated the evidence to recommend or reject certain interventions in the conduct of Caesarean section delivery | Only 2 trials reported a reduction in morbidity from infection among groups. | The 2 trials of interest are included in a separate review |
| Fasquel 2020 | January 1^st^ 1999 to February 1^st^ 2020 | 21 | Adults undergoing surgery- most commonly abdominal including caesarean section  (adults) | High FiO_2_ (80%)/ low FiO_2_ (30%) | Incidence of SSI at 15 or 30 days, mortality rate, incidence of postoperative pulmonary complications, length of hospital stay, incidence of postoperative nausea and vomiting | Results were heterogeneous but most recent studies and the largest RCTs reported no  difference in the incidence of SSI between the two groups. The review on secondary outcomes (respiratory and cardiovascular complications, postoperative nausea and vomiting, length of hospital stay and mortality) also failed to support the use of high FiO_2_. On the opposite, some data from follow-up analyses and registry studies suggested a possible negative effect of high FiO_2_ on long-term outcomes. | Qualitative analysis only |
| Hovaguimian 2013 | Inception to September 2012 | 22 (7001) | Adults undergoing surgery  (adults) | High FiO_2_ (80-100%)/ low FiO_2_ (30-40%) | Incidence of SSI, postoperative pulmonary complications and PONV | In nine trials including 5,103 patients, the incidence of SSI decreased from 14.1% with normal FiO_2_ to 11.4% with high FiO_2_; RR,0.77 (95% CI, 0.59–1.00). In patients undergoing colorectal surgery, the incidence of SSI decreased from 19.3 to 15.2%; RR, 0.78 (95% CI, 0.60–1.02). The incidence of postoperative nausea and vomiting decreased from 24.8% with normal FiO_2_ to 19.5% with high FiO_2_; RR, 0.79 (95% CI, 0.66– 0.93). Nine trials including 3,698 patients reported on pulmonary outcomes. High FiO_2_ did not increase the risk of atelectasis. | Trials and data on SSI were included in other more recent reviews. |
| Kao 2012 | Inception to June 2011 | 8 (4778) | Patients undergoing surgery  (adults) | High FiO_2_ (80%)/ low FiO_2_ (30-35%) | Incidence of SSI; mortality | “There is a moderately high probability of a benefit to hyperoxia in reducing SSIs in colorectal surgery patients; however, the magnitude of benefit is relatively small and might not exceed treatment hazards.” | All trials and data were included in subsequent reviews |
| Klingel 2013 | Inception to December 2011 | 5 (1966) | Patients undergoing Caesarean section  (adults) | High FiO_2_ (80%)/ Low FiO_2_ (30%). One trial used room air as control | Composite outcome of SSI and endometritis | “There is no evidence to suggest a difference in risk of surgical site infection by administration of high inspired oxygen concentrations among women undergoing caesarean section. Future studies with better adherence to the intervention may affect the results of this analysis.” | All trials and data included in subsequent review |
| Koo 2019 | From inception to August 2018 | 10 (787) | Patients undergoing surgery (adults) | High FiO_2_ (80-100%)/ low FiO_2_ (30-35%) | Incidence of postoperative pulmonary complications | “The results of this meta-analysis suggest that high inspired oxygen fraction during anaesthesia may impair postoperative pulmonary parameters. Cautious approach in intraoperative inspired oxygen fraction is required for patients susceptible to postoperative pulmonary complications.” | All the papers included in this review are also included in Lim 2021 which therefore supersedes this review |
| Martin 2015 | Inception to March 2013 | 10 (427). Of these, 5 RCTs met this overview’s inclusion criteria | Patients undergoing surgery  (adults) | High FiO_2_ (>60%)/ low FiO_2_ (<60%) | Incidence of postoperative atelectasis | Narrative synthesis. There were five studies that compared the use of high FiO_2_ to a decreased FiO_2_ (< 60%). Four of the five articles found no significant difference between the two groups in the incidence of atelectasis | Narrative synthesis |
| Mattishent 2019 | Inception to July 2018 | 17 (7050) | Patients undergoing surgery  (adults) | High FiO_2_ (80%)/ Low FiO_2_ (30-35%) | Short- and long-term mortality; incidence of atelectasis, pneumonia, other respirator adverse events, rates of unplanned admission to ICU | No evidence of harm with high FiO_2_ was found for major adverse events: mortality RR 0.49 (95% CI 0.17-1.37), atelectasis RR 0.91 (95% CI 0.59-1.42); cardiovascular events RR 0.90 (95% CI 0.32-2.54) and ICU admission RR 0.93 (95% CI 0.7-1.12). | Considered as anchoring review. Group consensus that Lim 2021 was more comprehensive |
| Mejía 2007 | Different search ranges in different databases: 1950-2006 | 4 (998) | Patients undergoing elective colorectal surgery  (adults) | High FiO_2_ (80%)/ low FiO_2_  (≤35%) | Incidence of SSI within 15 days of surgery; length of hospital stay; rates of admission to ICU; mortality | The authors conclude that their analysis does not support the use of high flow supplemental oxygen to reduce surgical site infection in elective patients undergoing abdominal surgery | All data and trials were included in subsequent reviews |
| Orhan-Sungur 2008 | 1996-March 2006 | 10 (1729) | Patients undergoing surgery  (adults and children) | High FiO_2_ (80%)/ low FiO_2_ (30-40%) | Incidence of PONV | “The positive results of two initial studies reducing the risk for PONV in patients given 80% FiO_2_ were not confirmed by any of the subsequent trials. Considering all available evidence, 80% FiO_2_ should no longer be considered an effective or reliable method to reduce overall PONV.” | All data and trials included in subsequent reviews |
| Patel 2013 | Inception to 2011 | 6 (2613) | Patients undergoing open abdominal surgery  (adults) | High FiO_2_ (>60%)/ low FiO_2_ (≤40%) | Incidence of SSI | There was no evidence of a reduction in SSIs with high FiO_2_ in patients undergoing open abdominal surgery  (RR 0.77, 95% confidence interval 0.50–1.19). Substantial het- erogeneity was observed among studies. | All trials and data included in a more up to date review |
| Qadan 2009 | 1^st^ 1966 to September 30^th^ 2007 | 5 (2803) | Patients undergoing any type of surgery with supplemental oxygen  (adults) | High FiO_2_ (80%0/ low FiO_2_ (<30%) via nasal cannula or face mask | Incidence of SSI within 14 days | Infection rates were 12.0% in the low FiO_2_ group and 9.0% in the high FiO_2_ group, with relative risk reduction of 25.3% (95% CI, 8.1%-40.1%) and absolute risk reduction of 3.0% (1.1%- 5.3%). The overall RR was 0.74 (95% CI, 0.59- 0.91; P=.006). The benefit from increasing oxygen concentration was greater in patients undergoing colorectal surgery[ RR 0.55 (95% CI, 0.38-0.80)]. | All trials and data on SSI are included in other more recent reviews |
| Rincón Valenzuela 2012 | Timeframe of searches not reported | 17 studies (4844) | Patients undergoing surgery  (adults and children) | High FiO_2_ (>60%)/ Low FiO_2_ (<40%) | Incidence of SSI and pneumonia; mortality; rates of unplanned ITU admission; length of hospital stay; overall rate of PONV. | High FiO2 reduced post-operative nausea and vomiting (odds ratio [OR] 0.40; 95% CI , 0.20 to 0.80), SSI (OR 0.46; 95% CI, 0.29 to 0.74), and mortality (OR 0.17; 95% CI, 0.03 to 0.99) in surgeries with extensive intestinal manipulation. | Trials and data on PONV, SSI and length of hospital stay are included in other most recent reviews. |
| Shaffer 2021 | Inception to August 2017 | 11 (8245) | Adult patients undergoing colorectal surgery  (adults) | High FiO_2_ (80%)/ low FiO_2_ (30-35%) | Incidence of SSI and atelectasis. Length of hospital stay. | 80% FIO2 was not effective in reducing SSI (RR, 0.91; 95% CI, 0.74 to 1.13; moderate certainty evidence). No effect on length of stay was found (MD, 0.39; 95% CI -0.59 to 1.36; low certainty evidence). | Limited to one category of surgical procedure. Of the 11 included trials one was a case control study, and one was retracted. |
| Smith 2020 | Inception until October 2018 | 12 (10212) | Patients undergoing any type of surgery  (adults) | High FiO_2_ (80%)/ low FiO_2_ (30%) | Incidence of SSI at 30 days, 30-day mortality | No statistically significant difference in postoperative SSI or mortality was found when comparing patients receiving an FiO2 of 80% to those receiving an FiO2 of 30%. | Our chosen anchoring reviews (de Jonge 2019, Lim 2021) provide a more comprehensive review on SSI and mortality including subgroup and sensitivity analysis |
| Togioka 2012 | Inception to July 12^th^, 2020 | 7 (2728) | Patients undergoing abdominal surgery  (adults and adolescents over the age of 15) | High FiO_2_ (80%)/ low FiO_2_ (30-35%) | Incidence of SSI | Hyperoxia resulted in an OR of 0.85 for surgical site infection (95% confidence interval: 0.52, 1.38). However, two subgroup analyses (general anesthesia and colorectal surgery trials) showed a benefit for high FiO_2_ in decreasing SSI. | All trials and data included in other reviews |
| Wang 2017 | From inception to August 1^st^, 2015 | 12 (6750) | Surgical patients  (adults) | High FiO_2_ (≥80%)/ low FiO_2_ (30-35%) | Incidence of SSI at 14 and 30 days | No significant difference in the incidence of SSIs was found between the two groups [RR: 0⋅91; 95% CI: 0⋅72–1⋅14]. A subgroup analysis of studies with intestinal tract surgery showed that patients experienced less SSI when high FiO_2_ was administrated (RR: 0⋅53; 95% CI: 0⋅37–0⋅74; P = 0⋅0003). | All trials and data included in more recent reviews. |
| Wetterslev 2015 | Inception to March 2015 | 28 (9330) | Patients undergoing surgery  (adults) | High FiO_2_ (60-90%)/ low FIO_2_ (30-40%) | Incidence of SSI at 30 days, 30-day mortality, mortality at longest point of follow-up, incidence of respiratory insufficiency, length of stay | ‘‘Overall results suggest that evidence is insufficient to support the routine use of a high fraction of inspired oxygen during anaesthesia and surgery’’ | Data presented in this review are included in more recent reviews |
| Xiao 2019 | Inception to April 2019 | 95 (not all including surgical patients) | Any patient population  (adults, children and neonates) | Normobaric oxygen therapy delivered according to different criteria/ standard care | Mortality, incidence of SSI, respiratory failure, pneumonia and atelectasis. Rates of intubation and nausea/vomiting, | Contrary to the WHO recommendation, 80% FiO2 did not affect surgical site infection in intubated patients (RR, 0.84; 95% CI, 0.69 to 1.01; p=0.07; moderate quality) | Data from included surgical trials are included in other more focused review |
| Yang 2016 | Inception to December 2015 | 17 (8093) | Patients undergoing abdominal surgery  (adults) | High FiO_2_ (80%)/ low FiO_2_ (30-35%) | Incidence of SSI. | “This meta- analysis, which used stricter inclusion and exclusion criteria, found that high FiO_2_ does not significantly reduce the SSI rate. Except for in colorectal surgery.” | Included studies are reported in more up to date review |
| Zhang 2016 | Inception to March 2015 | 9 RCTs (3281) | Patients undergoing abdominal surgery  (adults) | High FiO_2_ (80%)/ FiO_2_ of 30% | Incidence of SSI. | No significant difference in SSI was found between the two groups; RR=0.80, 95% CI: 0.60-1.08, P=0.15. However, the results of subgroup analyses showed that high FiO_2_ decreased the incidence of SSI significantly in the subgroups of colorectal surgery and intraoperative plus postoperative 6 h oxygen inhalation. | Included studies are reported in more up to date review |
| Zhao 2016 |  | 13 RCTs (3532) | Patients undergoing abdominal surgery  (adults) | High FiO_2_ (70%-80%)/ FiO_2_ of 30-35% | Incidence of SSI, 30-day mortality, atelectasis | The incidence SSI in the high FiO_2_ group was lower than that in the control group (OR=0.68, 95%CI 0.47 to 0.99). No significant differences were found between the groups in incidence of atelectasis, and 30-day mortality (all P values >0.05). | Included studies are reported in more up to date review |
| Du 2018 | January 2000 to November 2016 | 4 (643) | Patients undergoing cardiac surgery | HFNO/COT | Incidence of atelectasis; length of ITU stay; reintubation rates | “Compared with COT, HFNO could reduce the rate of tracheal reintubation in patients after cardiac surgeries, but no difference was found in improving atelectasis or reducing the length of ICU stay” | All trials and data on the reported outcomes are included in a more recent and comprehensive review (Chaudhuri 2020) |
| Huang 2018 | Inception to November 2016 | 7 (2781) | Patients undergoing cardiothoracic and major abdominal surgery  (adults) | HFNO/COT or non-invasive ventilation (NIV) | Reintubation rate; LOS; mortality | HFNO had a similar reintubation rate compared to COT (RR, 0.58; 95% CI, 0.21-1.60) or NIV (RR, 1.11; 95% CI, 0.88-1.40). In subgroup of critically ill patients, HFNO had a significantly lower reintubation rate compared to the COT (RR, 0.35; 95% CI, 0.19-0.64; interaction P = .07). | All trials and data on the reported outcomes are included in a more recent and comprehensive review (Chaudhuri 2020 I) |
| Lu 2019 | Inception to August 31^st,^ 2018 | 7 (965) | Surgical patients following extubation  (adults) | HFNO/COT | Mortality (in-hospital or at 28 days); incidence of PPC; reintubation rate; rates of exalation of respiratory support | HFNO significantly reduced reintubation rate (RR 0.39, 95%CI 0.17 to 0.87,) and rate of escalation of respiratory support (RR 0.46, 95%CI 0.22 to 0.93) in post-extubation surgical patients compared with COT. No differences in the incidence of PPCs (RR 0.86, 95%CI 0.69 to 1.086) or mortality (RR 0.77, 95%CI 0.17 to 3.41) were found. | Addresses the same research question of another more recent review |
| Lu 2020 | Inception to July 31^st^ 2018 | 14 (2568) | Patients undergoing surgery  (adults) | HFNO/COT or NIV | Mortality; rates of postoperative pulmonary complications; reintubation rate, LOS | HFNO was significantly associated with a shorter length of hospital stay (MD: 0.81; 95% CI: 1.34 to 0.29), but not mortality (RR: 1.0, 95% CI: 0.63 to 1.59). There was weak evidence of a reduction in reintubation rate (RR: 0.76, 95% CI: 0.57-1.01) and PPC rate (RR: 0.89, 95% CI: 0.75-1.06). | Considered as possible anchoring review as recent, addresses the same research question as Chaudhuri 2020 I and includes further papers. Upon group discussion was excluded as anchoring review as some of the extra trials pertain to a separate comparison |
| Monro-Somerville 2017 | Search timeframe not reported | 14 (2507) | Patients suffering with ARF (any aetiology)  (adults) | HFNO/COT via face mask or NIV | Mortality; reintubation rate | No difference in mortality (OR, 0.83; 95% CI, 0.58–1.17) or intubation rate (OR, 0.63; 95% CI, 0.37–1.06) was detected in patients with acute respiratory failure treated with HFNO compared with usual care. HFNO seem well tolerated by patients. Further large RCTs are required to evaluate their utility in this setting. | Mixed cohort. Surgical trials and data on the reported outcomes are included in a more recent and comprehensive review ( Chaudhuri 2020 I) |
| Wang 2020 | Inception to November 2019 | 8 (1086) | Patients who have undergone cardiothoracic surgery  (adults) | HFNO/COT | In-hospital mortality; incidence of postoperative respiratory failure (defined as escalation of respiratory support); length of hospital stay; rates of unplanned ICU admission | “HFNO may significantly reduce the need for the escalation of respiratory support and re-intubation rate, and might reduce the hospital stay.” | All trials and data reported are included in a more comprehensive review |
| Wang 2021 | Inception to April 10^th^ 2021 | 3 (526) | Obese patients undergoing cardiac surgery  (adults) | HFNO/COT | Incidence of atelectasis. Length of ICU stay. Rates of reintubation | “'For obese patients undergoing cardiac surgery, postoperative use of HFNO can maintain patient’s oxygenation. Additional clinical studies are needed to investigate the role of HFNO in this patient group.” | All trials and data on the reported outcomes are included in a more comprehensive review |
| Wu 2018 | Inception to December 2017 | 4 (649) | Patients in the postoperative period after cardiothoracic surgery  (adults) | HFNO/COT | Incidence of atelectasis and pneumonia; reintubation rate; rate of escalation of respiratory support; length of ICU and hospital stay | “The HFNO could reduce respiratory support and pulmonary complications, and it could be safely administered for adult post cardiothoracic surgery. Further large-scale, randomized, and controlled trials are needed to update this finding.” | No- all trials and data on the reported outcomes are included in a more recent and comprehensive review (Chaudhuri 2020 I) |
| Xiang 2020 | Inception to July 2019 | 6 (733) | Postoperative patients at high risk for pulmonary complications  (adults) | HFNO/COT | Mortality; incidence of pulmonary complications; length of ICU and hospital stay; escalation of therapy defined as rate of intubation or need for NIV for respiratory failure | Compared with COT, HFNO could reduce intubation rate (RR 0.23, 95% CI 0.08-0.66) , but could not reduce either Hospital or ICU LOS. | All trials and data included in more comprehensive review (anchoring review) |
| Zhu 2017 | Inception to June 2016 | 2 (495) | Patients undergoing cardiac surgery  (adults) | HFNO/COT | Rate of escalation of respiratory support; reintubation rates; length of ICU stay | “Our meta-analysis demonstrated that HFNO could reduce the need for the escalation of respiratory support, and it could be safely administered in adult postextubation cardiac surgical patients. Further large- scale, multicentre studies are required to confirm our results.” | All trials and data included in more comprehensive and more recent review. Very specific to cardiac surgery. |
| Zhu 2019 | Inception to August 2018 | 10 (7 RCTs and 3 cross-over studies) (1708) | Patients who had planned extubation following mechanical ventilation in the hospital or ICU (adults) | HFNO/COT | ICU and in-hospital mortality; incidence of postoperative respiratory failure; reintubation rate; length of ICU and hospital stay | “Our meta-analysis demonstrated that compared with COT therapy, HFNO therapy may significantly reduce postextubation respiratory failure and respiratory rates, may increase PaO_2_, and may be safely administered in patients after planned extubation.” | Mixed cohort of medical and surgical patients. Trials including surgical patients are included in another more comprehensive review |
| Arora 2020 | Inception to January 2017 | 3 (562) | Patients with respiratory compromise undergoing abdominal surgery  (adults) | NIV/COT | Rates of pneumonia; intubation rate; length of ICU and hospital stay | Based on moderate- to low-certainty evidence, NIV use after abdominal surgery did not result in a significant decrease intubation rates or ICU length of stay, but did reduce the incidence pneumonia. | Very specific review with narrow scope. |
| Ireland 2014 | 1996 to September 2013 | 10 (709) | Patients undergoing major abdominal surgery  (adults) | Postoperative CPAP/usual postoperative care | Incidence of SSI and PPCs; mortality; rates of unplanned ICU admission; hospital LOS | Very low-quality evidence suggests that postoperative CPAP might reduce the incidence of postoperative pneumonia,  atelectasis and reintubation, but its effects on mortality, hypoxia or invasive ventilation are uncertain. Evidence is  insufficient to confirm the benefits or harms of postoperative CPAP in patients undergoing major abdominal surgery. | Considered as possible anchoring review. Rejected following group discussion as scope quite narrow, CPAP only intervention and slightly outdated searches. |
| Faria 2015 | Inception to May 2015 | 2 (269) | Patients with acute respiratory failure after upper abdominal surgery  (adults) | NIV/COT | In-hospital mortality; incidence of postoperative respiratory failure and PPCs; rate of unplanned ICU admission and of tracheal intubation; length of ICU and hospital stay | “CPAP or bilevel NPPV is an effective and safe intervention for the treatment of adults with acute respiratory failure after upper abdominal surgery. However, based on GRADE methodology, the quality of the evidence was low or very low.” | Scope of review too narrow |
| Ferreyra 2008 | January 1996 to November 2005 | 9 (654) | Patients recovering from abdominal surgery  (adults) | CPAP/standard care | Incidence of PPC | CPAP significantly reduced the risk of PPCs (risk ratio, 0.66; 95% confidence interval CI, 0.52– 0.85); atelectasis (RR, 0.75; 95% CI, 0.58 – 0.97); pneumonia (RR, 0.33; 95% CI, 0.14 – 0.75) | All trials and data are included in a subsequent review |
| Glossop 2012 | Inception to January 2012 | 16 (979) | Patients undergoing surgery, critically ill patients in ICU  (adults) | NIV/COT | Mortality; incidence of PPC; rates of reintubation and unplanned ICU admission; ICU and hospital length of stay | NIV use in the postoperative period reduced reintubation rate [OR 0.24, 95% CI 0.12-0.50] and the incidence of pneumonia (OR 0.27, 95% CI 0.09 –0.77). There was insufficient evidence to suggest that NIV improves ICU survival, but might increase hospital survival post-surgery (OR 4.54, 0.95% CI 1.35 –15.31). | All trials and data are included in a subsequent review |
| Liu 2020 | Inception to April 2018 | 10 (1011) | Patients undergoing cardiac surgery  (adults) | CPAP or BiPAP/ COT or other conventional therapy | Mortality; incidence of atelectasis; reintubation rate; other PPCs | “Prophylactic NIV is associated with a lower rate of postoperative pulmonary complications. The effect on the other complications and hospital mortality might be limited. Further evidence with randomized controlled trials can discern the benefits.” | Lack of consensus with other reviews with comparable methodology, thus risk of introducing bias. |
| Nagappa 2014 | 1946 to October 31^st^ 2013 | 2 (263) | Patients with OSA undergoing surgery  (adults) | Preoperative CPAP/ no treatment | Incidence of atelectasis; rate of unplanned ICU admission; hospital LOS | There was no significant difference in the postoperative adverse events between CPAP and no-CPAP treatment groups. | Only 2 relevant RCTs included. Narrative synthesis including non-randomised studies |
| Odor 2020 | 1990 to December 12^th^ 2017 | 10 (1173) | Patients undergoing non-cardiac, non-transplant surgery  (adults) | NIV/COT | In-hospital mortality; incidence of atelectasis and respiratory infection | Postoperative CPAP after major abdominal and thoracic surgery may reduce PPCs (RR 0.49, 0.24 to 0.99); however, included RCTs were small and the required information size was not met. | All trials and data are included in a subsequent review |
| Olper 2013 | Inception to 2012 | 14 (1211) | Patients undergoing cardiac, pulmonary or thoracoabdominal surgery (adults) | NIV/conventional respiratory care | Mortality; incidence of pneumonia and atelectasis; reintubation rate | “NIV seems to be effective in reducing reintubation rate after cardiothoracic surgery. The results of this meta-analysis should be confirmed by large, randomised control studies” | Comprehensive review of NIV in patients undergoing cardiothoracic surgery, however group consensus decision was to select one anchoring review and update meta-analysis as required |
| Pang 2017 | Inception to March 2016 | 21 (842);  2 (37) for oxygen in the perioperative period | Obese patients undergoing surgery  (adults) | NIV/COT via nasal cannula | No relevant outcomes reported | The results showed that the optimal perioperative ventilation strategies in obese patients include head-up position plus CPAP for spontaneous breathing and IPPV with PEEP for mechanical ventilation during induction, large tidal volume ventilation with PEEP during anaesthesia maintenance, and NIPPV applied for oxygen delivery after extubation. | Multiple interventions addressed in this review and only 2 trials included for the comparison of interest. |
| Pieczkoski 2017 | Inception to March 2016 | 10 (1050) in narrative synthesis; 6(771) included in meta-analysis | Patients undergoing cardiac surgery (non-transplant) | NIV/standard therapy | Incidence of atelectasis; reintubation rate; length of ICU stay | Postoperative NIV use did not reduce the risk for atelectasis (RR: 0.60; 95% CI 0.28-1.28); pneumonia (RR: 0.20; 95% CI 0.04-1.16), reintubation rate (RR: 0.51; 95% CI: 0.15-1.66), and time spent in the ICU (-0.04 days; 95% CI: -0.13; 0.05). | All data and trials included in a more recent review. |
| Singh 2016 | Inception until November 4^th^, 2015 | 11 (725) | Patients undergoing upper abdominal surgery  (adults) | CPAP/ oxygen therapy with or without chest physiotherapy | Incidence of pneumonia, atelectasis and PPC | “Postoperative CPAP significantly reduces the incidence of atelectasis, pneumonia, and pulmonary complications in patients undergoing high-risk abdominal surgeries. Increasing the CPAP levels does not necessarily enhance the protective effect against pneumonia. Instead, protective effect decreases with increased CPAP levels. | All data and trials included in a more recent review on this topic |
| Tong 2017 | January 1^st^ 2000 to January 1^st^ 2015 | 5 (204) | Patients with obstructive sleep apnoea (OSA) undergoing bariatric surgery  (adults) | NIV/no intervention | Incidence of PPC | “Results reported in narrative format only. Review did not provide evidence that there is increased anastomotic dehiscence risk when NIPPV is administered during immediate post–bariatric surgery care.” | Narrative review |
| Torres 2019 | Inception to Deceber 2018 | 7 RCTs and 1 quasi-RCTs (486) | Adults undergoing pulmonary resection for lung cancer  (adults) | NIV/standard therapy or no intervention | Mortality; incidence of PPC; intubation rates; hospital LOS | No additional benefit of using NIPPV in the postoperative period after pulmonary resection for all outcomes analysed However, the certainty in evidence was 'very low', 'low' or moderate'. | Very specific population which limits generalisability of results to other surgical patients. |
| Wu 2020 | Inception to July 2019 | 9 (830) | Patients undergoing cardiac surgery with cardiopulmonary bypass  (adults) | CPAP or BiPAP/ standard therapy | Mortality; incidence of atelectasis and pneumonia; reintubation rates; length of ICU and hospital stay | No significant difference in pulmonary complications between NIV and standard therapy in patients undergoing cardiac surgery was found. However, prolonged NIV treatment could reduce incidence of pulmonary complications.  No significant difference in mortality, reintubation rate and cardiac complications was found. | Results conflicting with other reviews with similar methodology and assessing the same body of evidence. Methodological quality insufficient for anchoring review. |
| Zayed 2020 | Inception to September 2019 | 9 (1865) | Patients at high risk of or with established respiratory failure in the postoperative period (adults) | NIV/COT | Mortality; reintubation rate | Compared with COT, postoperative NIV was associated with reduced intubation rate, mortality, and ICU-acquired infections in patients at high risk of respiratory failure. HFNO was associated with reduced rates of intubation and ICU-acquired infections but not mortality in comparison with COT. There was no significant difference between HFNO and NIV on clinical outcomes. | Trials and data are included in more comprehensive review |
| Zhu 2016 | Start of NIV use to 2015 | 14 | Patients undergoing cardiothoracic surgery  (adults) | NIV/standard treatment | Mortality; incidence of PPCs; reintubation rates; hospital LOS | NIV had minimal effect on the risk of mortality (RR: 0.64; 95% CI: 0.36–1.14), endotracheal intubation (RR: 0.52; 95% CI: 0.24–1.11), respiratory (RR: 0.70; 95% CI: 0.47–1.30), cardiovascular (RR: 0.81; 95% CI: 0.54–1.2), and other complications (RR: 0.72; 95% CI: 0.38–1.36). | Wider scope than other reviews investigating use of NIV in cardiothoracic surgery but scope not broad enough to be considered an anchoring review |
