# Supplementary material for: Perioperative oxygen therapy: an overview of systematic reviews and meta-analyses
Source: Br J Anaesth. 2025 Jun 6;135(5):1456–76. doi: 10.1016/j.bja.2025.04.020 (PMC12597348; doi:10.1016/j.bja.2025.04.020)
Supplement: Supplementary material 5 [file mmc5.docx]

**Supplementary file 5: Characteristics of new RCTs**

| Study | Country | No of patients randomised | Type of surgery | Care stage | Intervention vs control | Relevant outcomes |
| --- | --- | --- | --- | --- | --- | --- |
| Alvandipour et al 2019 | Iran | 85 | Colorectal surgery | Intraoperative+2 hours postoperatively | 80% FiO_2_ vs 30% FiO_2_ | SSI, atelectasis, pneumonia, respiratory failure, length of hospital stay |
| Ferrando et al 2020 | Spain | 740 | Abdominal surgery | Intraoperative+3 hours postoperatively | 80% FiO_2_ vs 30% FiO_2_ | Mortality within 30 days, mortality within 180 days, SSI, pulmonary complications |
| Holse et al 2022 | Denmark | 600 | Major noncardiac surgery | Intraoperative+2 hours postoperatively | 80% FiO_2_ vs 30% FiO_2_ | SSI, pneumonia, respiratory failure, mortality |
| Lin et al 2021 | China | 652 | Laparoscopic gastric and colorectal malignancies surgery | Intraoperative | 80% FiO_2_ vs 40% FiO_2_ | SSI, pneumonia, atelectasis |
| Mayank et al 2019 | India | 94 | Colorectal surgery | Intraoperative+6 hours postoperatively | 80% FiO_2_ vs 33% FiO_2_ | SSI |
| Reiterer et al 2021 | Austria | 260 | Major abdominal surgery | Intraoperative+2 hours postoperatively | 80% FiO_2_ vs 30% FiO_2_ | Mortality, unplanned ICU admission, respiratory failure |
| Yerra et al 2021 | India | 178 | Abdominal surgery | Intraoperative+2 hours postoperatively | 80% FiO_2_ vs 30% FiO_2_ | SSI, PONV, respiratory complications |
| Ferrando et al 2019 | Spain | 64 | Laparoscopic bariatric surgery | First 3 hours postoperatively | HFNO at 60L/min vs Venturi mask 15l/min | Atelectasis, hypoxaemia, pneumonia, ARDS, respiratory failure, length of hospital stay |
| Pibul et al 2021 | Thailand | 67 | Cardiac surgery with midline sternotomy | First 24 hours postoperatively | HFNO 20-60L/min vs face mask/nasal cannula | Reintubation rate, length of hospital stay, length of ICU stay |
| Theologou et al 2021 | Greece | 99 | Cardiac surgery | First 48 hours postoperatively | HFNO 60 or 40 L/min vs venturi mask | Reintubation, mortality, escalation of respiratory support |
| Twose et al 2019 | United Kingdom | 20 | Major head and neck surgery | Postoperatively at the point when mechanical ventilation stopped | HFNO of 60 L/minute vs standard O_2_ | Pneumonia, ARDS, length of hospital stay, ICU length of stay |
| Vourc'h et al 2020 | France | 98 | Cardiac surgery | First 48 hours postoperatively | HFNO 45 L/min vs high-flow face mask 15 L/min. | Reintubation, escalation of respiratory support, ICU length of stay |
| METRRC 2022 (Major Extremity Trauma Research Consortium) | USA | 1,136 | Lower-Extremity Fractures | Intraoperative and for up to 2 hours in the recovery room | 80% FiO_2_ vs 30% FiO_2_ | SSI within 182 days following definitive fixation. Secondary outcomes included SSI at 90 and 365 days after surgery. |
| Nam et al. 2023 | Korea | 414 | Cardiac surgery | Intraoperative | 80% FiO2 vs 30% FiO2 | SSI, mortality, length of hospital stay, length of ICU stay |
| Sadurni et al 2023 | Spain | 403 | Colorectal surgery | Intraoperative | FiO2>0.8 vs FiO2<0.4 | SSI, incidence of myocardial injury, major adverse cardiovascular and cerebral events up to 30 postoperative days, length of stay |
| Soliman et al 2022 | Egypt | 87 | Upper abdominal surgery | Postoperative | HFNO vs face mask | Escalation of respiratory support, length of hospital and ICU stay |
