# Supplementary material for: Perioperative oxygen therapy: an overview of systematic reviews and meta-analyses
Source: Br J Anaesth. 2025 Jun 6;135(5):1456–76. doi: 10.1016/j.bja.2025.04.020 (PMC12597348; doi:10.1016/j.bja.2025.04.020)
Supplement: Supplementary material 7 [file mmc7.docx]

**Supplementary file 7: risk of bias assessment for additional RCTs.**
