# Supplementary material for: Perioperative oxygen therapy: an overview of systematic reviews and meta-analyses
Source: Br J Anaesth. 2025 Jun 6;135(5):1456–76. doi: 10.1016/j.bja.2025.04.020 (PMC12597348; doi:10.1016/j.bja.2025.04.020)

**Supplementary file 9: Trial sequential analysis of surgical site infection in low risk of bias trials included in the meta-analysis.**


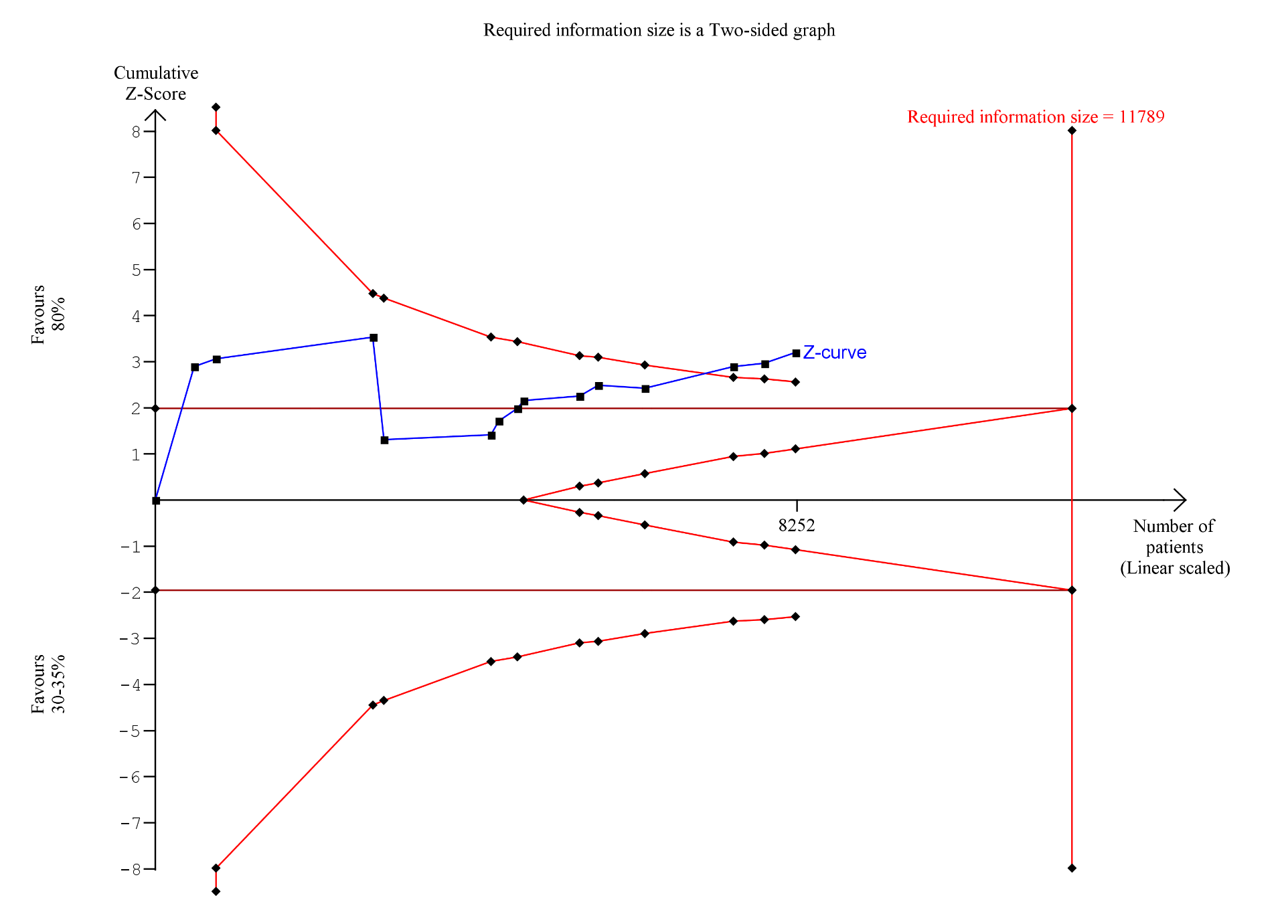

Supplement: Supplementary material 9 [file mmc9.docx]
