# Supplementary material for: Perioperative oxygen therapy: an overview of systematic reviews and meta-analyses
Source: Br J Anaesth. 2025 Jun 6;135(5):1456–76. doi: 10.1016/j.bja.2025.04.020 (PMC12597348; doi:10.1016/j.bja.2025.04.020)

**Supplementary file 10: funnel plot analyses**

***Funnel plot analysis for publication bias in reporting SSI incidence between 80% and 30-35% supplemental oxygen.***


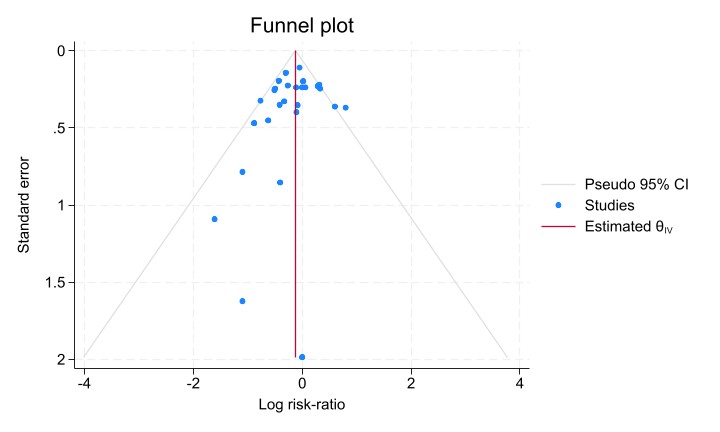


***Funnel plot analysis for publication bias in reporting mortality incidence within 30 days between high and low FiO2.***


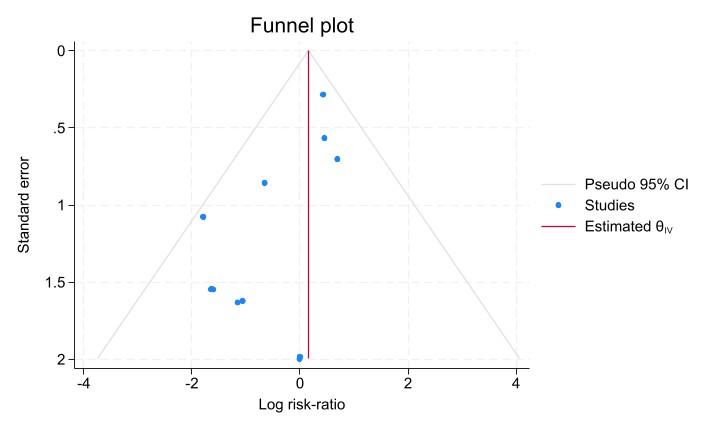


***Funnel plot analysis for publication bias in reporting mortality incidence up to longest follow up between high and low FiO2.***


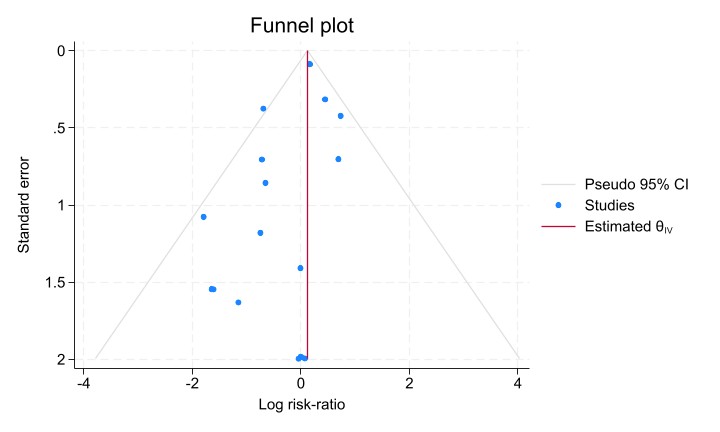


***Funnel plot analysis for publication bias in reporting length of hospital stay between high and low FiO2.***


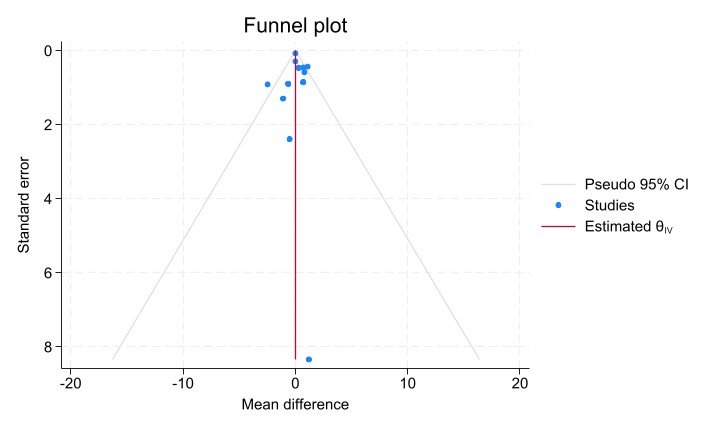


***Funnel plot analysis for publication bias in reporting escalation of respiratory support between HFNO and COT.***

***
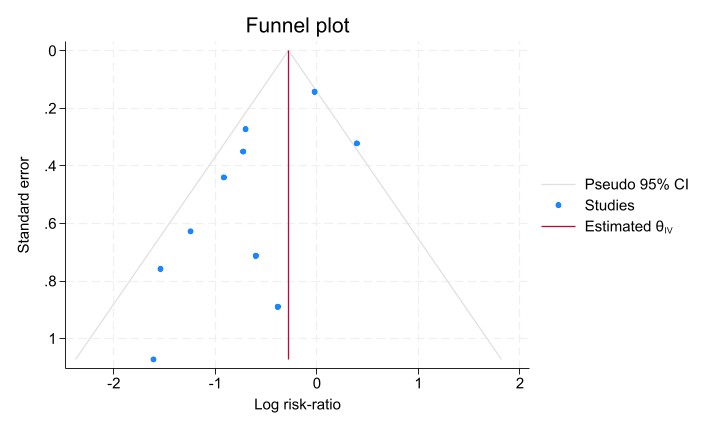
***

***Funnel plot analysis for publication bias in reporting length of hospital stay between HFNO and COT.***


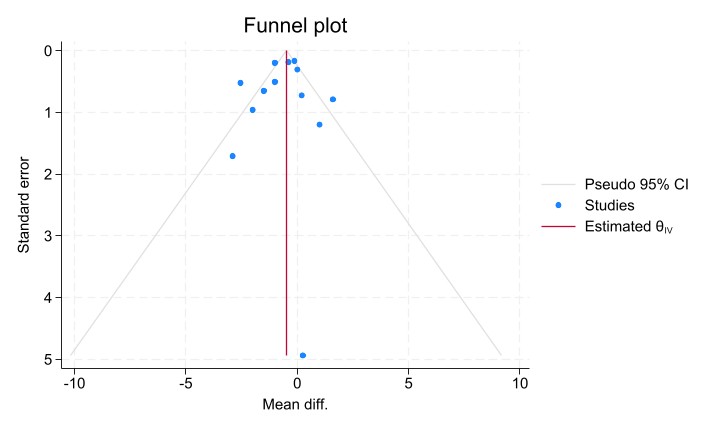


***Funnel plot analysis for publication bias in reporting length of ICU stay between HFNO and COT.***


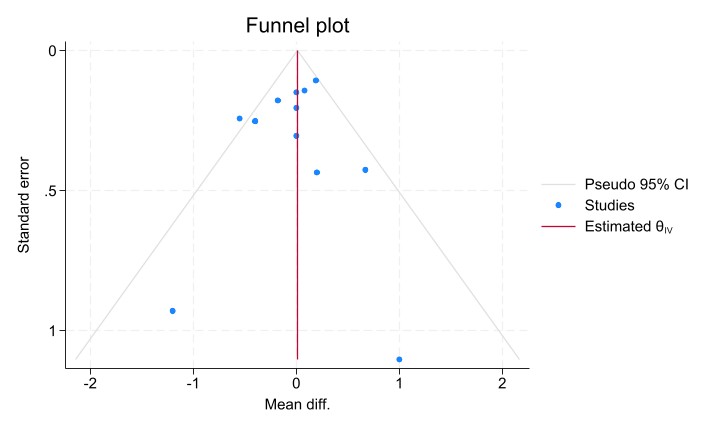


***Funnel plot analysis for publication bias in reporting mortality incidence between NIV and COT.***


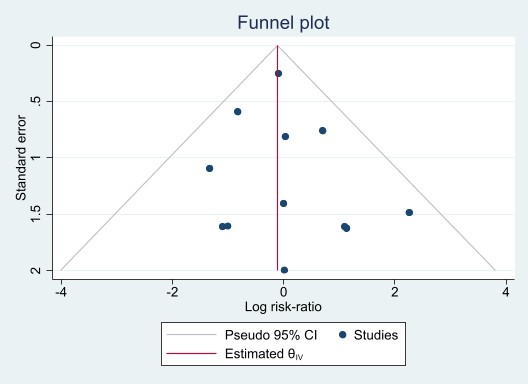


***Funnel plot analysis for publication bias in reporting PPCs incidence between NIV and COT.***


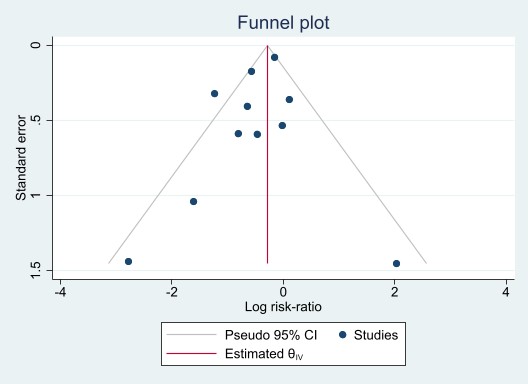


***Funnel plot analysis for publication bias in reporting pneumonia incidence between NIV and COT.***


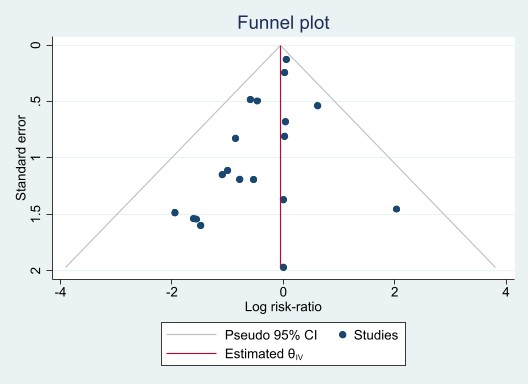


***Funnel plot analysis for publication bias in reporting reintubation rate between NIV and COT****.*


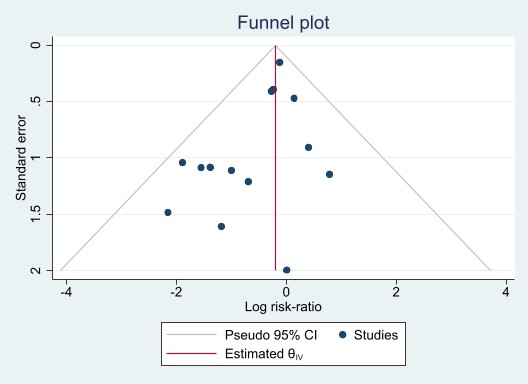


***Funnel plot analysis for publication bias in reporting ICU admission between NIV and COT.***


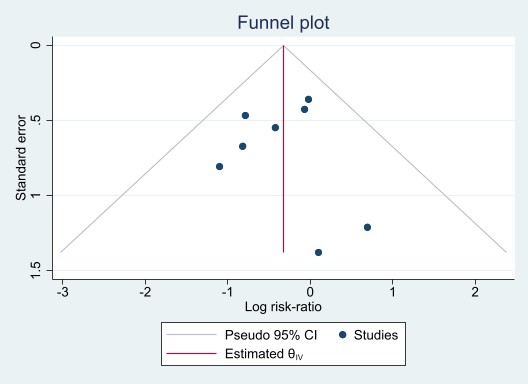


***Funnel plot analysis for publication bias in reporting length of hospital stay between NIV and COT.***


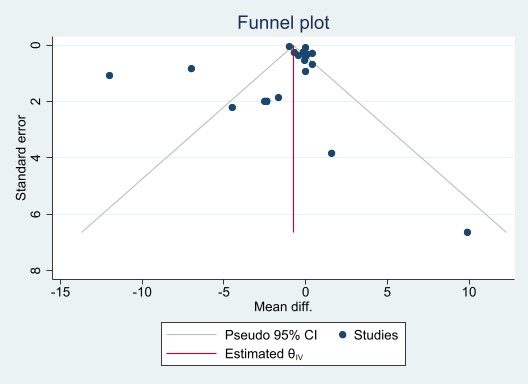

Supplement: Supplementary material 10 [file mmc10.docx]
