# Supplementary material for: Perioperative oxygen therapy: an overview of systematic reviews and meta-analyses
Source: Br J Anaesth. 2025 Jun 6;135(5):1456–76. doi: 10.1016/j.bja.2025.04.020 (PMC12597348; doi:10.1016/j.bja.2025.04.020)
Supplement: Supplementary material 11 [file mmc11.docx]

***Supplementary file 11: forest plots_high vs low FiO2***

***Effect of 80% FiO_2_ on SSI when compared to 30-35% FiO_2_ by type of surgery***


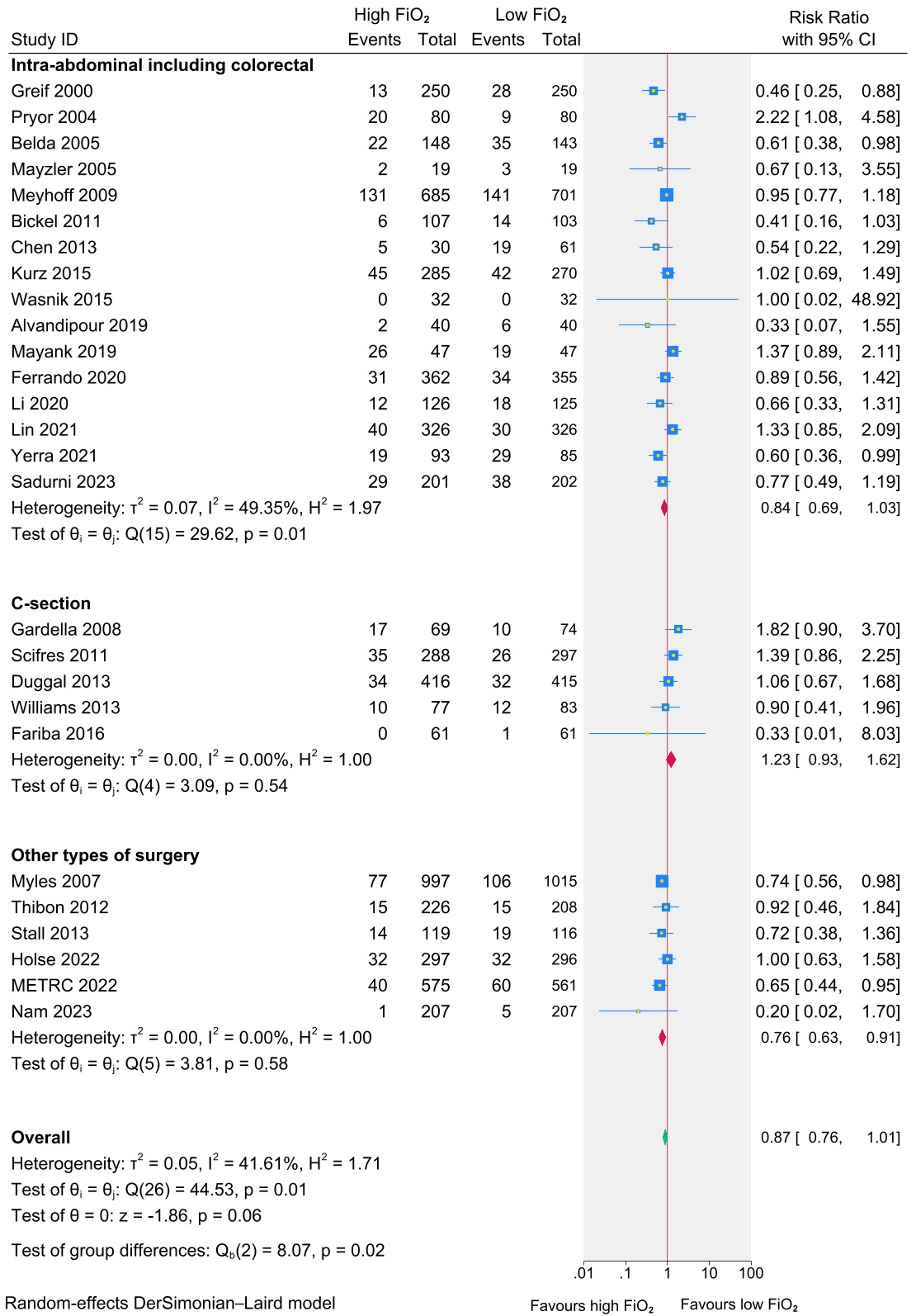


***Effect of 80% Fi****O_2_* ***on SSI when compared to low Fi****O_2_* ***by method of oxygen delivery***


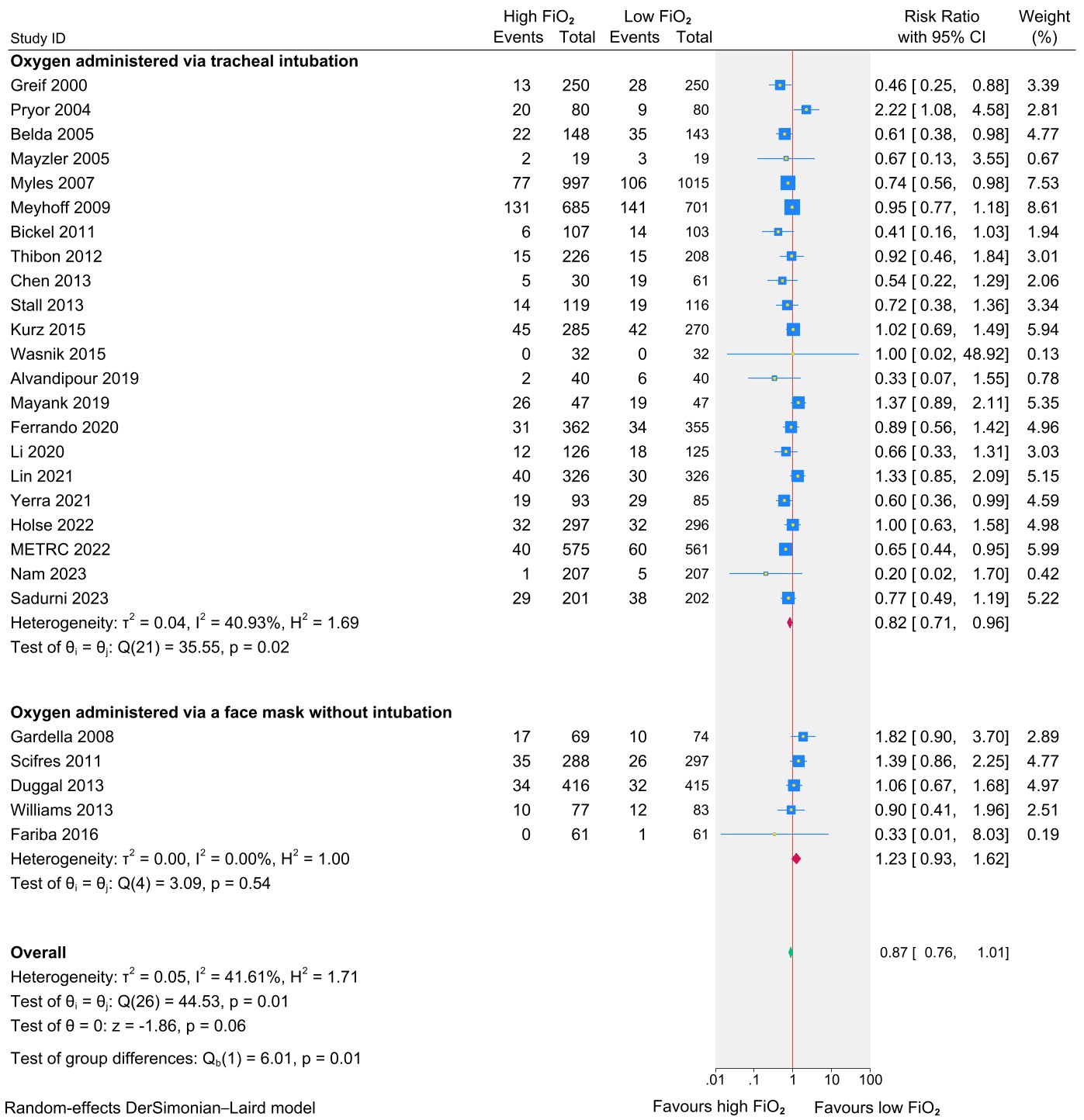


***Effect of 80% FiO_2_ on SSI when compared to 30-35% FiO_2_ according to use or no use of nitrous oxide in the control group.***


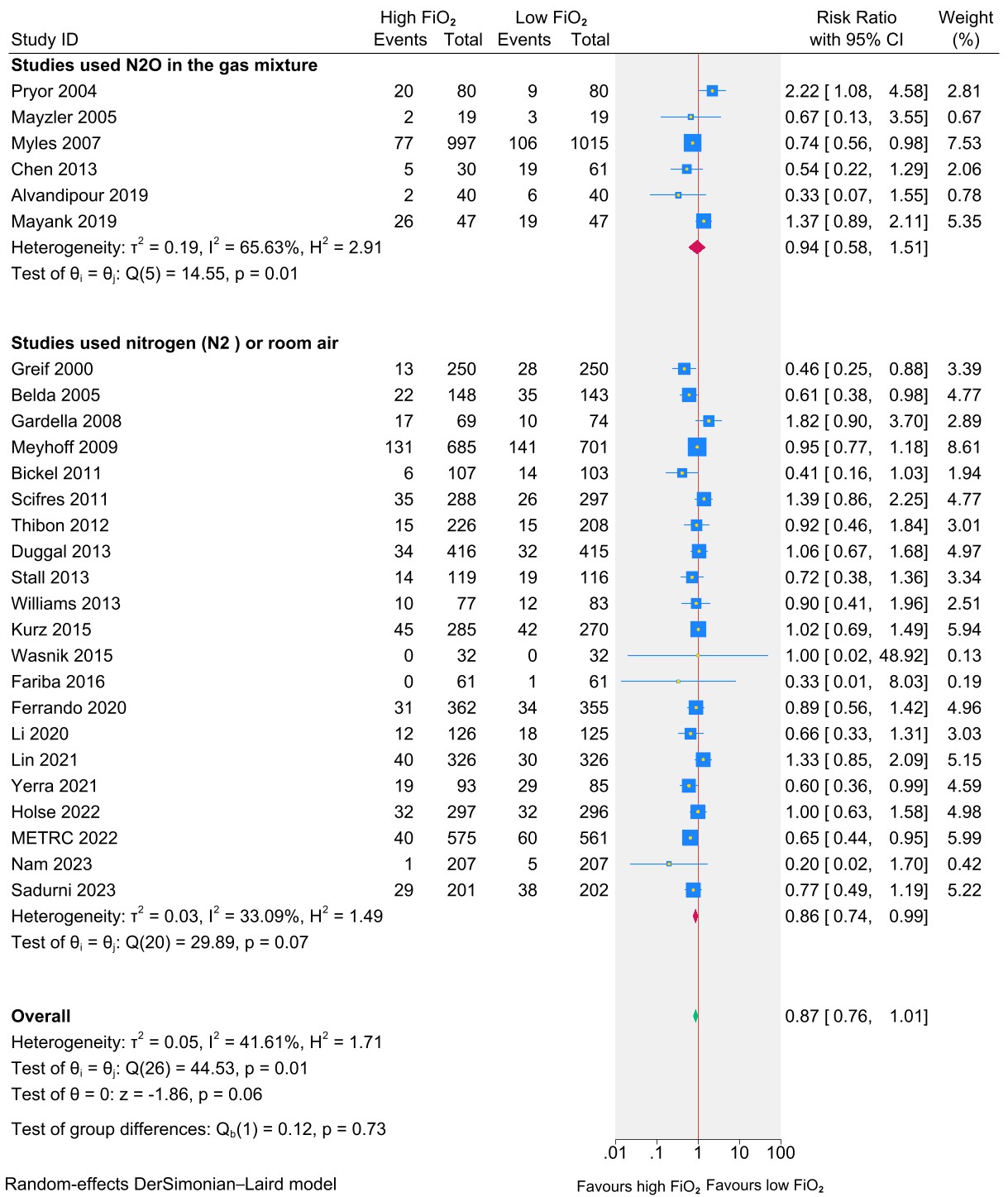


***Effect of 80% Fi****O_2_* ***on SSI when compared to 30-35% Fi****O_2_* ***by overall risk of bias.***


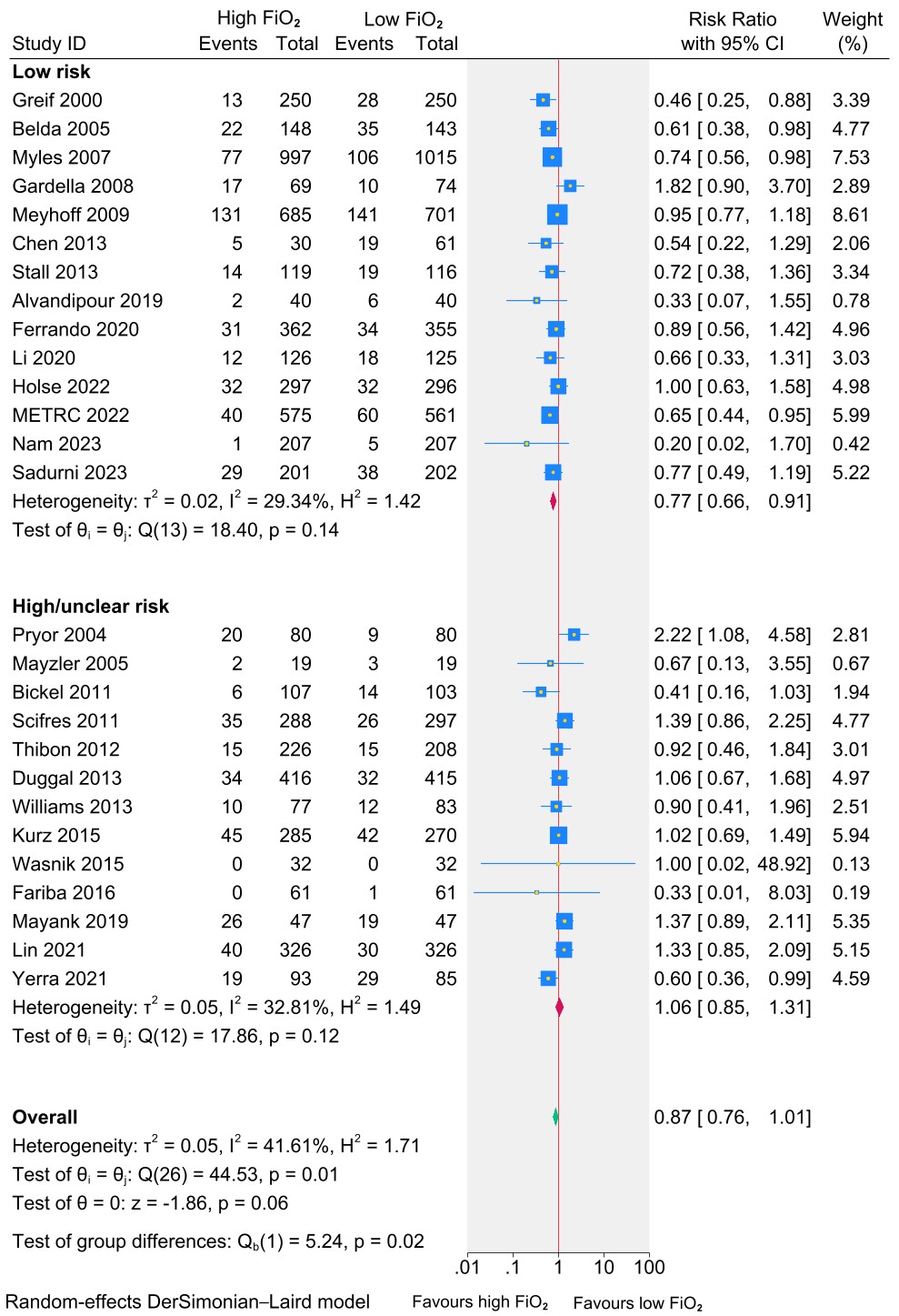


***Effect of 80% Fi****O_2_* ***on SSI when compared to 30-35% Fi****O_2_* ***by urgency of surgery.***


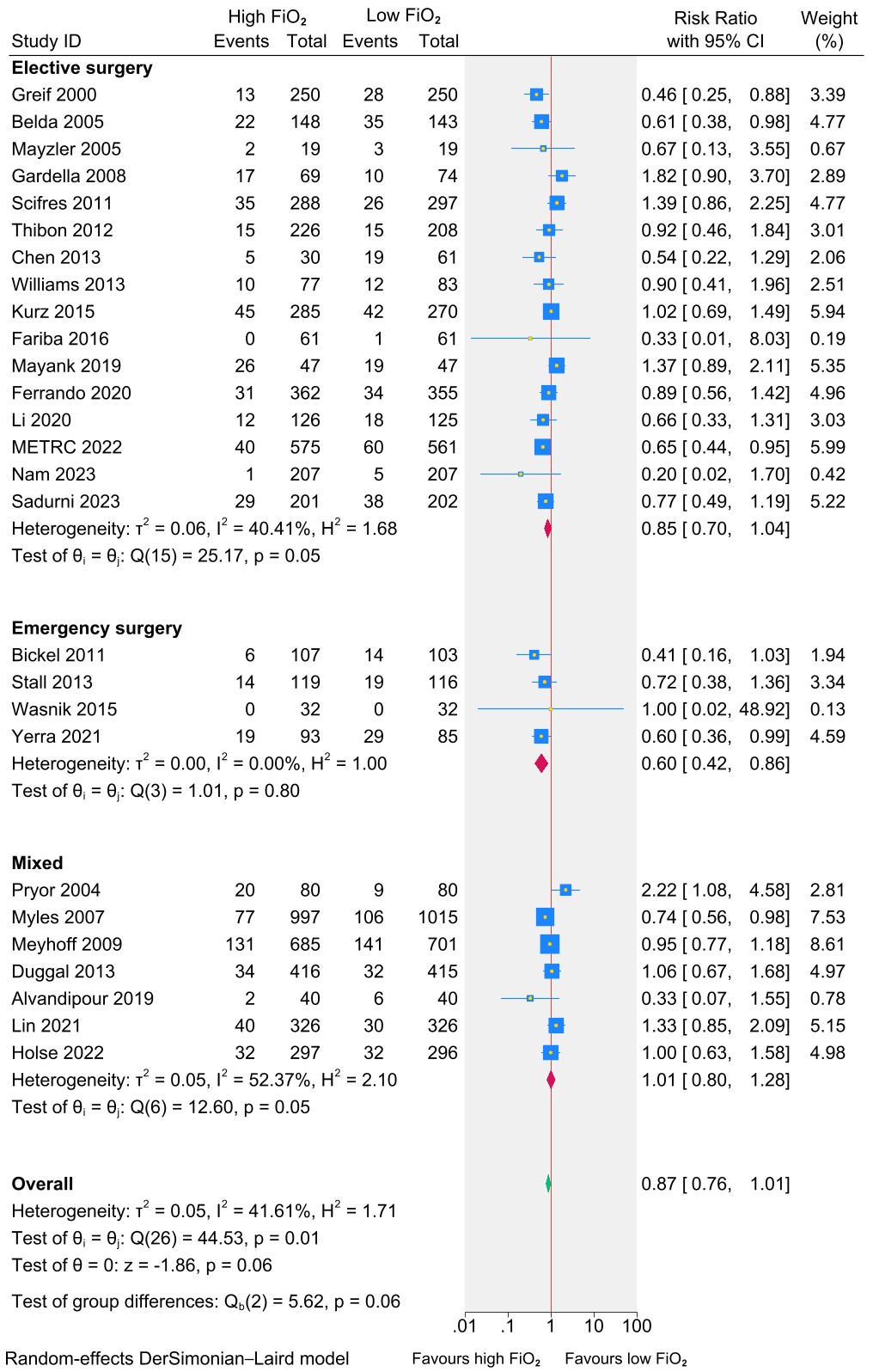


***Effect of 80% FiO2 on mortality within longest follow-up when compared to 30-35% FiO2***


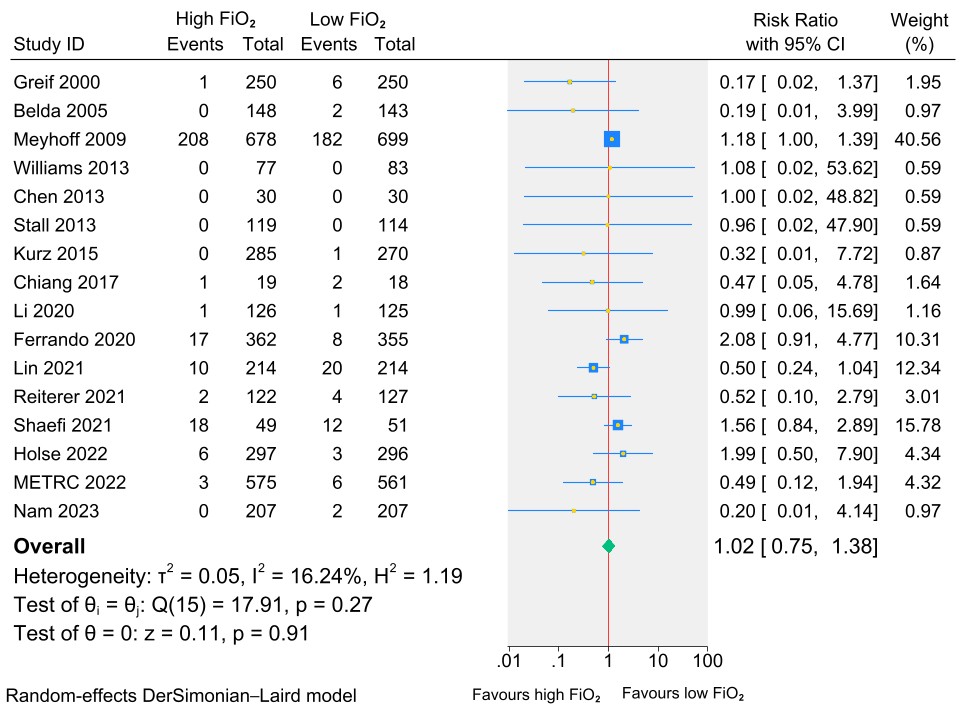


***Effect of 80% Fi****O_2_* ***on the incidence of atelectasis when compared to 30-35% Fi****O_2_*


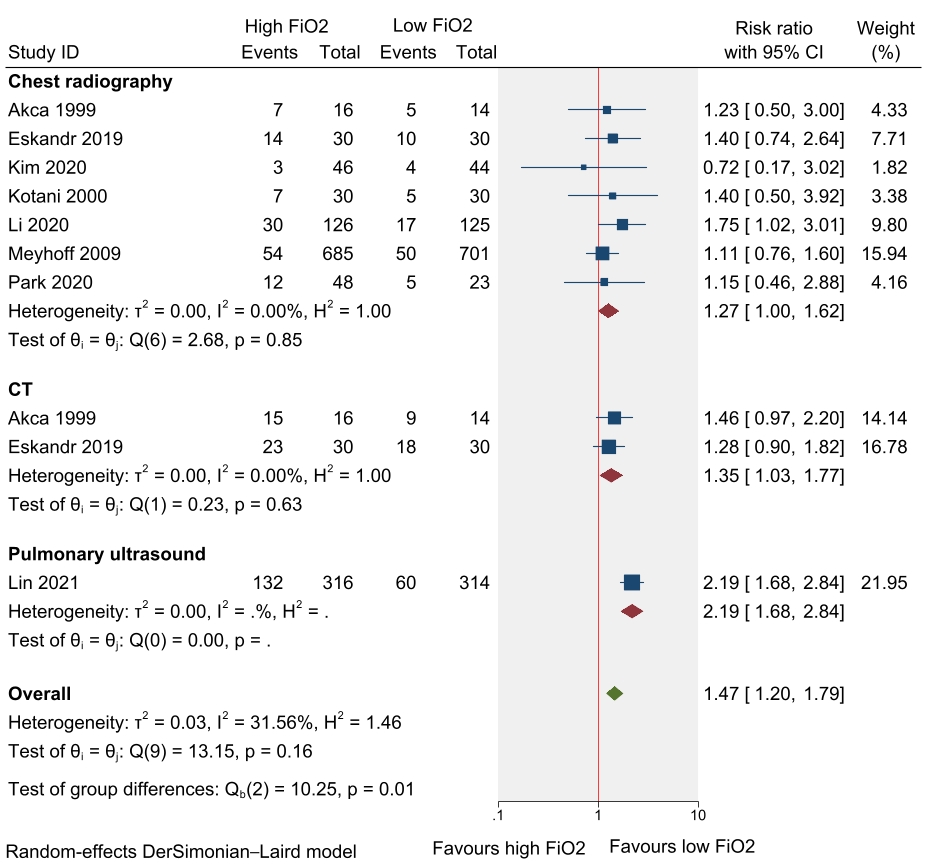


***Effect of 80% Fi****O_2_* ***on the incidence of pneumonia when compared to 30-35% Fi****O_2_*


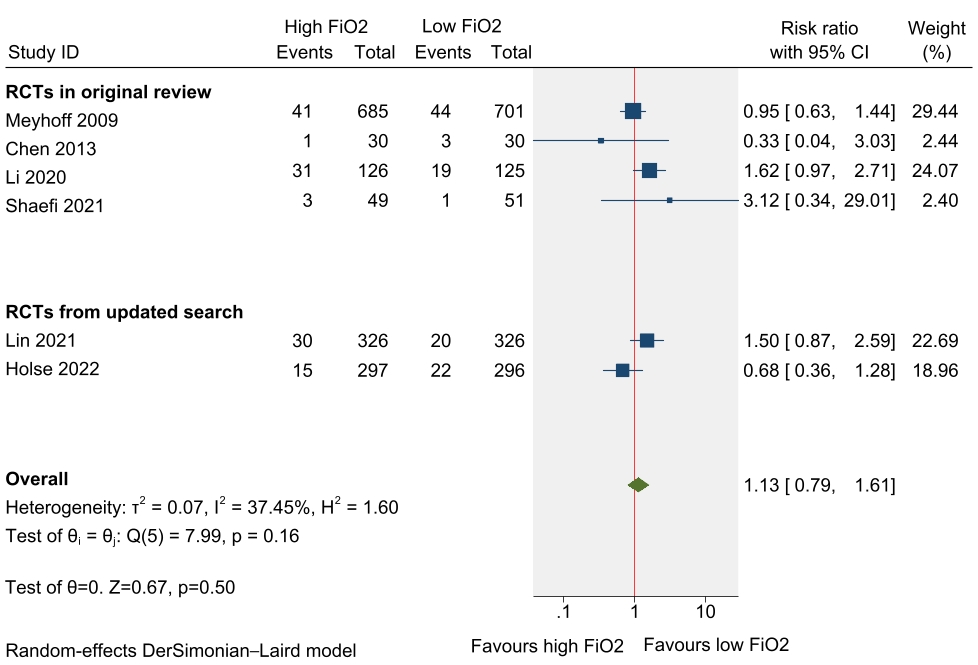


***Effect of 80% FiO_2_ on respiratory failure when compared to 30-35% FiO_2_.***


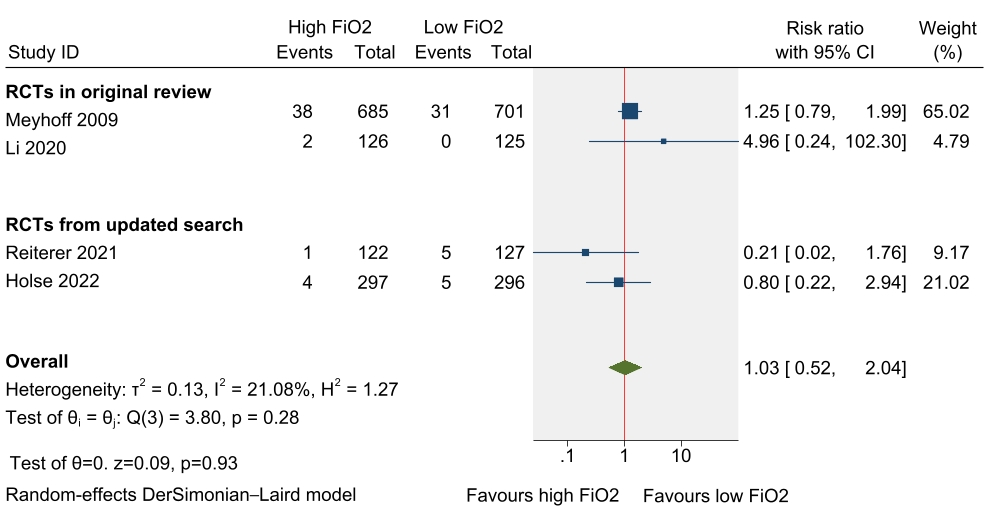


***Effect of 80% FiO_2_ on*  *postoperative pulmonary complications when compared to 30-35% FiO_2_***


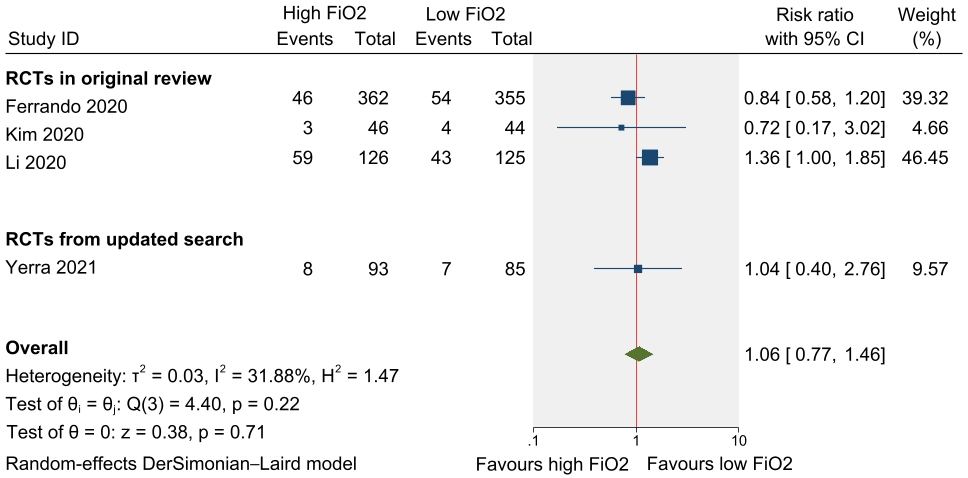


***Effect of 80% FiO_2_ on ICU admissions when compared to 30-35% FiO_2_***


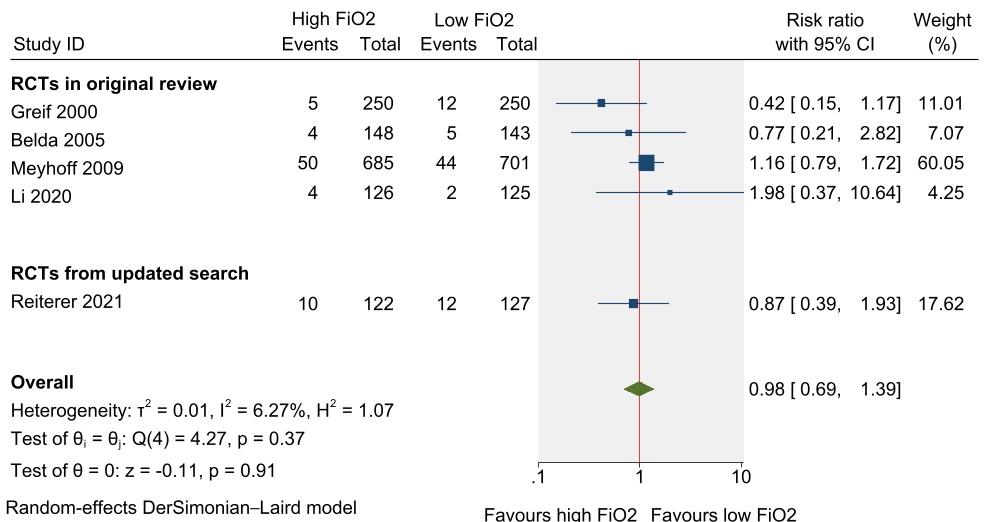


***Effect of 80% FiO_2_ on length of hospital stay when compared to 30-35% FiO_2_***


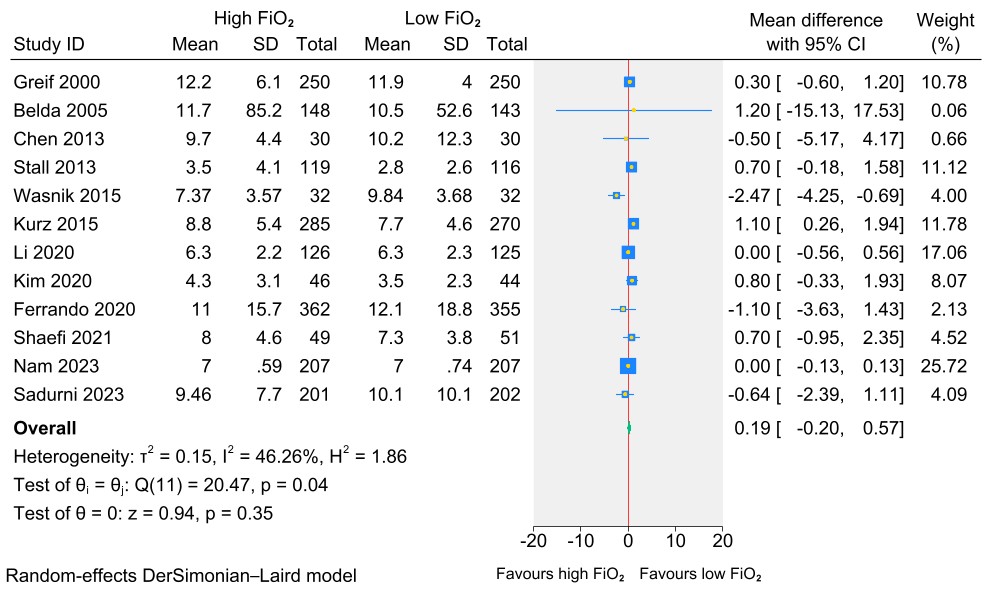


***Effect of 80% FiO2 on postoperative nausea and vomiting when compared to 30% FiO2***

**
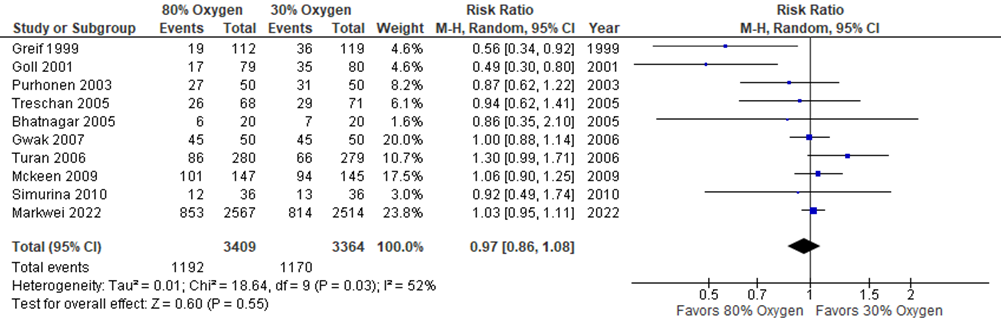
**
