# Supplementary material for: Perioperative oxygen therapy: an overview of systematic reviews and meta-analyses
Source: Br J Anaesth. 2025 Jun 6;135(5):1456–76. doi: 10.1016/j.bja.2025.04.020 (PMC12597348; doi:10.1016/j.bja.2025.04.020)
Supplement: Supplementary material 12 [file mmc12.docx]

***Supplementary file 12: Findings from meta-regression analyses exploring potential effect modifiers of high vs low FiO_2_ on surgical site infection***

| **Explanatory variable** | **No. of trials** | **Univariable**  **meta-regression** | | **Full**  **meta-regression** | |
| --- | --- | --- | --- | --- | --- |
|  |  | Ratio of risk ratio  (95% CI) | P value | Ratio of risk ratio  (95% CI) | P value |
|  | 27 | - |  | R^2^ = 40% |  |
|  |  |  |  |  |  |
| Delivery of oxygen |  | R^2^ = 23% | - |  |  |
| Intubation | 22 | 1 (Reference) |  | 1 (Reference) |  |
| No intubation | 5 | 1.48 (1.02 to 2.15) | 0.03 | 1.30 (0.89 to 1.92) | 0.17 |
|  |  |  |  |  |  |
| Type of surgery |  | R^2^ = 22% | 0.09 |  |  |
| Intra-abdominal  (including colorectal) | 16 | 1 (Reference) |  |  |  |
| C-section | 5 | 1.43 (0.97 to 2.10) | 0.06 |  |  |
| Other types of surgery | 6 | 0.88 (0.64 to 1.21) | 0.46 |  |  |
|  |  |  |  |  |  |
| Urgency of surgery |  | R^2^ = 9% | 0.10* |  |  |
| Elective surgery | 13 | 1 (Reference) |  |  |  |
| Mixed | 7 | 1.18 (0.87 to 1.59) | 0.27 |  |  |
| Emergency surgery | 4 | 0.69 (0.43 to 1.12) | 0.13 |  |  |
|  |  |  |  |  |  |
| Risk of bias |  | R^2^ = 35% | - |  |  |
| Low risk | 11 | 1 (Reference) |  | 1 (Reference) |  |
| High/unclear risk | 13 | 1.37 (1.06 to 1.79) | 0.01 | 1.28 (0.94 to 1.69) | 0.07 |
|  |  |  |  |  |  |
| Gas mixture |  | R^2^ = 0% | - |  |  |
| With N_2_O | 6 | 1 (Reference) |  |  |  |
| Without N_2_O | 18 | 0.88 (0.61 to 1.28) | 0.53 |  |  |
