# Supplementary material for: Perioperative oxygen therapy: an overview of systematic reviews and meta-analyses
Source: Br J Anaesth. 2025 Jun 6;135(5):1456–76. doi: 10.1016/j.bja.2025.04.020 (PMC12597348; doi:10.1016/j.bja.2025.04.020)
Supplement: Supplementary material 13 [file mmc13.docx]

***Supplementary file 13: Sensitivity analysis including Kurz 2018***

| **No. of   studies** | | **No. of SSI in the  intervention  group** | **No. of SSI in  the control group** | **Relative risk  (95% CI)** | **Univariable meta-regression (*P*-value)** | **Between study variance (I^2^)** | **Percent variance explained**  **R-squared (%)** |
| --- | --- | --- | --- | --- | --- | --- | --- |
| **Overall** | | | | | | | |
| All | 28 | 973 of 9059 (10.7%) | 1097 of 9025 (12.1%) | 0.88 (0.78 – 1.00) | NA | 39% | NA |
| **Delivery of oxygen** | | | | | | | |
| Intubation | 23 | 877 of 8148 (10.7%) | 1016 of 8095 (12.5%) | 0.84 (0.74 - 0.96) | 0.03 | 34% | 21% |
| No intubation | 5 | 96 of 911 (10.5%) | 81 of 930 (8.7%) | 1.23 (0.93–1.62) |  |  |  |
| **Type of surgery** | | | | | | | |
| Intra-abdominal including colorectal | 17 | 698 of 5727 (12.2%) | 779 of 5692 (13.7%) | 0.86 (0.73 – 1.02) | 0.06 | 32% | 25% |
| C-section | 5 | 96/911 (10.5%) | 81/930 (8.7%) | 1.23 (0.93–1.62) |  |  |  |
| Other types of surgery | 6 | 179 of 2421 (7.4%) | 237 of 2403 (9.8%) | 0.76 (0.63-0.91) |  |  |  |
| **Urgency of surgery** | | | | | | | |
| Elective surgery | 16 | 303 of 2971 (10.2%) | 365 of 2963 (12.3%) | 0.85 (0.70-1.04) | 0.09 | 36% | 11% |
| Emergency surgery | 4 | 39 of 351 (11.1%) | 62 of 336 (18.4%) | 0.60 (0.42-0.86) |  |  |  |
| Mixed | 8 | 631 of 5737 (11%) | 670 of 5726 (11.7%) | 0.97 (0.82-1.15) |  |  |  |
| **Risk of bias** | | | | | | | |
| Low risk | 14 | 426 of 4106 (10.4%) | 551 of 4146 (13.3%) | 0.77 (0.66-0.91) | 0.01 | 31% | 22% |
| High/unclear risk | 14 | 547/4953 (11%) | 546/4879 (11.2%) | 1.03 (0.86–1.23) |  |  |  |
| **Gas mixture** | | | | | | | |
| With N_2_O | 6 | 132/1213 (10.9%) | 162/1262 (12.8%) | 0.94 (0.58–1.51) | 0.55 | 42% | - |
| Without N_2_O | 22 | 841 of 7846 (10.7%) | 935 of 7763 (12%) | 0.87 (0.77 – 0.99) |  |  |  |
