# Supplementary material for: Perioperative oxygen therapy: an overview of systematic reviews and meta-analyses
Source: Br J Anaesth. 2025 Jun 6;135(5):1456–76. doi: 10.1016/j.bja.2025.04.020 (PMC12597348; doi:10.1016/j.bja.2025.04.020)
Supplement: Supplementary material 14 [file mmc14.docx]

***Supplementary file 14. GRADE evidence profiles and summary of findings***

***High vs low FiO2***

| **Certainty assessment** | | | | | | | **Summary of findings** | | | | |
| --- | --- | --- | --- | --- | --- | --- | --- | --- | --- | --- | --- |
| **Participants (studies) Follow-up** | **Risk of bias** | **Inconsistency** | **Indirectness** | **Imprecision** | **Publication bias** | **Overall certainty of evidence** | **Study event rates (%)** | | **Relative effect (95% CI)** | **Anticipated absolute effects** | |
|  |  |  |  |  |  |  | **With Low FiO2** | **With High FiO2** |  | **Risk with Low FiO2** | **Risk difference with High FiO2** |
| **Surgical site infection** | | | | | | | | | | | |
| 12335 (27 RCTs) | not serious | serious^a^ | not serious | Very serious^b^ | none | ⨁◯◯◯ Very low | 783/6172 (12.7%) | 678/6163 (11.0%) | **RR 0.87** (0.76 to 1.01) | 127 per 1,000 | **16 fewer per 1,000** (from 30 fewer to 1 more) |
| **Mortality within 30 days** | | | | | | | | | | | |
| 5116 (11 RCTs) | not serious | not serious | not serious | serious^b^ | publication bias strongly suspected^c^ | ⨁⨁◯◯ Low | 44/2555 (1.7%) | 47/2561 (1.8%) | **RR 1.17** (0.77 to 1.78) | 17 per 1,000 | **3 more per 1,000** (from 4 fewer to 13 more) |
| **Mortality within longest follow-up** | | | | | | | | | | | |
| 7101 (16 RCTs) | not serious | not serious | not serious | extremely serious^b^ | none | ⨁◯◯◯ Very low | 249/3543 (7%) | 267/3558 (7.5%) | **RR 1.02** (0.75 to 1.38) | 70 per 1,000 | **1 more per 1,000** (from 18 fewer to 27 more) |
| **Incidence of atelectasis** | | | | | | | | | | | |
| 2668 (10 RCTs) | not serious | serious^e^ | not serious | serious^b^ | none | ⨁⨁◯◯ Low | 183/1325 (13.8%) | 297/1343 (22.1%) | **RR 1.47** (1.20 to 1.79) | 138 per 1,000 | **65 more per 1,000** (from 28 more to 109 more) |
| **Pneumonia** | | | | | | | | | | | |
| 3042 (6 RCTs) | not serious | not serious | not serious | extremely serious^b^ | none | ⨁◯◯◯ Very low | 109/1529 (7.1%) | 121/1513 (8.0%) | **RR 1.13** (0.79 to 1.61) | 71 per 1,000 | **9 more per 1,000** (from 15 fewer to 43 more) |
| **Postoperative pulmonary complications (PPCs)** | | | | | | | | | | | |
| 1236 (4 RCTs) | not serious | not serious | not serious | extremely serious^b^ | none | ⨁◯◯◯ Very low | 108/609 (17.7%) | 116/627 (18.5%) | **RR 1.06** (0.77 to 1.46) | 177 per 1,000 | **11 more per 1,000** (from 41 fewer to 82 more) |
| **Respiratory failure** | | | | | | | | | | | |
| 2479 (4 RCTs) | not serious | not serious | not serious | extremely serious^b^ | none | ⨁◯◯◯ Very low | 41/1249 (3.3%) | 45/1230 (3.7%) | **RR 1.03** (0.52 to 2.04) | 33 per 1,000 | **1 more per 1,000** (from 16 fewer to 34 more) |
| **ICU admission** | | | | | | | | | | | |
| 2677 (5 RCTs) | not serious | not serious | not serious | very serious^b^ | none | ⨁⨁◯◯ Low | 75/1346 (5.6%) | 73/1331 (5.5%) | **RR 0.98** (0.69 to 1.39) | 56 per 1,000 | **1 fewer per 1,000** (from 17 fewer to 22 more) |
| **Length of hospital stay** | | | | | | | | | | | |
| 3680 (12 RCTs) | not serious | serious | not serious | serious^b^ | none | ⨁⨁◯◯ Low | 1825 | 1855 | - |  | MD **0.19 higher** (0.2 lower to 0.57 higher) |

**CI:** confidence interval; **MD:** mean difference; **RR:** risk ratio

**Explanations for downgrading**

a. Statistically significant heterogeneity.

b. Wide confidence interval suggesting appreciable benefit and appreciable harm.

c. Egger’s test for small-study effects was statistically significant.

**GRADE Working Group grades of evidence**
**High certainty:** we are very confident that the true effect lies close to that of the estimate of the effect
**Moderate certainty:** we are moderately confident in the effect estimate: the true effect is likely to be close to the estimate of the effect, but there is a possibility that it is substantially different
**Low certainty:** our confidence in the effect estimate is limited: The true effect may be substantially different from the estimate of the effect
**Very low certainty:** we have very little confidence in the effect estimate: The true effect is likely to be substantially different from the estimate of effect

***NIV vs COT***

| **Certainty assessment** | | | | | | | **Summary of findings** | | | | |
| --- | --- | --- | --- | --- | --- | --- | --- | --- | --- | --- | --- |
| **Participants (studies) Follow-up** | **Risk of bias** | **Inconsistency** | **Indirectness** | **Imprecision** | **Publication bias** | **Overall certainty of evidence** | **Study event rates (%)** | | **Relative effect (95% CI)** | **Anticipated absolute effects** | |
|  |  |  |  |  |  |  | **With COT** | **With NIV** |  | **Risk with COT** | **Risk difference with NIV** |
| **Mortality** | | | | | | | | | | | |
| 7876 (19 RCTs) | not serious | not serious | not serious | not serious | none | ⨁⨁⨁⨁ High | 54/3704 (1.5%) | 57/4172 (1.4%) | **RR 0.91** (0.62 to 1.32) | 15 per 1,000 | **1 fewer per 1,000** (from 6 fewer to 5 more) |
| **Pneumonia** | | | | | | | | | | | |
| 7848 (19 RCTs) | not serious | not serious | not serious | serious^a^ | publication bias strongly suspected^b^ | ⨁⨁◯◯ Low | 202/3691 (5.5%) | 202/4157 (4.9%) | **RR 0.95** (0.78 to 1.15) | 55 per 1,000 | **3 fewer per 1,000** (from 12 fewer to 8 more) |
| **PPCs** | | | | | | | | | | | |
| 2142 (10 RCTs) | not serious | serious^c^ | not serious | serious^a^ | none | ⨁⨁◯◯ Low | 266/828 (32.1%) | 379/1314 (28.8%) | **RR 0.62** (0.44 to 0.87) | 321 per 1,000 | **122 fewer per 1,000** (from 180 fewer to 42 fewer) |
| **Reintubation** | | | | | | | | | | | |
| 7782 (17 RCTs) | not serious | not serious | not serious | serious^a^ | none | ⨁⨁⨁◯ Moderate | 149/3657 (4.1%) | 130/4125 (3.2%) | **RR 0.82** (0.65 to 1.04) | 41 per 1,000 | **7 fewer per 1,000** (from 14 fewer to 2 more) |
| **Unplanned ICU admission** | | | | | | | | | | | |
| 2382 (9 RCTs) | not serious | not serious | not serious | very serious^a^ | none | ⨁⨁◯◯ Low | 57/958 (5.9%) | 58/1424 (4.1%) | **RR 0.73** (0.50 to 1.06) | 59 per 1,000 | **16 fewer per 1,000** (from 30 fewer to 4 more) |
| **ARDS** | | | | | | | | | | | |
| 6617 (8 RCTs) | not serious | not serious | not serious | serious^a^ | none | ⨁⨁⨁◯ Moderate | 123/3073 (4.0%) | 98/3544 (2.8%) | **RR 0.70** (0.53 to 0.93) | 40 per 1,000 | **12 fewer per 1,000** (from 19 fewer to 3 fewer) |
| **Pulmonary aspiration** | | | | | | | | | | | |
| 6041 (4 RCTs) | not serious | not serious | not serious | not serious | none | ⨁⨁⨁⨁ High | 13/2784 (0.5%) | 15/3257 (0.5%) | **RR 1.11** (0.54 to 2.27) | 5 per 1,000 | **1 more per 1,000** (from 2 fewer to 6 more) |
| **Length of hospital stay** | | | | | | | | | | | |
| 8157 (19 RCTs) | not serious | serious^c^ | not serious | not serious | none | ⨁⨁⨁◯ Moderate | 3845 | 4312 | - | - | MD **1.12 days fewer** (1.69 fewer to 0.55 fewer) |

**CI:** confidence interval; **RR:** risk ratio; **SMD:** standardised mean difference

**Explanations for downgrading**

a. Wide confidence interval suggesting appreciable benefit and appreciable harm.

b. Egger’s test for small-study effects was statistically significant.

c. Statistically significant heterogeneity

**GRADE Working Group grades of evidence**
**High certainty:** we are very confident that the true effect lies close to that of the estimate of the effect
**Moderate certainty:** we are moderately confident in the effect estimate: the true effect is likely to be close to the estimate of the effect, but there is a possibility that it is substantially different
**Low certainty:** our confidence in the effect estimate is limited: The true effect may be substantially different from the estimate of the effect
**Very low certainty:** we have very little confidence in the effect estimate: The true effect is likely to be substantially different from the estimate of effect

***HFNO vs COT***

| **Certainty assessment** | | | | | | | **Summary of findings** | | | | |
| --- | --- | --- | --- | --- | --- | --- | --- | --- | --- | --- | --- |
| **Participants (studies)**  (studies) Follow-up | **Risk of bias** k of bias | Inco **Inconsistency** n | Ind **Indirectness** ir | **Imprecision** | **Publication bias** | **Overall certainty of evidence** | **Study event rates (%)** | | **Relative effect (95% CI)** | **Anticipated absolute effects** | |
|  |  |  |  |  |  |  | **With COT** | **With HFNO** |  | **Risk with COT** | **Risk difference with HFNO** |
| **Mortality** | | | | | | | | | | | |
| 853 (5 RCTs) | not serious | not serious | not serious | very serious^a^ | none | ⨁⨁◯◯ Low | 8/411 (1.9%) | 7/442 (1.6%) | **RR 0.78** (0.27 to 2.24) | 19 per 1,000 | **4 fewer per 1,000** (from 14 fewer to 24 more) |
| **Reintubation** | | | | | | | | | | | |
| 1156 (9 RCTs) | not serious | not serious | not serious | extremely serious^a^ | none | ⨁◯◯◯ Very low | 23/555 (4.1%) | 21/601 (3.5%) | **RR 0.78** (0.29 to 2.07) | 41 per 1,000 | **9 fewer per 1,000** (from 29 fewer to 44 more) |
| **Escalation of respiratory support** | | | | | | | | | | | |
| 1389 (10 RCTs) | not serious | serious^b^ | serious^c^ | Very serious^a^ | none | ⨁◯◯◯ Very low | 131/674 (19.4%) | 106/715 (14.8%) | **RR 0.59** (0.40 to 0.88) | 194 per 1,000 | **80 fewer per 1,000** (from 117 fewer to 23 fewer) |
| **Hospital LOS** | | | | | | | | | | | |
| 1447 (13 RCTs) | not serious | serious^b^ | not serious | not serious | none | ⨁⨁⨁◯  Moderate | 732 | 715 | - |  | MD **0.63 lower** (1.1 lower to 0.16 lower) |
| **ICU LOS** | | | | | | | | | | | |
| 1421 (12 RCTs) | not serious | not serious | not serious | serious^a^ | none | ⨁⨁⨁◯ Moderate | 710 | 711 | - |  | MD **0.03 higher** (0.19 lower to 0.14 higher) |
| **Postoperative hypoxemia** | | | | | | | | | | | |
| 829 (5 RCTs) | not serious | serious^b^ | not serious | extremely serious^a^ | none | ⨁◯◯◯ Very low | 207/417 (49.6%) | 174/412 (42.2%) | **RR 0.79** (0.58 to 1.06) | 496 per 1,000 | **104 fewer per 1,000** (from 208 fewer to 30 more) |

**CI:** confidence interval; **MD:** mean difference; **RR:** risk ratio

**Explanations for downgrading**

a. Wide confidence interval.

b. Statistically significant heterogeneity.

c. Escalating therapy has various degrees of importance, for example, escalating to NIV is less predictive of poorer outcomes than escalating to invasive mechanical ventilation. Also, some patients in the control group escalated to HFNO.

**GRADE Working Group grades of evidence**
**High certainty:** we are very confident that the true effect lies close to that of the estimate of the effect
**Moderate certainty:** we are moderately confident in the effect estimate: the true effect is likely to be close to the estimate of the effect, but there is a possibility that it is substantially different
**Low certainty:** our confidence in the effect estimate is limited: The true effect may be substantially different from the estimate of the effect
**Very low certainty:** we have very little confidence in the effect estimate: The true effect is likely to be substantially different from the estimate of effect
