# Supplementary material for: Perioperative oxygen therapy: an overview of systematic reviews and meta-analyses
Source: Br J Anaesth. 2025 Jun 6;135(5):1456–76. doi: 10.1016/j.bja.2025.04.020 (PMC12597348; doi:10.1016/j.bja.2025.04.020)

***Supplementary file 15: forest plots_ NIV vs COT***

**Effect of NIV on mortality when compared to conventional oxygen therapy. Studies are grouped by type of surgery.**

***
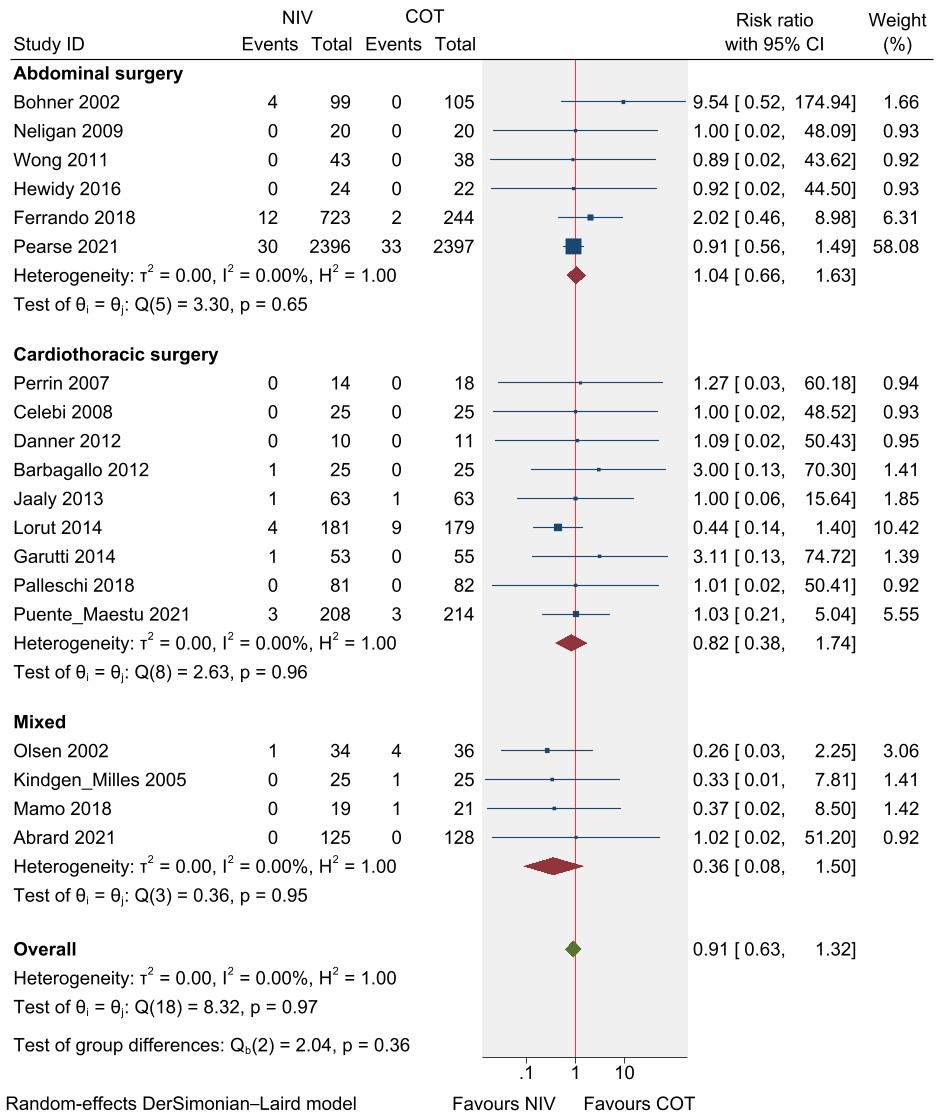
***

***Effect of NIV on PPCs when compared to conventional oxygen therapy.***


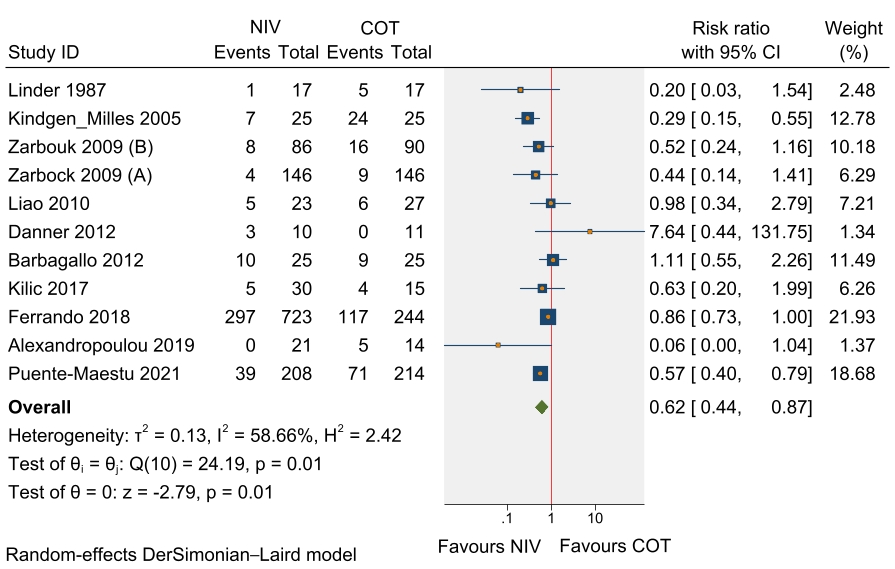


***Effect of NIV on pneumonia when compared to conventional oxygen therapy***


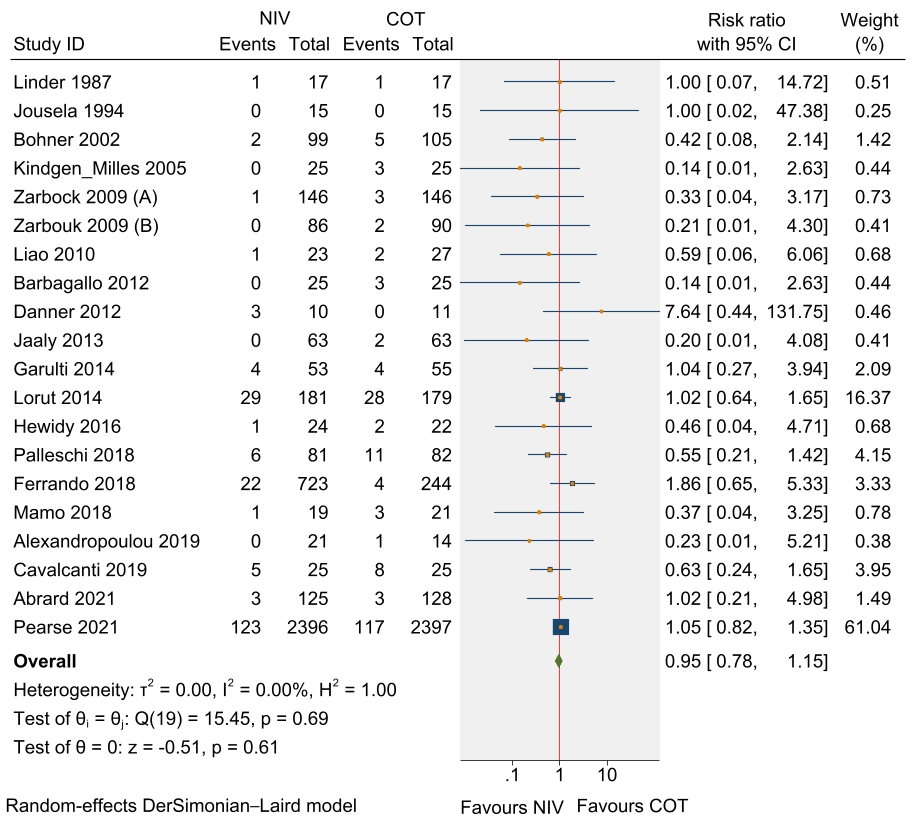


**Effect of NIV on pneumonia when compared to conventional oxygen therapy. Studies are grouped by type of surgery.**


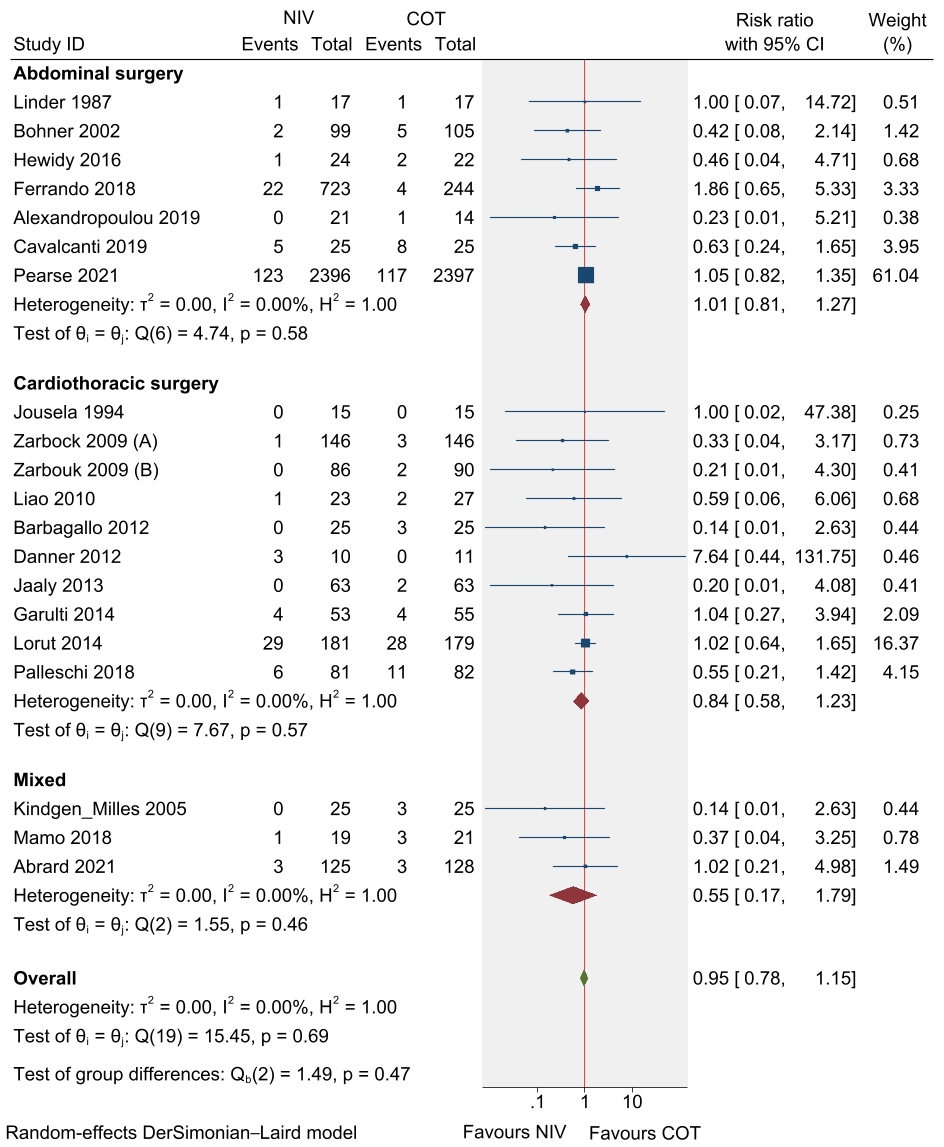


***Effect of NIV on reintubation when compared to conventional oxygen therapy.***


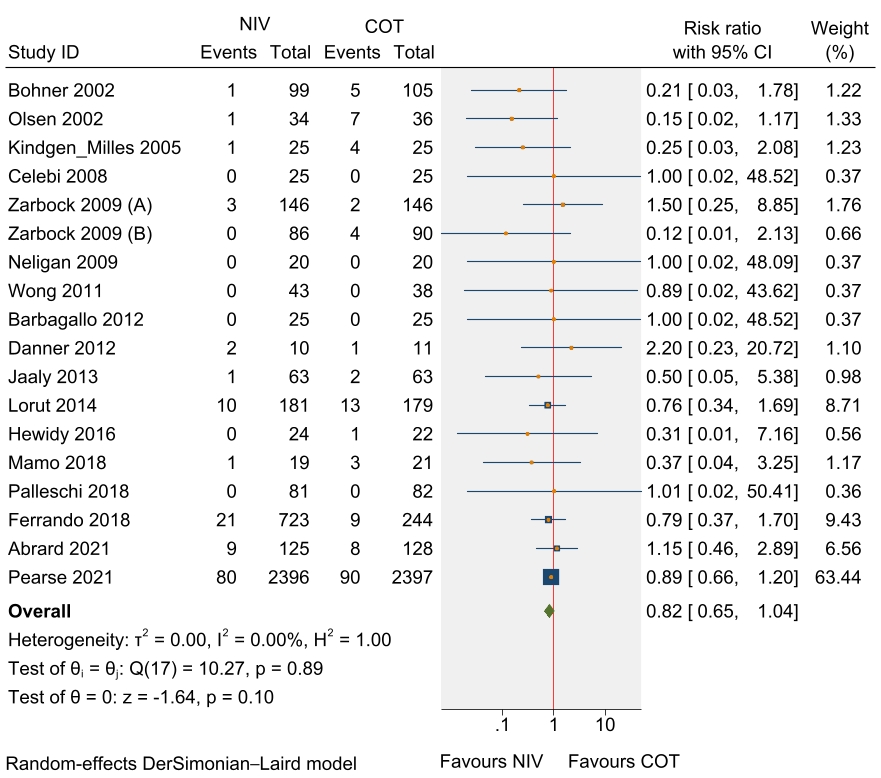


***Effect of NIV on unplanned ICU admission when compared to conventional oxygen therapy.***


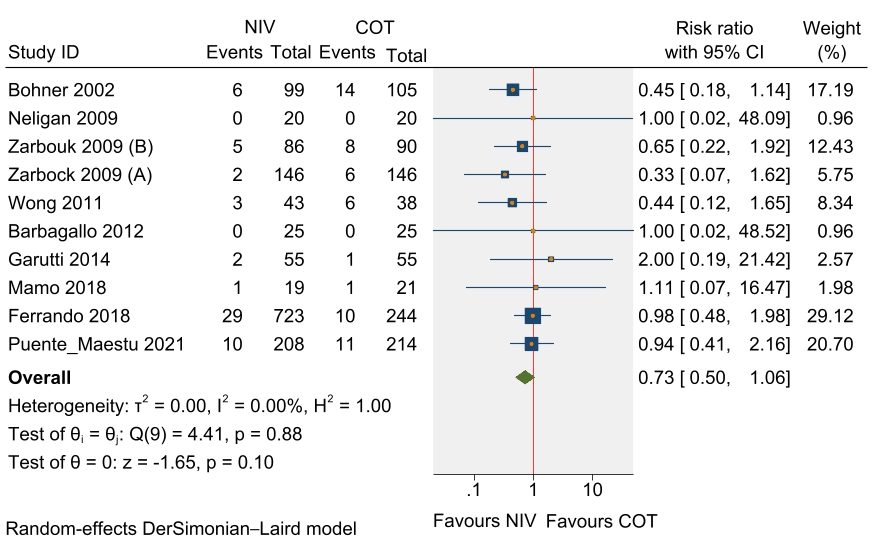


***Effect of NIV on ARDS when compared to conventional oxygen therapy.***


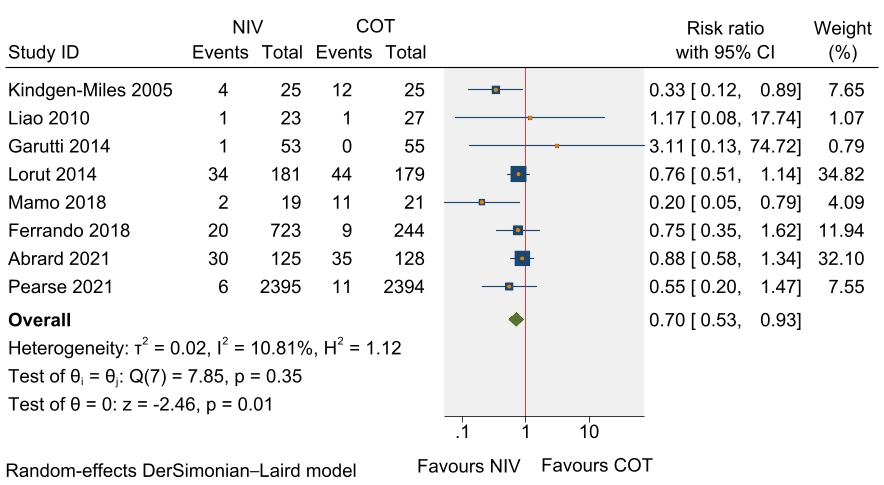


***Effect of NIV on pulmonary aspiration when compared to conventional oxygen therapy.***


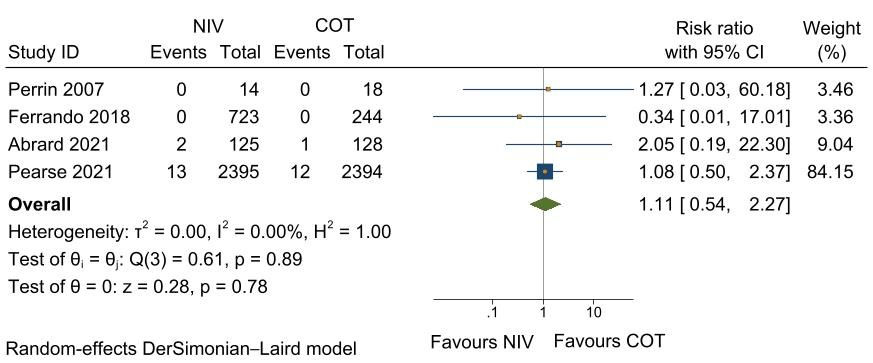


***Effect of NIV on length of hospital stay when compared to conventional oxygen therapy.***


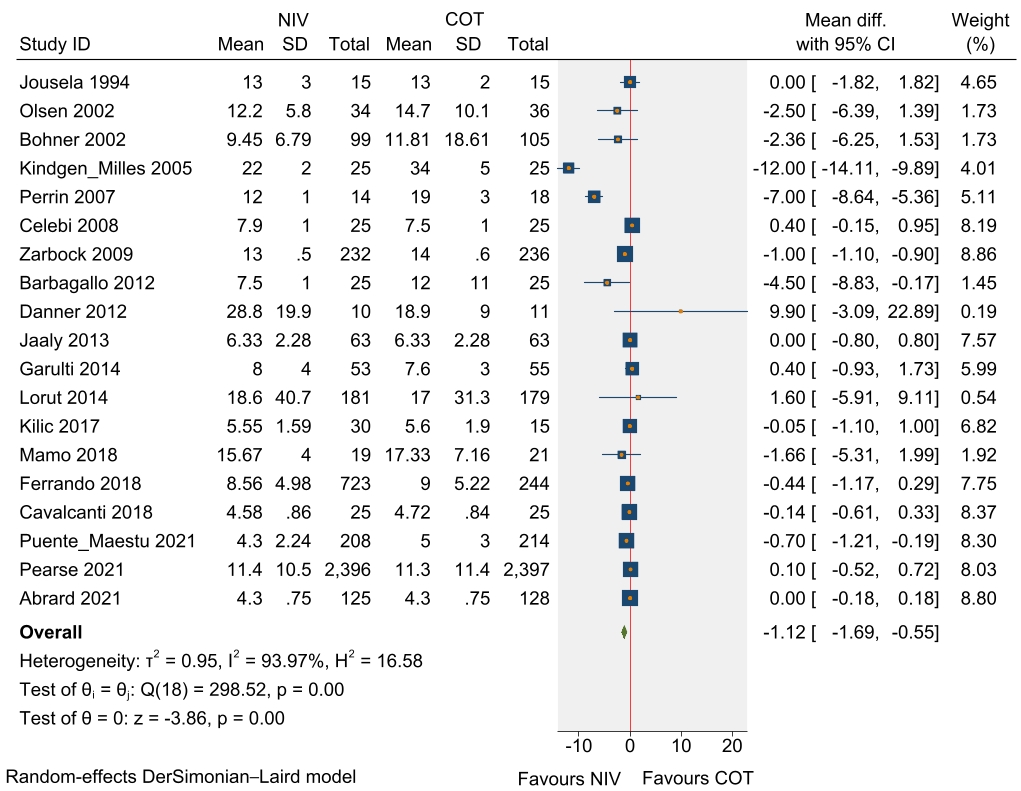

Supplement: Supplementary material 15 [file mmc15.docx]
