# Supplementary material for: Perioperative oxygen therapy: an overview of systematic reviews and meta-analyses
Source: Br J Anaesth. 2025 Jun 6;135(5):1456–76. doi: 10.1016/j.bja.2025.04.020 (PMC12597348; doi:10.1016/j.bja.2025.04.020)

***Supplementary file 16: forest plots_HFNO vs COT***

***Effect of HFNO on hospital length of stay when compared to conventional oxygen therapy***


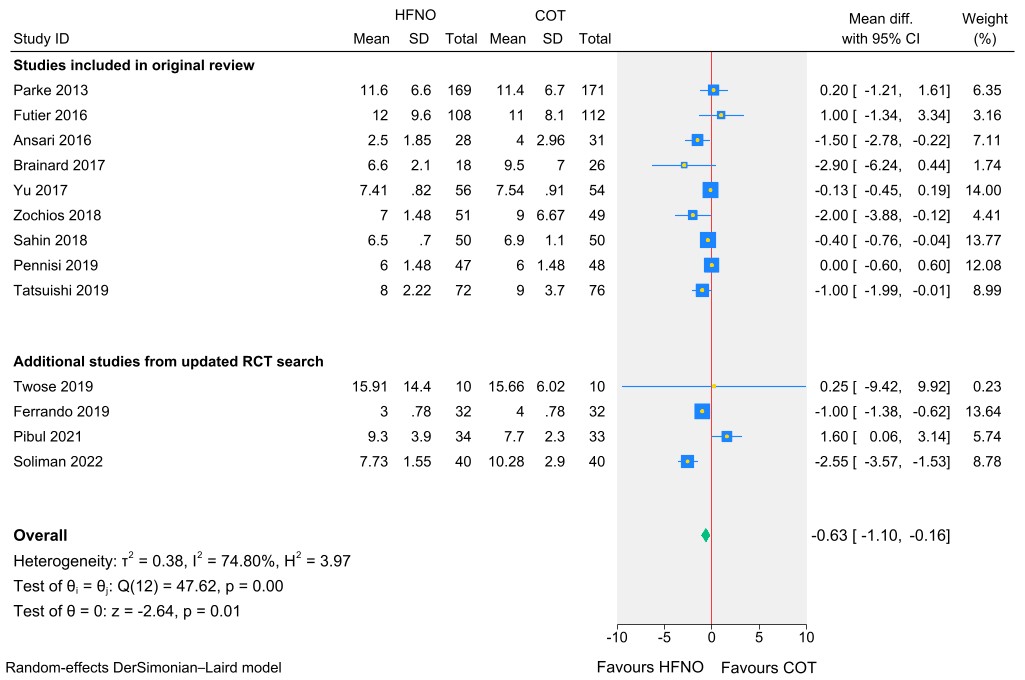


***Effect of HFNO on reintubation when compared to conventional oxygen therapy.***


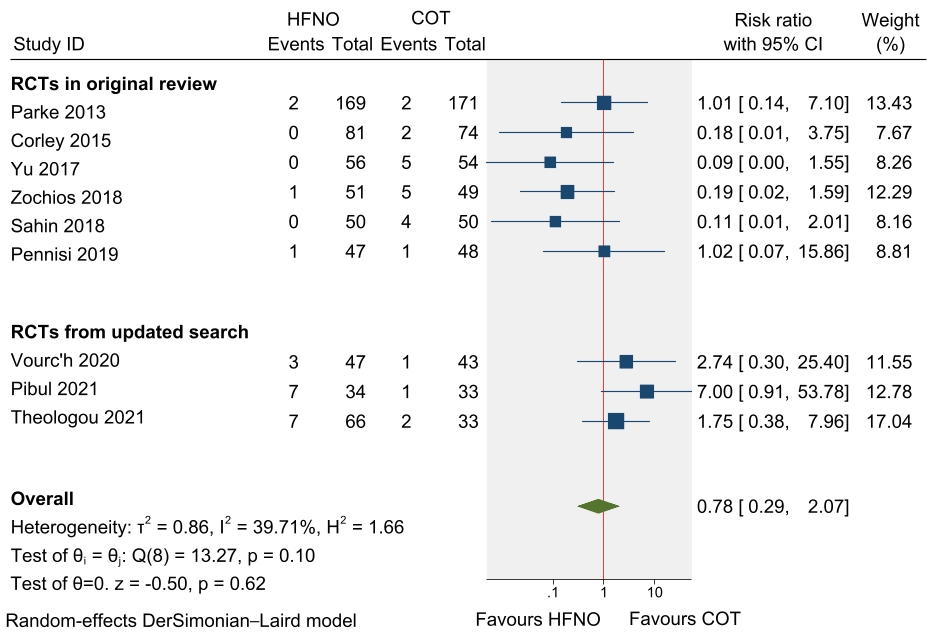


***Effect of HFNO on the need for escalation of respiratory support when compared to conventional oxygen therapy.***


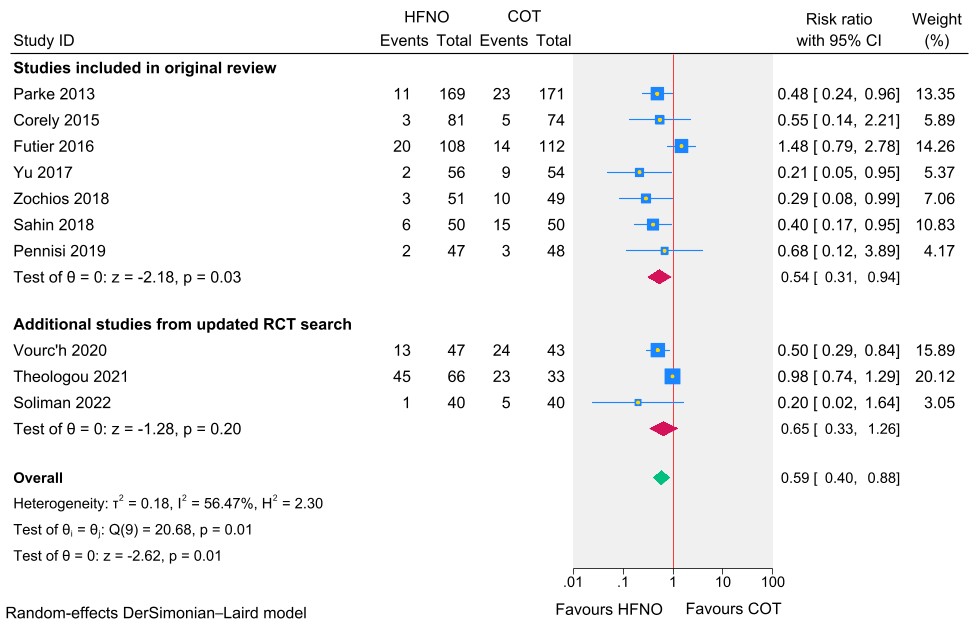


***Effect of HFNO on postoperative hypoxaemia when compared to conventional oxygen therapy***


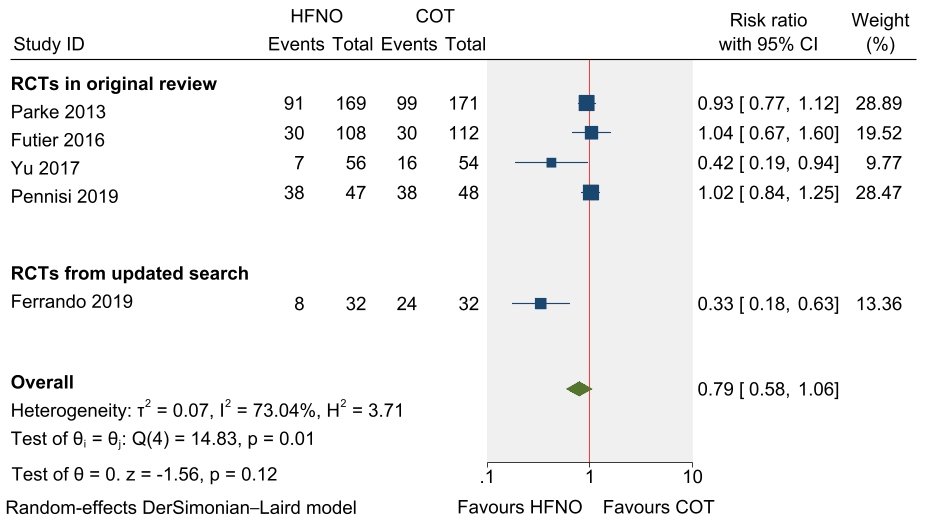


***Effect of HFNO on ICU length of stay when compared to conventional oxygen therapy***


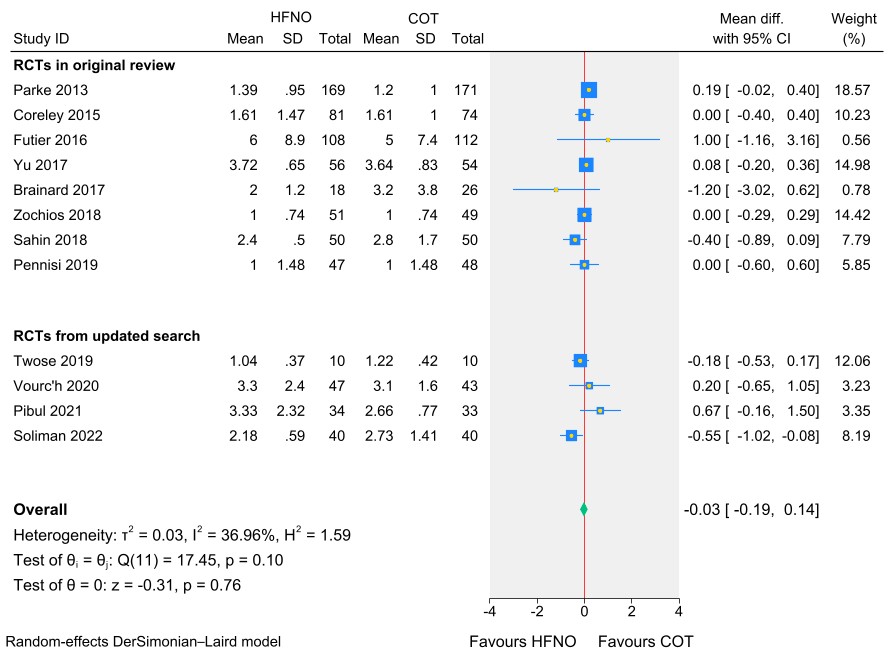

Supplement: Supplementary material 16 [file mmc16.docx]
