# Supplementary material for: Perioperative oxygen therapy: an overview of systematic reviews and meta-analyses
Source: Br J Anaesth. 2025 Jun 6;135(5):1456–76. doi: 10.1016/j.bja.2025.04.020 (PMC12597348; doi:10.1016/j.bja.2025.04.020)
Supplement: Supplementary material 17 [file mmc17.docx]

***Supplementary file 17***

***Subgroup analyses: HFNO vs COT_reintubation***

|  | **No. of  studies** | **No. of events in the intervention  group** | **No. of events in  the control group** | **Relative risk  (95% CI)** | **Test of group differences (P value)** |
| --- | --- | --- | --- | --- | --- |
| **Overall** | | | | | |
| All | 9 | 21/601 (3.5%) | 23/555 (4.1%) | 0.78 (0.29-2.07) | NA |
| **By type of surgery** | | | | | |
| Cardiac surgery | 7 | 20/498 (4%) | 17/453 (3.7%) | 0.94 (0.32-2.77) | 0.41 |
| Thoracic/ abdominal surgery | 2 | 1/103 (0.97%) | 6/102 (5.8%) | 0.31 (0.03-3.44) |  |
| **By risk of postoperative pulmonary complications** | | | | | |
| Average risk | 2 | 3/216 (1.4%) | 3/219 (1.3%) | 1.01 (0.21-4.97) | 0.69 |
| High risk | 7 | 18/385 (4.6%) | 20/336 (5.9%) | 0.67 (0.19-2.42) |  |
| **By BMI** | | | | | |
| Average BMI patients | 7 | 21/470 (4.4%) | 17/431 (3.9%) | 1.08 (0.39-3.03) | 0.09 |
| Obese patients | 2 | 0/131 | 6/124 (4.8%) | 0.14 (0.02-1.14) |  |

***Subgroup analyses: HFNO vs COT_escalation of respiratory support***

|  | **No. of  studies** | **No. of events in the intervention  group** | **No. of events in  the control group** | **Relative risk  (95% CI)** | **Test of group differences (P value)** |
| --- | --- | --- | --- | --- | --- |
| **Overall** | | | | | |
| All | 10 | 106 / 715 (15%) | 131/ 674 (19%) | 0.59 (0.40-0.88) | NA |
| **By type of surgery** | | | | | |
| Cardiac surgery | 6 | 81/464 (17%) | 100/420 (24%) | 0.56 (0.36-0.86) | 0.99 |
| Thoracic/ abdominal surgery | 4 | 25/251 (10%) | 31/254 (12%) | 0.55 (0.18-1.73) |  |
| **By risk of postoperative pulmonary complications** | | | | | |
| Average risk | 4 | 22/347 (6%) | 46/343 (13%) | 0.48 (0.29-0.77) | 0.46 |
| High risk | 6 | 84/368 (23%) | 85/331 (26%) | 0.63 (0.36-1.08) |  |
| **By BMI** | | | | | |
| Average BMI patients | 8 | 97/584 (17%) | 111/550 (20%) | 0.62 (0.39-0.97) | 0.43 |
| Obese patients | 2 | 9/131 (7%) | 20/124 (16%) | 0.44 (0.21-0.91) |  |
